# Supplementary material for: Effects of stimulus emotional content on gaze pattern: An eye-tracking study
Source: PLoS One. 2026 Jan 28;21(1):e0341261. doi: 10.1371/journal.pone.0341261 (PMC12851487; doi:10.1371/journal.pone.0341261)
Supplement: S1 File — (PDF) [file pone.0341261.s001.pdf]

Effects of stimulus emotional content on gaze pattern: an eye-tracking study

Supplementary method, code and data analyses

Andrés Castellanos-Chacón Daniela Arias-Otero Valeria Uribe-Jaramillo
Milena Vásquez-Amézquita Juan David Leongómez

05 November, 2025

Faculty of Psychology, Universidad El Bosque, Bogota, Colombia.
AC-Ch: gcastellanos@unbosque.edu.co.

Description

This document contains supplements to both the method and the data analysis in which it is contained, all code and step-by-step explanations of all analyses, figures and tables (including supplementary figures and tables) for:

Castellanos-Chacón, A., Arias-Otero, D., Uribe-Jaramillo, V., Leongómez, J. D. & Vásquez-Amézquita, M. (in prep). *Effects of stimulus emotional content on gaze pattern: an eye-tracking study.*

Data available from the Open Science Framework (OSF): https://doi.org/10.XXXXX/OSF.IO/XXXXX. All analyses were planned by Andrés Castellanos-Chacón, Daniela Arias-Otero, Valeria Uribe-Jaramillo, Juan David Leongómez y Milena Vásquez-Amézquita. This document and its underlying code were created in `R Markdown` by Andrés Castellanos-Chacón using LaTeX.

Contents

1 Method **2**
1.1 Stimuli and experimental paradigm . . . 2
1.1.1 Negative . . . 3
1.1.2 Positive . . . 3
1.1.3 Neutral . . . 3
1.1.4 Control . . . 3

2 Data analysis **3**
2.1 Preliminaries data analysis . . . 3
2.1.1 Load packages . . . 3
2.1.2 Custom functions . . . 4
2.1.2.1 `pval.stars` . . . 4
2.1.3 Load and wrangle data . . . 4
2.1.3.1 Load data base. . . . 4
2.1.3.2 Calculate first fixation count data . . . 5
2.2 Linear Mixed Models (LMM) . . . 5
2.2.1 Time to first fixation . . . 5
2.2.1.1 Fit model . . . 5
2.2.1.1.1 Model assumptions . . . 5
2.2.1.1.2 Summary model . . . 7

2.2.1.1.3 Estimated marginal means . . . 10
2.2.1.2 Figure . . . 11
2.2.1.3 Bootstrap . . . 12
2.2.2 First fixation duration . . . 14
2.2.2.1 Fit model . . . 14
2.2.2.1.1 Model assumptions . . . 14
2.2.2.1.2 Summary model . . . 16
2.2.2.1.3 Estimated marginal means . . . 19
2.2.2.2 Figure . . . 20
2.2.2.3 Bootstrap . . . 21
2.2.3 First fixation count . . . 23
2.2.3.1 Fit model . . . 23
2.2.3.1.1 Model assumptions . . . 23
2.2.3.1.2 Summary model . . . 25
2.2.3.1.3 Estimated marginal means . . . 27
2.2.3.2 Figure . . . 29
2.2.3.3 Bootstrap . . . 31
2.2.4 Total Duration of Fixation . . . 33
2.2.4.1 Fit model . . . 33
2.2.4.1.1 Model assumptions . . . 33
2.2.4.1.2 Summary model . . . 35
2.2.4.1.3 Estimated marginal means . . . 37
2.2.4.2 Figure . . . 38
2.2.4.3 Bootstrap . . . 39
2.2.5 Total number of fixations . . . 41
2.2.5.1 Fit model . . . 41
2.2.5.1.1 Model assumptions . . . 41
2.2.5.1.2 Summary model . . . 43
2.2.5.1.3 Estimated marginal means . . . 45
2.2.5.2 Figure . . . 46
2.2.5.3 Bootstrap . . . 47

3 Summary tables and figures 49

4 Session info (for reproducibility) 54

5 Supplementary references 55

1 Method

1.1 Stimuli and experimental paradigm

Seventy-one stimuli were originally preselected from the image banks the Nencki Affective Picture System, NAPS (Marchewka et al., 2014) and from the Open Affective Standardized Image Set, OASIS (Kurdi et al., 2017) using subjective valence scores on a scale of 1 to 9, with 1 being very negative and 9 being very positive, obtained in the validation of stimuli from the corresponding set: negative stimuli with scores below 3; positive stimuli with scores above 7; neutral stimuli of people and inanimate target control with scores between 4 and 5. Subsequently, the validation check of stimulus valence was performed on a sample similar to the one that would be evaluated in the final experiment and valence was evaluated on a pictographic scale from 4 (very pleasant) to -4 (very unpleasant) and arousal from 1 (not at all activating) to 9 (very activating) with a total of 213 participants, with mean ages of 21 to 34 (SD = 5.13). From these evaluations, we selected the stimuli that maintained the scores and that the participants also reported that they generated emotions such as: positive stimuli: “joy”, “happiness”; negative stimuli: “sadness”, “pain”, “fear”, “anger”, and neutral stimuli: “no emotion”. From this, the following stimuli were selected from each set:

1.1.1 Negative

-> OASIS (5): PN_1_Cemetery 5; PN_2_Miserable face 2; PN_7_Injury 3; PN_8_Sad face 9; PN_10_Tumor 1

-> NAPS (11): PN_13_People_039_v; PN_14_People_001_h; PN_15_People_003_h; PN_16_People_033_h; PN_18_People_226_h; PN_20_People_143_h; PN30_Faces_009_h; PN31_Faces_010_h; PN32_Faces_018_h; PN33_Faces_145_v; PN34_Faces_283_h PP_35_Faces_002_v

1.1.2 Positive

-> OASIS (8): PP_1_Astronaut 1; PP_3_Dancing 5; PP_7_Hang gliding 2; PP_8_Mother 8; PP_30_Mother 4; PP_31_Picnic 1; PP_32_Moter 7; PP_33_Father 1

-> NAPS (8): PP_11_People_174_v;PP_12_People_180_h;PP_13_People_176_h;PP_16_People_055_h; PP_17_People_172_v; PP_18_People_181_v; PP_34_Faces_001_h; PP_35_Faces_002_v.

1.1.3 Neutral

-> OASIS (6): PC_1_Bored pose 2; PC_2_Boxing 1; PC_3_Band 1; PC_6_Neutral face 5; PC_9_Scared face 1; PC_10_Sad face 4

-> NAPS (10): PC_16_People_092_v; PC_17_People_089_h; PC_18_People_091_h; PC_19_People_166_h; PC30_People_056_h; PC31_People_097_h; PC32_People_146_h; PC33_People_150_h; PC34_Faces_078_h; PC35_Faces_198_h

1.1.4 Control

-> OASIS (16): OC_2_Bed 1; OC_3_Bottle 1; OC_4_Cotton swabs 1; OC_5_Office supplies 4; OC_6_Office supplies 2; OC_10_Roofing 4; OC_11_Yarn 4; OC_12_Windmill 1; OC_14_Storage 2; OC_17_Cups 3; OC_19_Cotton swabs 3; OC_20_Fire hydrant 2; OC30_Phone 1; OC31_Cups 2; OC32_Fence 3; OC33_Rocks 3

2 Data analysis

2.1 Preliminaries data analysis

2.1.1 Load packages

This file was created using knitr (Xie, 2014), mostly following the tidyverse (Wickham et al., 2019) syntax. Data wrangling was primarily handled with dplyr (Wickham et al., 2022), and most figures were created or modified using ggplot2 (Wickham, 2016). Tables were generated using knitr::kable and enhanced with kableExtra (Zhu, 2020).

The dataset was imported using the readxl package (Wickham & Bryan, 2021). To generate summary tables combining multiple ANOVA results, DescTools (Signorell, 2023) was used. Additionally, tidyquant (Dancho & Vaughan, 2023) supported customization of the visual layout of the figures.

For statistical modeling, models 1, 2, 4, and 5 were fitted using the glmmTMB package (Brooks et al., 2017) to estimate generalized linear mixed models. Model 3, in contrast, was analyzed using a count-based GLMM with a Conway–Maxwell–Poisson (COM-Poisson) distribution, which provides a flexible approach for count data exhibiting under-dispersion. In all models, stimulus content and gender were included as fixed effects, and random intercepts were included for participants.

Model assumptions and diagnostics were checked using the performance package (Lüdecke et al., 2021) and DHARMa (Hartig, 2024). Estimated marginal means and contrasts were obtained using emmeans (Lenth, 2022). Output tidying and table formatting were supported by broom and broom.mixed (Robinson, 2020).

Graphs were refined using ggpubr (Kassambara, 2023), and significance annotations were added using gtools (Bolker et al., 2022). The tidyr (Wickham et al., 2023) package helped structure post hoc tables and contrasts.

To generate bootstrap confidence intervals, the boot (Canty & Ripley, 2022) package was used. External PDF files, such as the experimental design figure, were processed using magick, pdftools, and ggplotify.

Used packages also include `osfr` (Wolen et al., 2020) to download and open data files directly from the Open Science Framework (OSF), using the `osf_retrieve_file` and `osf_download` functions.

All packages used in this file can be directly installed from the Comprehensive R Archive Network (CRAN).

```
library(tidyverse)
library(dplyr)
library(lmerTest)
library(performance)
library(readxl)
library(emmeans)
library(kableExtra)
library(broom)
library(ggpubr)
library(gtools)
library(tidyr)
library(boot)
library(tidyquant)
library(magick)
library(ggplotify)
library(pdftools)
library(glmmTMB)
library(car)
library(broom.mixed)
library(DescTools)
library(DHARMa)
```

2.1.2 Custom functions

2.1.2.1 `pval.stars` This function takes p-values and adds starts to represent significance levels.

```
pval.stars <- function(pvals) {
  ifelse(pvals < 0.0001,
         "****",
         ifelse(pvals < 0.001,
                "***",
                ifelse(pvals < 0.01,
                "**",
                       ifelse(pvals < 0.05,
                       "*", NA))))
}
```

2.1.3 Load and wrangle data

2.1.3.1 Load data base. The first thing we do is to load the database and save it in a variable that we will call 'bd'. After that, we will check all the variables that are identified as 'character' and we will convert them into 'factor' type. And we will reorder the levels leaving the Neutral stimuli first, followed by the Control, Positive and Negative stimuli.

```
bd <-  read_excel("Data/Datos1.xlsx")|>
  mutate(across(where(is.character), as.factor)) |>
  mutate(Stimulus_content = fct_relevel(Stimulus_content,
                                        "Negative",
                                        "Positive",
                                        "Neutral",
```

```
                                    "Control" ))
```

2.1.3.2 Calculate first fixation count data This formula takes the values of the first fixation column and stores them in a variable so as not to repeat values due to the vertical database being handled.

```
pf <- bd |>
  group_by(Participant, First_fixation) |>
  count(First_fixation, .drop = FALSE) |>
  mutate(n = n/4) |>
  mutate(Gender = ifelse(grepl("H", Participant),
                         "Male", "Female"))|>
  mutate(First_fixation = fct_relevel(First_fixation,
                                      "Negative",
                                      "Positive",
                                      "Neutral",
                                      "Control" ))

pf <- pf |>
  filter(!(Participant %in% c("H035", "M011") & is.na(First_fixation)))
```

2.2 Linear Mixed Models (LMM)

Generalized Linear Mixed Models (GLMMs) extend Linear Mixed Models (LMMs) by allowing the response variable to follow a distribution other than the normal distribution, and by applying a link function to relate the linear predictor to the expected value of the response (Nakagawa & Schielzeth, 2013).

In the present analysis, GLMMs are used when the distributional assumptions of LMMs are not appropriate for the characteristics of the data.

2.2.1 Time to first fixation

The time to first fixation is understood as the participant's latency to the first stimulus, once the competing stimuli are presented. Previously, a cross has been presented so that the participant fixates his gaze on the center of the screen and has the same probability of seeing any stimulus.

2.2.1.1 Fit model In this model, the time to first fixation is predicted by the type of stimulus and its interaction with participant gender. Random intercepts are included for both participant and stimulus, and by-participant random slopes for stimulus content are added. The response variable is modeled using a Gamma distribution with a log link, which is appropriate for positive, right-skewed response times.

Zero values were discarded as they did not make sense for the measurement, a total of 3 out of 15315 measurements were discarded.

```
bd1 <- subset(bd, Time_first_fixation > 0)

mod1_glmm <- glmmTMB(Time_first_fixation ~ Stimulus_content * Gender +
                     (1 + Stimulus_content | Participant) +
                     (1 | Stimulus),
                     family = Gamma(link = "log"),
                     data = bd1)
```

2.2.1.1.1 Model assumptions The following functions 'check_model()', 'check_distribution()', and 'check_normality()' are used to evaluate the quality of fit and distribution of residuals in a statistical model. For example, check_model() could provide a visualization of residuals and diagnose

problems such as heteroscedasticity or bias in the model. check_distribution() could provide a visualization of the distribution of residuals and diagnose problems such as deviation from normality or outliers.

```
check_model(mod1_glmm)
```

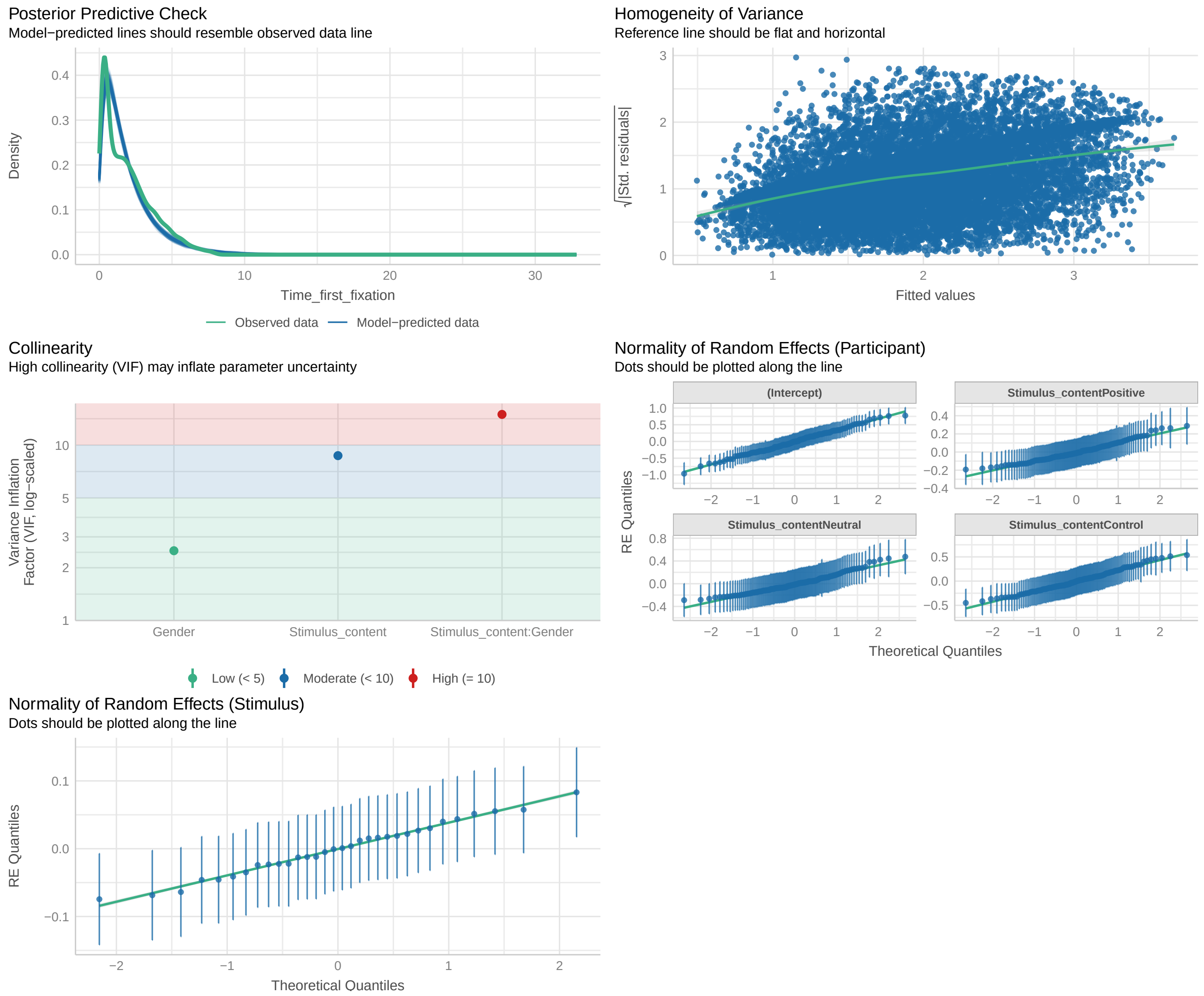


```
dist_checkmod1 <- check_distribution(mod1_glmm)
kable(data.frame(dist_checkmod1),
      digits = 2, booktabs = TRUE,
      caption = "Distribution of residuals model 1") %>%
      kable_styling(latex_options = c("HOLD_position"))
```

Table S1. *Distribution of residuals model 1*

Distribution	p_Residuals	p_Response
bernoulli	0.00	0.00
beta	0.00	0.06
beta-binomial	0.00	0.00
binomial	0.00	0.03
cauchy	0.19	0.00
chi	0.06	0.00
exponential	0.03	0.19
F	0.00	0.00
gamma	0.06	0.16
half-cauchy	0.03	0.00
inverse-gamma	0.00	0.00
lognormal	0.00	0.00
neg. binomial (zero-infl.)	0.00	0.00
negative binomial	0.00	0.00
normal	0.44	0.00
pareto	0.00	0.00
poisson	0.00	0.00
poisson (zero-infl.)	0.00	0.00
tweedie	0.16	0.31
uniform	0.00	0.00
weibull	0.03	0.25

2.2.1.1.2 Summary model To examine the results of model 1, we used three complementary summaries. First, we used the Anova() function from the car package to obtain a Type III Wald chi-square test, which evaluates the significance of each predictor and their interaction in the model. This method is appropriate for generalized linear mixed models fitted with glmmTMB.

Second, we applied the summary() function to obtain the estimated coefficients, their standard errors, z-values, and associated p-values for the fixed effects.

Third, we calculated the marginal and conditional pseudo R^2 values using the r2_nakagawa() function. These values reflect the proportion of variance explained by the fixed effects alone (marginal R^2) and by both fixed and random effects combined (conditional R^2), following the method proposed by Nakagawa and Schielzeth (Nakagawa & Schielzeth, 2013).

In addition to analyzing the fixed effects, the model output included a detailed summary of the random effects structure, which captures individual and stimulus-level variability. Specifically, we report the standard deviations and variances of the random intercepts and slopes, along with their correlations.

```
anova.mod1 <- Anova(mod1_glmm, type = 3)
anova.mod1 <- as.data.frame(anova.mod1)|>
  rownames_to_column() |>
  mutate_at("rowname", str_replace_all, ":", " × ") |>
  mutate_at("rowname", str_replace_all, "Stimulus_content",
          "Stimulus content")|>
  rename( Effect = rowname)|>
  kable(digits = 2, booktabs = TRUE,
        align = c("l", "l", rep("c", 4)), caption = "Effect of Stimulus
        Content and Participant's Gender on Time to First Fixation (TFF)",
        escape = FALSE)|>
        kable_styling(latex_options = c("hold_position"))
anova.mod1
```

Table S2. *Effect of Stimulus Content and Participant's Gender on Time to First Fixation (TFF)*

Effect	Chisq	Df	Pr(>Chisq)
(Intercept)	59.47	1	0.00
Stimulus content	97.55	3	0.00
Gender	0.91	1	0.34
Stimulus content × Gender	1.75	3	0.62

```
sum.mod1 <- summary(mod1_glmm)


sum.mod1_df <- tidy(mod1_glmm, effects = "fixed") |>
  mutate(term = str_replace_all(term, ":", " × ")) |>
  mutate(term = str_replace_all(term, "Stimulus_contentControl",
                               "Stimulus content [Control]")) |>
  mutate(term = str_replace_all(term, "Stimulus_contentPositive",
                               "Stimulus content [Positive]")) |>
  mutate(term = str_replace_all(term, "Stimulus_contentNeutral",
                               "Stimulus content [Neutral]")) |>
  mutate(term = str_replace_all(term, "GenderMale",
                               "Gender [Male]")) |>
  rename(Effect = term,
         Estimate = estimate,
         `Std. Error` = std.error,
         `z value` = statistic,
         `Pr(>|z|)` = p.value)|>
  select(Effect, Estimate, `Std. Error`, `z value`, `Pr(>|z|)`)

sum.mod1_df |>
  kable(digits = 2, booktabs = TRUE,
        align = c("l", rep("c", 5)),
        caption = "Time to First Fixation (TFF) by stimulus content, gender, and their interaction",
        escape = FALSE) |>
  kable_styling(latex_options = c("HOLD_position"))
```

Table S3. *Time to First Fixation (TFF) by stimulus content, gender, and their interaction*

Effect	Estimate	Std. Error	z value	Pr(>|z|)
(Intercept)	0.40	0.05	7.71	0.00
Stimulus content [Positive]	0.19	0.03	5.69	0.00
Stimulus content [Neutral]	0.28	0.04	6.91	0.00
Stimulus content [Control]	0.44	0.04	9.82	0.00
Gender [Male]	-0.07	0.07	-0.95	0.34
Stimulus content [Positive] × Gender [Male]	0.04	0.05	0.80	0.43
Stimulus content [Neutral] × Gender [Male]	0.02	0.05	0.30	0.76
Stimulus content [Control] × Gender [Male]	0.06	0.06	1.04	0.30

```
r2mod1 <- r2_nakagawa(mod1_glmm)
r2_tblmod1 <- as_tibble(r2mod1)
kable(r2_tblmod1, format = "markdown",  booktabs =TRUE,
      align = "c", caption = "R model 1",
      escape = FALSE) %>%
      kable_styling(latex_options = c("HOLD_position"))
```

Table S4. *R model 1*

R2_conditional	R2_marginal
0.1698319	0.0433354

```r
re_par <- parameters::model_parameters(mod1_glmm, effects = "random")
re_df  <- as.data.frame(re_par)

col_param <- intersect(c("Parameter","Term","Name"), names(re_df))[1]
col_est   <- intersect(c("SD","SD (random effect)","Coefficient","Estimate","Est."), names(re_df))[1]
col_group <- intersect(c("Group","Cluster"), names(re_df))[1]

tab_sd <- re_df %>%
  transmute(
    Group  = .data[[col_group]],
    Effect_raw = .data[[col_param]],
    SD   = suppressWarnings(as.numeric(.data[[col_est]]))
  ) %>%
  filter(grepl("^SD\\s*\\(", Effect_raw)) %>%
  mutate(
    Effect = str_replace(Effect_raw, "^SD\\s*\\((.*)\\)$", "\\1"),
    Effect = str_replace_all(Effect, "Stimulus_content", "Stimulus content"),
    Effect = str_replace_all(Effect, "First_fixation",   "Stimulus content"),
    Effect = str_replace_all(Effect, ":", " × "),
    Effect = str_replace(Effect, "Stimulus contentPositive", "Stimulus content [Positive]"),
    Effect = str_replace(Effect, "Stimulus contentNeutral",  "Stimulus content [Neutral]"),
    Effect = str_replace(Effect, "Stimulus contentControl",  "Stimulus content [Control]"),
    Variance = SD^2
  ) %>%
  select(Group, Effect, SD, Variance)

kable(tab_sd, digits = 4, booktabs = TRUE,
      caption = "Random effects (SD and Variance) - Model 1 (Gamma GLMM)") %>%
  kable_styling(latex_options = c("HOLD_position"))
```

Table S5. *Random effects (SD and Variance) — Model 1 (Gamma GLMM)*

Group	Effect	SD	Variance
Participant	Intercept	0.3611	0.1304
Stimulus	Intercept	0.0495	0.0025
Participant	Stimulus content [Positive]	0.1298	0.0169
Participant	Stimulus content [Neutral]	0.2100	0.0441
Participant	Stimulus content [Control]	0.2582	0.0667

```r
vc <- VarCorr(mod1_glmm)
part_cov <- try(as.matrix(vc$cond$Participant), silent = TRUE)

if (!inherits(part_cov, "try-error")) {
  part_cor <- cov2cor(part_cov)

  rn <- rownames(part_cor)
  cn <- colnames(part_cor)
  cor_long <- as.data.frame(as.table(part_cor), stringsAsFactors = FALSE) %>%
    rename(Effect1 = Var1, Effect2 = Var2, Correlation = Freq) %>%
```

```
    mutate(
      i = match(Effect1, rn),
      j = match(Effect2, cn)
    ) %>%
    filter(i < j) %>%
    select(-i, -j)


  pretty <- function(x) {
    x %>%
      str_replace_all("Stimulus_content", "Stimulus content") %>%
      str_replace_all("First_fixation",   "Stimulus content") %>%
      str_replace_all(":", " × ") %>%
      str_replace("^\\(Intercept\\)$", "Intercept") %>%
      str_replace("Stimulus contentPositive", "Stimulus content [Positive]") %>%
      str_replace("Stimulus contentNeutral",  "Stimulus content [Neutral]") %>%
      str_replace("Stimulus contentControl",  "Stimulus content [Control]")
  }

  cor_tab <- cor_long %>%
    mutate(
      Effect1 = pretty(Effect1),
      Effect2 = pretty(Effect2)
    ) %>%
    transmute(
      Group = "Participant",
      Effect = paste0(Effect1, " × ", Effect2),
      Correlation
    )

  kable(cor_tab, digits = 3, booktabs = TRUE,
        caption = "Random-effects correlations (Participant level) - Model 1") %>%
    kable_styling(latex_options = c("HOLD_position"))
} else {
  kable(data.frame(Note = "Correlations could not be extracted from VarCorr(mod1_glmm)."),
        booktabs = TRUE,
        caption = "Random-effects correlations - Model 1") %>%
    kable_styling(latex_options = c("HOLD_position"))

}
```

Table S6. *Random-effects correlations (Participant level) — Model 1*

Group	Effect	Correlation
Participant	Intercept × Stimulus content [Positive]	-0.723
Participant	Intercept × Stimulus content [Neutral]	-0.660
Participant	Stimulus content [Positive] × Stimulus content [Neutral]	0.996
Participant	Intercept × Stimulus content [Control]	-0.838
Participant	Stimulus content [Positive] × Stimulus content [Control]	0.983
Participant	Stimulus content [Neutral] × Stimulus content [Control]	0.963

2.2.1.1.3 Estimated marginal means Given the significant effect observed for the stimulus content variable in the ANOVA-type table, post-hoc pairwise comparisons were performed using the emmeans() function. These contrasts allow for a detailed examination of the differences between levels of Stimulus content (i.e., Control, Neutral, Positive, Negative), adjusting for multiple comparisons and

accounting for the model's link function.

The estimated marginal means were computed on the response scale using type = "response" and regridded to obtain interpretable values (i.e., in milliseconds). The table below presents the pairwise contrasts between levels of Stimulus content, where the estimate reflects the difference in expected time to first fixation between the compared levels.

```
mcm1 <- emmeans(mod1_glmm, pairwise ~ Stimulus_content, type = "response")
mcm1$emmeans <- regrid(mcm1$emmeans)
mcm1$contrasts <- pairs(mcm1$emmeans)

mcm1_emmeans <- tibble(data.frame(mcm1$emmeans)) |>
  rename(Time_first_fixation = response)|>
  select(-df)

contrasts_df1 <-  as.data.frame(mcm1$contrasts)|>
  select(-df)


kable(contrasts_df1, digits = 5,  booktabs =TRUE,
      align = "c", caption = "contrasts between variables, according to the
      content of the stimulus of model 1") %>%
      kable_styling(latex_options = "HOLD_position",
                font_size = 12,
                full_width = FALSE)
```

Table S7. *contrasts between variables, according to the content of the stimulus of model 1*

contrast	estimate	SE	z.ratio	p.value
Negative - Positive	-0.33429	0.03601	-9.28333	0.00000
Negative - Neutral	-0.47461	0.04507	-10.53027	0.00000
Negative - Control	-0.86236	0.05195	-16.59891	0.00000
Positive - Neutral	-0.14032	0.03954	-3.54898	0.00219
Positive - Control	-0.52807	0.04635	-11.39245	0.00000
Neutral - Control	-0.38775	0.04349	-8.91614	0.00000

2.2.1.2 Figure Finally, for this model 1, a figure is shown in which the time for the first fixation is observed according to the content of the stimulus and the respective differences between the variables are presented.

```
tbl.contrasts_df1 <- contrasts_df1 %>%
  mutate(.y. = "Time_first_fixation") %>%
  separate_wider_delim(contrast, " - ",
                       names = c("group1", "group2")) %>%
  select(7, 1:6) %>%
  mutate(p.signif = pval.stars(p.value))


fig_TPF <- ggplot(bd, aes(x=Stimulus_content,
               y=Time_first_fixation)) +
  geom_violin(trim = FALSE,
              aes(fill = Stimulus_content)) +
  geom_jitter(alpha = 0.0009, width = 0.3) +
  geom_errorbar(data = mcm1_emmeans,
              mapping =
```

```
                aes(ymin = Time_first_fixation-SE,
                    ymax = Time_first_fixation+SE),
                colour = "black", width = 0.1) +
  geom_point(data = mcm1_emmeans,
             size = 1,
             color = "black", fill = "white") +
  stat_pvalue_manual(tbl.contrasts_df1,
                     label = "p.signif",
                     y.position =  c(10, 12, 14, 11, 13, 10))  +
  labs(x = "Stimulus Content",
       y = "Time to first fixation (TFF)",
       fill = " ") +
  theme(axis.text.x = element_text(size = 10))+
  guides(fill = FALSE)+
  theme_tq()


fig_TPF
```

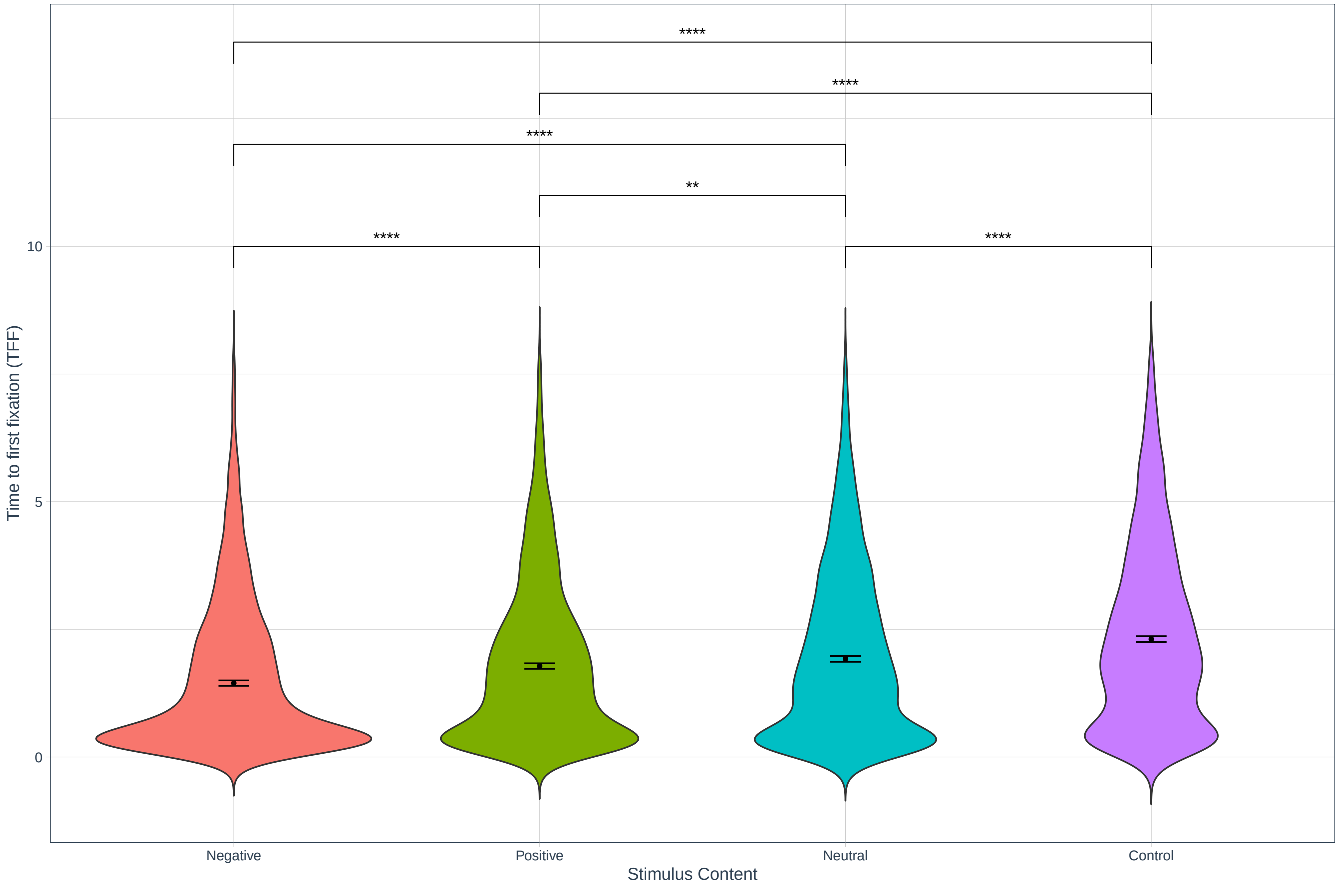

Figure S1. *Dimension between the emotional content of the stimulus, images with negative, positive, neutral and control emotional content, see Table S7, where the contrasts between each dimension are observed. In all cases, significant effects are represented with lines and stars:* *p < .05, **p < .01, ***p < .001, ****p < .0001.

2.2.1.3 Bootstrap Given that the model is a generalized linear mixed model with a non-normal error distribution (Gamma with log link), standard inferential techniques based on asymptotic normality may not provide reliable estimates. Therefore, a non-parametric bootstrap approach was used to better

estimate the uncertainty associated with the fixed effects.

The bootstrap method involves generating multiple resamples from the original dataset (with replacement) and fitting the same model on each sample. This process produces an empirical sampling distribution of the statistic of interest, in this case, the Time to first fixation (TFF).

```
boot_fn <- function(data, indices) {
  d <- data[indices, ]
  fit <- glmmTMB(Time_first_fixation ~ Stimulus_content * Gender +
                   (1 + Stimulus_content | Participant) +
                   (1 | Stimulus),
                 data = d, family = Gamma(link = "log"))
  return(fixef(fit)$cond)
}

set.seed(824)
mod1_boot <- boot(data = bd1, statistic = boot_fn, R = 1000)

boot.ci <- boot.ci(mod1_boot, type = "basic", index = 1)
terms <- names(fixef(mod1_glmm)$cond)
boots_mod1_df <- data.frame(
  term = terms,
  observed = fixef(mod1_glmm)$cond,
  rep.mean = colMeans(mod1_boot$t),
  se = apply(mod1_boot$t, 2, sd),
  bias = colMeans(mod1_boot$t) - fixef(mod1_glmm)$cond
)

get_ci <- function(boot_obj, index) {
  ci <- boot.ci(boot_obj, type = "basic", index = index)
  if (!is.null(ci$basic)) {
    return(c(lower = ci$basic[4], upper = ci$basic[5]))
  } else {
    return(c(lower = NA, upper = NA))
  }
}


boot_cis <- t(sapply(1:length(terms), function(i) get_ci(mod1_boot, i)))
boots_mod1_df$ci.lower <- boot_cis[, "lower"]
boots_mod1_df$ci.upper <- boot_cis[, "upper"]


boots_mod1_df <- boots_mod1_df |>
  mutate(term = str_replace_all(term, ":", " × ")) |>
  mutate(term = str_replace_all(term, "Stimulus_contentControl", "Stimulus content [Control]")) |>
  mutate(term = str_replace_all(term, "Stimulus_contentPositive", "Stimulus content [Positive]")) |>
  mutate(term = str_replace_all(term, "Stimulus_contentNeutral", "Stimulus content [Neutral]")) |>
  mutate(term = str_replace_all(term, "GenderMale", "Gender [Male]"))

orden_deseado <- c("(Intercept)",
                   "Stimulus content [Positive]",
                   "Stimulus content [Neutral]",
                   "Stimulus content [Control]" ,
                   "Gender [Male]",
                   "Stimulus content [Positive] × Gender [Male]",
                   "Stimulus content [Neutral] × Gender [Male]",
```

```
                   "Stimulus content [Control] × Gender [Male]")

boots_mod1_df <- boots_mod1_df |> arrange(match(term, orden_deseado))
rownames(boots_mod1_df) <- NULL

boots_mod1_df |>
  select(term, observed, rep.mean, se, bias, ci.lower, ci.upper) |>
  rename(
    Effect = term,
    Estimate = observed,
    `Boot Mean` = rep.mean,
    `Std. Error` = se,
    Bias = bias,
    `CI Lower` = ci.lower,
    `CI Upper` = ci.upper
  ) |>
  kable(digits = 4, booktabs = TRUE,
        caption = "Bootstrap estimates of model 1",
        align = "lcccccc",
         rownames = FALSE) |>
  kable_styling(latex_options = c("HOLD_position"))
```

Table S8. *Bootstrap estimates of model 1*

Effect	Estimate	Boot Mean	Std. Error	Bias	CI Lower	CI Upper
(Intercept)	0.4031	0.3919	0.0203	-0.0112	0.3716	0.4523
Stimulus content [Positive]	0.1897	0.1966	0.0280	0.0069	0.1314	0.2376
Stimulus content [Neutral]	0.2754	0.2805	0.0281	0.0051	0.2170	0.3276
Stimulus content [Control]	0.4356	0.4403	0.0253	0.0047	0.3809	0.4812
Gender [Male]	-0.0678	-0.0686	0.0281	-0.0008	-0.1212	-0.0130
Stimulus content [Positive] × Gender [Male]	0.0365	0.0353	0.0380	-0.0012	-0.0329	0.1136
Stimulus content [Neutral] × Gender [Male]	0.0167	0.0157	0.0384	-0.0010	-0.0574	0.0956
Stimulus content [Control] × Gender [Male]	0.0639	0.0639	0.0358	0.0000	-0.0054	0.1314

2.2.2 First fixation duration

It is a measure of early attention and its objective is to capture the total time a person spends looking at a stimulus the first time he/she directs his/her gaze to the target.

2.2.2.1 Fit model To predict the first fixation duration, a generalized linear mixed model (GLMM) with a Gamma distribution and log link was fitted. The fixed effects included the stimulus content, participant gender, and their interaction. Random intercepts were added for both participant and stimulus, and a random slope for stimulus content was included for each participant. This accounts for individual variability in baseline fixation duration and how different stimulus types affect participants differently.

```
mod2_glmm <- glmmTMB(First_fix_duration ~ Stimulus_content * Gender +
                     (1 + Stimulus_content| Participant) +
                     (1 | Stimulus),
                     family = Gamma(link = "log"),
                     data = bd1)
```

2.2.2.1.1 Model assumptions The following functions ‘check_model()’, ‘check_distribution()’, and ‘check_normality()’ are used to evaluate the quality of fit and distribution of residuals in a statistical model. For example, check_model() could provide a visualization of residuals and diagnose

problems such as heteroscedasticity or bias in the model. check_distribution() could provide a visualization of the distribution of residuals and diagnose problems such as deviation from normality or outliers.

```
check_model(mod2_glmm)
```

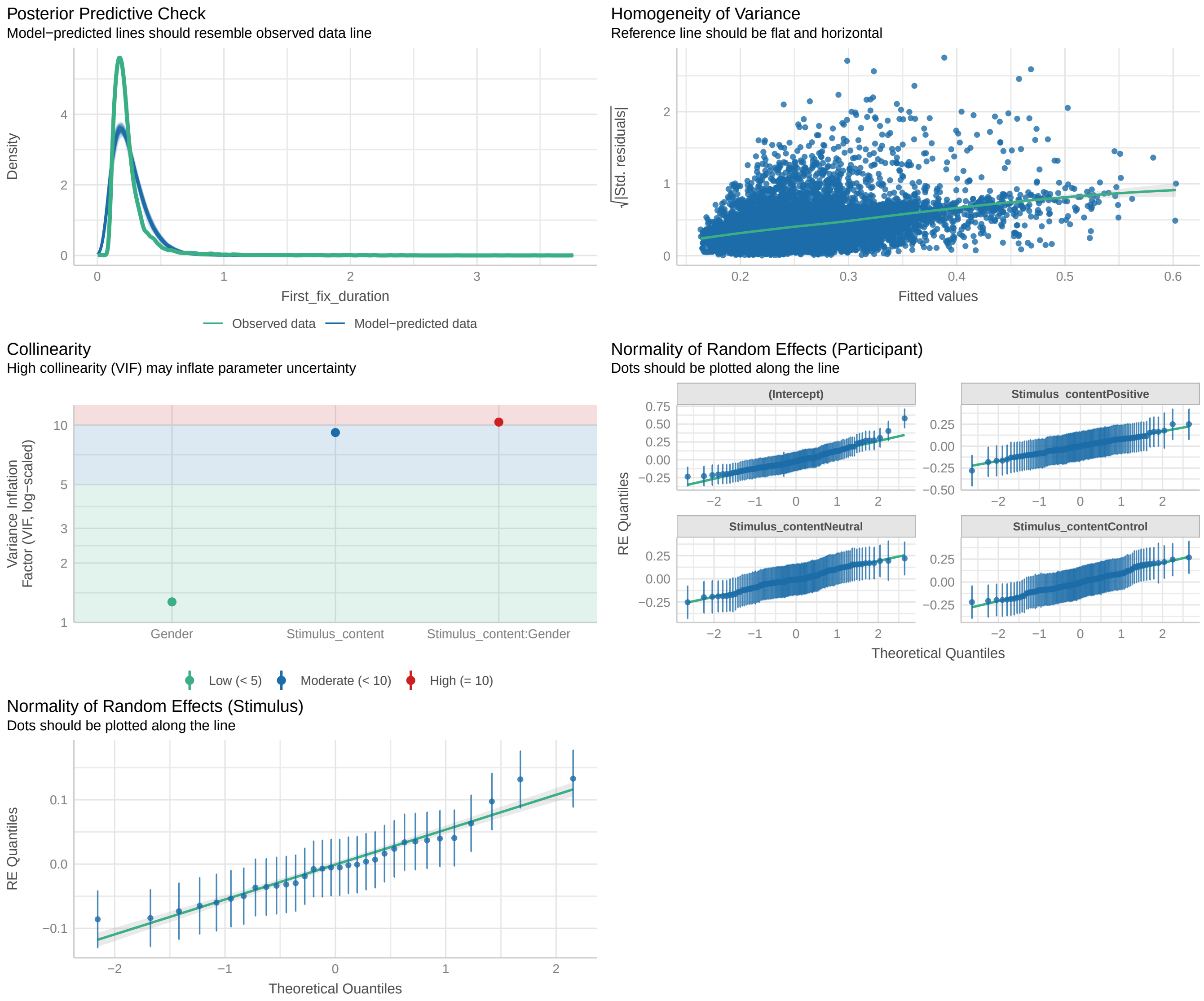


```
dist_checkmod2 <- check_distribution(mod2_glmm)
      kable(data.frame(dist_checkmod2),
      digits = 2, booktabs = TRUE,
      caption = "Distribution of residuals model 2") %>%
      kable_styling(latex_options = c("HOLD_position"))
```

Table S9. *Distribution of residuals model 2*

Distribution	p_Residuals	p_Response
bernoulli	0.00	0.00
beta	0.03	0.06
beta-binomial	0.00	0.03
binomial	0.00	0.06
cauchy	0.50	0.00
chi	0.00	0.00
exponential	0.03	0.00
F	0.00	0.00
gamma	0.28	0.09
half-cauchy	0.00	0.00
inverse-gamma	0.09	0.56
lognormal	0.00	0.00
neg. binomial (zero-infl.)	0.00	0.00
negative binomial	0.00	0.00
normal	0.06	0.00
pareto	0.00	0.00
poisson	0.00	0.03
poisson (zero-infl.)	0.00	0.03
tweedie	0.00	0.12
uniform	0.00	0.00
weibull	0.00	0.00

2.2.2.1.2 Summary model To examine the results of model 2, we used three complementary summaries. First, we used the Anova() function from the car package to obtain a Type III Wald chi-square test, which evaluates the significance of each predictor and their interaction in the model. This method is appropriate for generalized linear mixed models fitted with glmmTMB.

Second, we applied the summary() function to obtain the estimated coefficients, their standard errors, z-values, and associated p-values for the fixed effects.

Third, we calculated the marginal and conditional pseudo R^2 values using the r2_nakagawa() function. These values reflect the proportion of variance explained by the fixed effects alone (marginal R^2) and by both fixed and random effects combined (conditional R^2), following the method proposed by Nakagawa and Schielzeth (Nakagawa & Schielzeth, 2013).

```
anova.mod2 <- Anova(mod2_glmm, type = 3)
anova.mod2 <- as.data.frame(anova.mod2)|>
  rownames_to_column() |>
  mutate_at("rowname", str_replace_all, ":", " × ") |>
  mutate_at("rowname", str_replace_all, "Stimulus_content",
        "Stimulus content")|>
  rename( Effect = rowname)|>
  kable(digits = 2, booktabs = TRUE,
        align = c("l", "l", rep("c", 4)), caption = "Effect of Stimulus
        Content and Participant's Gender on Duration of first
        Fixation (FFD)",
        escape = FALSE)|>
        kable_styling(latex_options = c("hold_position"))
anova.mod2
```

```
sum.mod2 <- summary(mod2_glmm)
```

Table S10. *Effect of Stimulus Content and Participant's Gender on Duration of first Fixation (FFD)*

Effect	Chisq	Df	Pr(>Chisq)
(Intercept)	3266.08	1	0.00
Stimulus content	67.88	3	0.00
Gender	1.73	1	0.19
Stimulus content × Gender	3.01	3	0.39

```
sum.mod2_df <- tidy(mod2_glmm, effects = "fixed") |>
  mutate(term = str_replace_all(term, ":", " × ")) |>
  mutate(term = str_replace_all(term, "Stimulus_contentControl",
                               "Stimulus content [Control]")) |>
  mutate(term = str_replace_all(term, "Stimulus_contentPositive",
                               "Stimulus content [Positive]")) |>
  mutate(term = str_replace_all(term, "Stimulus_contentNeutral",
                               "Stimulus content [Neutral]")) |>
  mutate(term = str_replace_all(term, "GenderMale",
                               "Gender [Male]")) |>
  rename(Effect = term,
         Estimate = estimate,
         `Std. Error` = std.error,
         `z value` = statistic,
         `Pr(>|z|)` = p.value)|>
  select(Effect, Estimate, `Std. Error`, `z value`, `Pr(>|z|)`)

sum.mod2_df |>
  kable(digits = 2, booktabs = TRUE,
        align = c("l", rep("c", 5)),
        caption = "Duration of first
        Fixation (FFD) by stimylus content,
        gender and the interaction
        between stimulus contentand gender",
        escape = FALSE) |>
  kable_styling(latex_options = c("HOLD_position"))
```

Table S11. *Duration of first Fixation (FFD) by stimylus content, gender and the interaction between stimulus contentand gender*

Effect	Estimate	Std. Error	z value	Pr(>|z|)
(Intercept)	-1.42	0.02	-57.15	0.00
Stimulus content [Positive]	-0.01	0.02	-0.54	0.59
Stimulus content [Neutral]	0.03	0.02	1.36	0.18
Stimulus content [Control]	0.15	0.02	6.53	0.00
Gender [Male]	-0.04	0.03	-1.32	0.19
Stimulus content [Positive] × Gender [Male]	0.01	0.03	0.48	0.63
Stimulus content [Neutral] × Gender [Male]	-0.02	0.03	-0.76	0.45
Stimulus content [Control] × Gender [Male]	0.02	0.03	0.64	0.52

```
r2mod2 <- r2_nakagawa(mod2_glmm)
r2_tblmod2 <- as_tibble(r2mod2)
kable(r2_tblmod2, format = "markdown",  booktabs =TRUE,
      align = "c", caption = "R model 2",
      escape = FALSE) %>%
```

```
    kable_styling(latex_options = c("HOLD_position"))
```

Table S12. *R model 2*

R2_conditional	R2_marginal
0.1601726	0.0224862

```
re_par2 <- parameters::model_parameters(mod2_glmm, effects = "random")
re_df2  <- as.data.frame(re_par2)

col_param2 <- intersect(c("Parameter","Term","Name"), names(re_df2))[1]
col_est2   <- intersect(c("SD","SD (random effect)","Coefficient","Estimate","Est."), names(re_df2))[1]
col_group2 <- intersect(c("Group","Cluster"), names(re_df2))[1]

tab_sd2 <- re_df2 %>%
  transmute(
    Group      = .data[[col_group2]],
    Effect_raw = .data[[col_param2]],
    SD         = suppressWarnings(as.numeric(.data[[col_est2]]))
  ) %>%
  filter(grepl("^SD\\s*\\(", Effect_raw)) %>%
  mutate(
    Effect = str_replace(Effect_raw, "^SD\\s*\\((.*)\\)$", "\\1"),
    Effect = str_replace_all(Effect, "Stimulus_content", "Stimulus content"),
    Effect = str_replace_all(Effect, "First_fixation",   "Stimulus content"),
    Effect = str_replace_all(Effect, ":", " × "),
    Effect = str_replace(Effect, "Stimulus contentPositive", "Stimulus content [Positive]"),
    Effect = str_replace(Effect, "Stimulus contentNeutral",  "Stimulus content [Neutral]"),
    Effect = str_replace(Effect, "Stimulus contentControl",  "Stimulus content [Control]"),
    Variance = SD^2
  ) %>%
  select(Group, Effect, SD, Variance)

kable(tab_sd2, digits = 4, booktabs = TRUE,
      caption = "Random effects (SD and Variance) - Model 2 (Gamma GLMM)") %>%
  kable_styling(latex_options = c("HOLD_position"))
```

Table S13. *Random effects (SD and Variance) — Model 2 (Gamma GLMM)*

Group	Effect	SD	Variance
Participant	Intercept	0.1501	0.0225
Stimulus	Intercept	0.0587	0.0034
Participant	Stimulus content [Positive]	0.1180	0.0139
Participant	Stimulus content [Neutral]	0.1281	0.0164
Participant	Stimulus content [Control]	0.1354	0.0183

```
vc2 <- VarCorr(mod2_glmm)
part_cov2 <- try(as.matrix(vc2$cond$Participant), silent = TRUE)

if (!inherits(part_cov2, "try-error")) {
  part_cor2 <- cov2cor(part_cov2)

  rn <- rownames(part_cor2)
  cn <- colnames(part_cor2)
```

```r
  cor_long <- as.data.frame(as.table(part_cor2), stringsAsFactors = FALSE) %>%
    rename(Effect1 = Var1, Effect2 = Var2, Correlation = Freq) %>%
    mutate(
      i = match(Effect1, rn),
      j = match(Effect2, cn)
    ) %>%
    filter(i < j) %>%
    select(-i, -j)


  pretty <- function(x) {
    x %>%
      str_replace_all("Stimulus_content", "Stimulus content") %>%
      str_replace_all("First_fixation",   "Stimulus content") %>%
      str_replace_all(":", " × ") %>%
      str_replace("^\\(Intercept\\)$", "Intercept") %>%
      str_replace("Stimulus contentPositive", "Stimulus content [Positive]") %>%
      str_replace("Stimulus contentNeutral",  "Stimulus content [Neutral]") %>%
      str_replace("Stimulus contentControl",  "Stimulus content [Control]")
  }

  cor_tab2 <- cor_long %>%
    mutate(
      Effect1 = pretty(Effect1),
      Effect2 = pretty(Effect2)
    ) %>%
    transmute(
      Group = "Participant",
      Effect = paste0(Effect1, " × ", Effect2),
      Correlation
    )

  kable(cor_tab2, digits = 3, booktabs = TRUE,
        caption = "Random-effects correlations (Participant level) - Model 2") %>%
    kable_styling(latex_options = c("HOLD_position"))
} else {
  kable(data.frame(Note = "Correlations could not be extracted from VarCorr(mod2_glmm)."),
        booktabs = TRUE,
        caption = "Random-effects correlations - Model 2") %>%
    kable_styling(latex_options = c("HOLD_position"))

}
```

Table S14. *Random-effects correlations (Participant level) — Model 2*

Group	Effect	Correlation
Participant	Intercept × Stimulus content [Positive]	-0.346
Participant	Intercept × Stimulus content [Neutral]	-0.153
Participant	Stimulus content [Positive] × Stimulus content [Neutral]	0.704
Participant	Intercept × Stimulus content [Control]	-0.105
Participant	Stimulus content [Positive] × Stimulus content [Control]	0.592
Participant	Stimulus content [Neutral] × Stimulus content [Control]	0.652

2.2.2.1.3 Estimated marginal means Given the significant effect observed for the stimulus content variable in the ANOVA-type table, post-hoc pairwise comparisons were performed using the

emmeans() function. These contrasts allow for a detailed examination of the differences between levels of Stimulus content (i.e., Control, Neutral, Positive, Negative), adjusting for multiple comparisons and accounting for the model's link function.

The estimated marginal means were computed on the response scale using type = "response" and regridded to obtain interpretable values (i.e., in milliseconds). The table below presents the pairwise contrasts between levels of Stimulus content.

```
mcm2 <- emmeans(mod2_glmm, pairwise ~ Stimulus_content, type = "response")
mcm2$emmeans <- regrid(mcm2$emmeans)
mcm2$contrasts <- pairs(mcm2$emmeans)

mcm2_emmeans <- tibble(data.frame(mcm2$emmeans)) |>
  rename(First_fix_duration = response)|>
  select(-df)

contrasts_df2 <-  as.data.frame(mcm2$contrasts)|>
  select(-df)


kable(contrasts_df2, digits = 5,  booktabs =TRUE,
      align = "c", caption = "contrasts between variables, according to the
      content of the stimulus of model 2") %>%
      kable_styling(latex_options = "HOLD_position",
                font_size = 12,
                full_width = FALSE)
```

Table S15. *contrasts between variables, according to the content of the stimulus of model 2*

contrast	estimate	SE	z.ratio	p.value
Negative - Positive	0.00109	0.00359	0.30507	0.99015
Negative - Neutral	-0.00455	0.00380	-1.19723	0.62859
Negative - Control	-0.04251	0.00444	-9.57112	0.00000
Positive - Neutral	-0.00565	0.00331	-1.70464	0.32117
Positive - Control	-0.04360	0.00411	-10.61099	0.00000
Neutral - Control	-0.03795	0.00395	-9.61636	0.00000

2.2.2.2 Figure Finally, for this model 2, a figure is shown in which the time for First fixation duration is observed according to the content of the stimulus and the respective differences between the variables are presented.

```
tbl.contrasts_df2 <- contrasts_df2 %>%
  mutate(.y. = "First_fix_duration") %>%
  separate_wider_delim(contrast, " - ",
                      names = c("group1", "group2")) %>%
  select(7, 1:6) %>%
  mutate(p.signif = pval.stars(p.value))

fig_DFF <- ggplot(bd, aes(x=Stimulus_content,
               y=First_fix_duration)) +
  geom_violin(trim = FALSE,
              aes(fill = Stimulus_content)) +
  geom_jitter(alpha = 0.0009, width = 0.3) +
  geom_errorbar(data = mcm2_emmeans,
              mapping =
```

```
                  aes(ymin = First_fix_duration-SE,
                      ymax = First_fix_duration+SE),
                  colour = "black", width = 0.1) +
  geom_point(data = mcm2_emmeans,
             shape = 50, size = 1,
             color = "black", fill = "white") +
  stat_pvalue_manual(tbl.contrasts_df2,
                     label = "p.signif",
                     y.position =  c(2.5, 2.0 , 3.5, 3.2 , 3.2, 2.9))  +
  labs(x = "Stimulus Content",
       y = "Duration of first Fixation (FFD)",
       fill = " ") +
  theme(axis.text.x = element_text(size = 10))+
  guides(fill = FALSE)+
  theme_tq()

fig_DFF
```

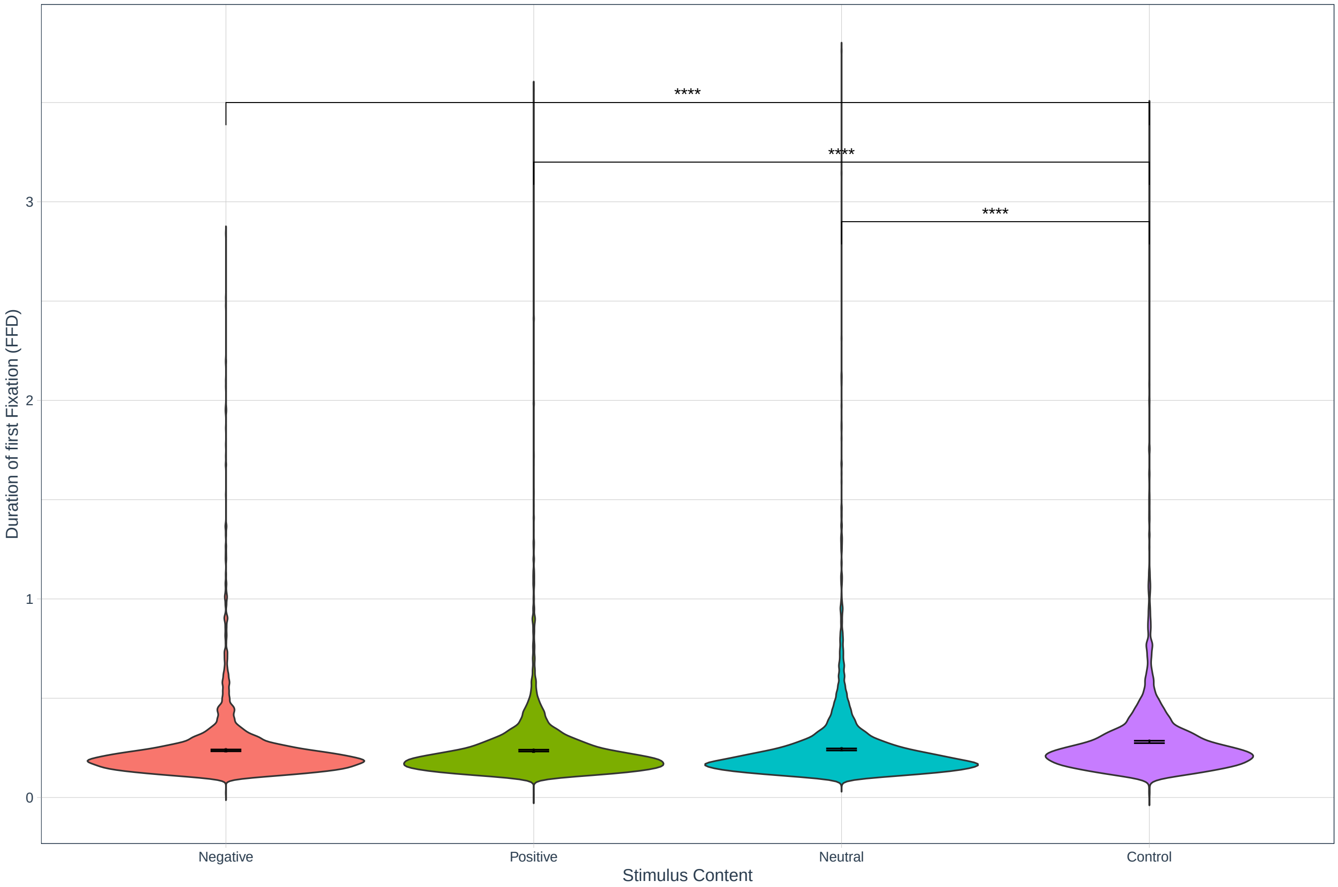

Figure S2. *Dimension between the emotional content of the stimulus, images with negative, positive, neutral and control emotional content, see Table S15, where the contrasts between each dimension are observed. In all cases, significant effects are represented with lines and stars: *p < .05, **p < .01, ***p < .001, ****p < .0001.*

2.2.2.3 Bootstrap Given that the model is a generalized linear mixed model with a non-normal error distribution (Gamma with log link), standard inferential techniques based on asymptotic normality may not provide reliable estimates. Therefore, a non-parametric bootstrap approach was used to better estimate the uncertainty associated with the fixed effects.

The bootstrap method involves generating multiple resamples from the original dataset (with replace-

ment) and fitting the same model on each sample. This process produces an empirical sampling distribution of the statistic of interest, in this case, the Duration of first Fixation (FFD).

```
boot_fn <- function(data, indices) {
  d <- data[indices, ]
  fit <- glmmTMB(First_fix_duration ~ Stimulus_content * Gender +
                  (1 + Stimulus_content | Participant) +
                  (1 | Stimulus),
                 data = d, family = Gamma(link = "log"))
  return(fixef(fit)$cond)
}

set.seed(824)
mod2_boot <- boot(data = bd1, statistic = boot_fn, R = 1000)

boot.ci <- boot.ci(mod2_boot, type = "basic", index = 1)
terms <- names(fixef(mod2_glmm)$cond)
boots_mod2_df <- data.frame(
  term = terms,
  observed = fixef(mod2_glmm)$cond,
  rep.mean = colMeans(mod2_boot$t),
  se = apply(mod2_boot$t, 2, sd),
  bias = colMeans(mod2_boot$t) - fixef(mod2_glmm)$cond
)

get_ci <- function(boot_obj, index) {
  ci <- boot.ci(boot_obj, type = "basic", index = index)
  if (!is.null(ci$basic)) {
    return(c(lower = ci$basic[4], upper = ci$basic[5]))
  } else {
    return(c(lower = NA, upper = NA))
  }
}


boot_cis <- t(sapply(1:length(terms), function(i) get_ci(mod2_boot, i)))
boots_mod2_df$ci.lower <- boot_cis[, "lower"]
boots_mod2_df$ci.upper <- boot_cis[, "upper"]


boots_mod2_df <- boots_mod2_df |>
  mutate(term = str_replace_all(term, ":", " × ")) |>
  mutate(term = str_replace_all(term, "Stimulus_contentControl", "Stimulus content [Control]")) |>
  mutate(term = str_replace_all(term, "Stimulus_contentPositive", "Stimulus content [Positive]")) |>
  mutate(term = str_replace_all(term, "Stimulus_contentNeutral", "Stimulus content [Neutral]")) |>
  mutate(term = str_replace_all(term, "GenderMale", "Gender [Male]"))

orden_deseado <- c("(Intercept)",
                   "Stimulus content [Positive]",
                   "Stimulus content [Neutral]",
                   "Stimulus content [Control]" ,
                   "Gender [Male]",
                   "Stimulus content [Positive] × Gender [Male]",
                   "Stimulus content [Neutral] × Gender [Male]",
                   "Stimulus content [Control] × Gender [Male]")

boots_mod2_df <- boots_mod2_df |> arrange(match(term, orden_deseado))
```

```
rownames(boots_mod2_df) <- NULL

boots_mod2_df |>
  select(term, observed, rep.mean, se, bias, ci.lower, ci.upper) |>
  rename(
    Effect = term,
    Estimate = observed,
    `Boot Mean` = rep.mean,
    `Std. Error` = se,
    Bias = bias,
    `CI Lower` = ci.lower,
    `CI Upper` = ci.upper
  ) |>
  kable(digits = 4, booktabs = TRUE,
        caption = "Bootstrap estimates of model 2",
        align = "lcccccc",
         rownames = FALSE) |>
  kable_styling(latex_options = c("HOLD_position"))
```

Table S16. *Bootstrap estimates of model 2*

Effect	Estimate	Boot Mean	Std. Error	Bias	CI Lower	CI Upper
(Intercept)	-1.4177	-1.4247	0.0155	-7e-03	-1.4417	-1.3797
Stimulus content [Positive]	-0.0118	-0.0110	0.0204	8e-04	-0.0533	0.0275
Stimulus content [Neutral]	0.0310	0.0310	0.0223	0e+00	-0.0111	0.0728
Stimulus content [Control]	0.1542	0.1543	0.0211	1e-04	0.1129	0.1940
Gender [Male]	-0.0410	-0.0402	0.0202	8e-04	-0.0808	-0.0003
Stimulus content [Positive] × Gender [Male]	0.0144	0.0154	0.0271	1e-03	-0.0401	0.0666
Stimulus content [Neutral] × Gender [Male]	-0.0240	-0.0241	0.0284	-1e-04	-0.0805	0.0316
Stimulus content [Control] × Gender [Male]	0.0210	0.0219	0.0284	9e-04	-0.0333	0.0768

2.2.3 First fixation count

This variable captures an early attentional measure, based on how frequently each type of stimulus (positive, negative, neutral, control) was the first to be fixated by participants during the initial visual competition. Since each trial presented four competing images, we recorded which type of image was viewed first and then calculated the total number of first fixations for each stimulus type.

2.2.3.1 Fit model To analyze first fixation counts, we fitted a generalized linear mixed-effects model (GLMM) with a Conway–Maxwell–Poisson (COM-Poisson) distribution, which is appropriate for count data that show under-dispersion. The model included Stimulus_content and Gender as fixed effects, along with their interaction, and a random intercept for Participant to account for individual differences in fixation tendencies.

```
mod3 <- glmmTMB(
  n ~ First_fixation * Gender +
    (1 | Participant),
  family = compois(),
  data = pf
)
```

2.2.3.1.1 Model assumptions Simulation-based residual diagnostics were conducted using the DHARMa package to evaluate model fit. The dispersion test indicated no over- or under-dispersion beyond that modeled by the COM-Poisson distribution, and the zero-inflation test indicated no excess zeros.

```
res <- simulateResiduals(mod3)
plot(res)
```

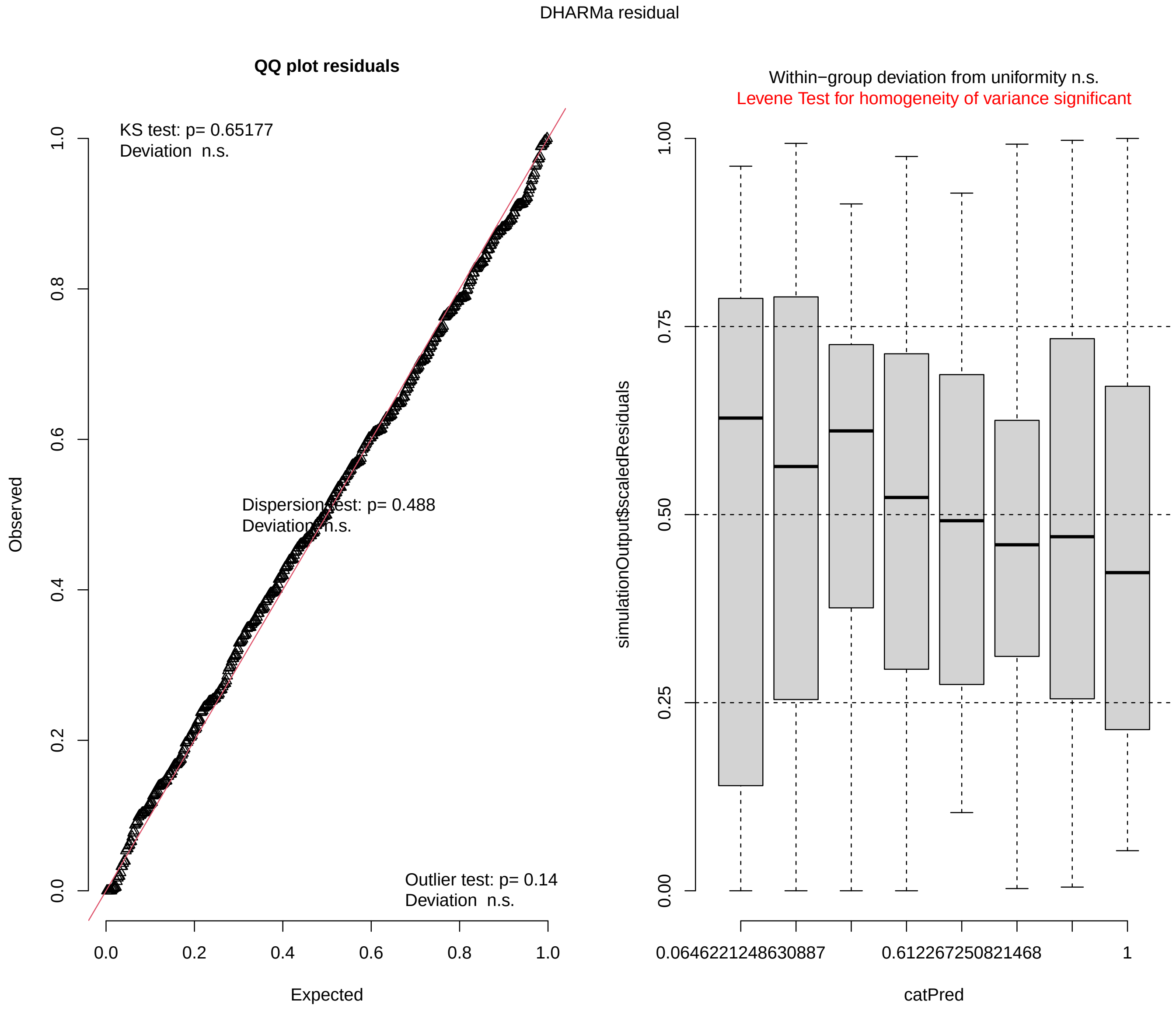


```
testDispersion <- testDispersion(res)
```

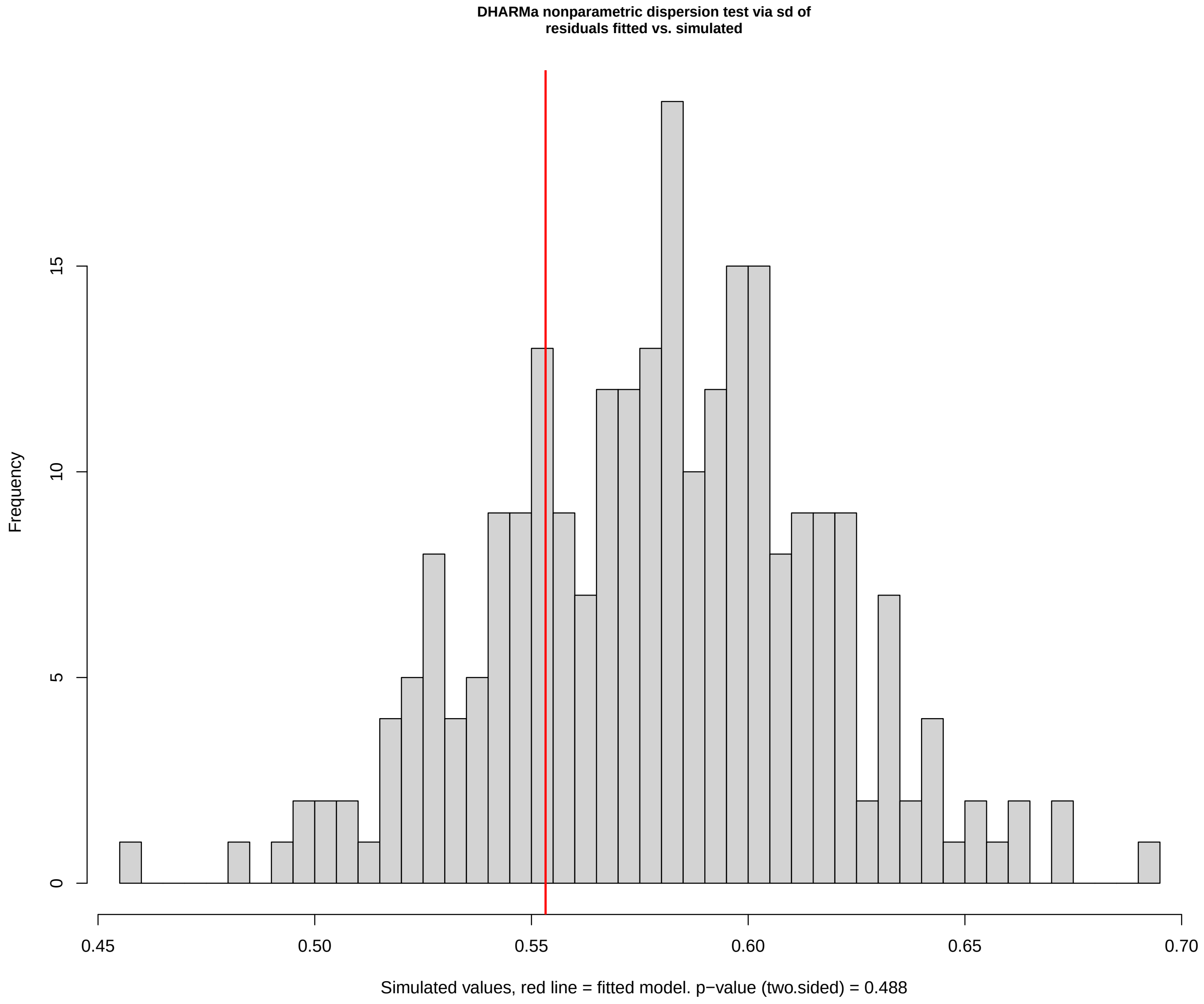

2.2.3.1.2 Summary model To summarize the fixed and random effects of the GLMM, we used the summary() function from glmmTMB, which provides coefficient estimates, standard errors, Wald z-statistics, and associated p-values for each predictor. To evaluate the significance of the main effects and their interaction, we computed a Type III Wald chi-square test using the Anova() function from the car package. This test reports chi-square statistics and degrees of freedom for each term.

```
anova_com <- Anova(mod3, type = 3)

anova_com_df <- as.data.frame(anova_com) |>
  rownames_to_column() |>
  mutate(Effect = str_replace_all(rowname, ":", " × "),
         Effect = str_replace_all(Effect, "First_fixation", "Stimulus content"),
         Effect = str_replace_all(Effect, "Gender", "Participant gender")) |>
  select(Effect, Chisq, Df, `Pr(>Chisq)`) |>
  kable(digits = 3, booktabs = TRUE,
        caption = "Effects of stimulus content and participant gender on First Fixation Count (FFC), COM-P
  kable_styling(latex_options = c("hold_position"))

anova_com_df
```

Table S17. *Effects of stimulus content and participant gender on First Fixation Count (FFC), COM-Poisson GLMM (Type III Wald test)*

Effect	Chisq	Df	Pr(>Chisq)
(Intercept)	6043.412	1	0.000
Stimulus content	158.857	3	0.000
Participant gender	2.131	1	0.144
Stimulus content × Participant gender	9.027	3	0.029

```
sum_com <- summary(mod3)


sum_com_df <- as.data.frame(sum_com$coefficients$cond) |>
  rownames_to_column(var = "Effect") |>
  mutate(Effect = str_replace_all(Effect, "First_fixation", "Stimulus content"),
         Effect = str_replace_all(Effect, "GenderMale", "Participant gender [Male]"),
         Effect = str_replace_all(Effect, ":", " × ")) |>
  kable(digits = 3, booktabs = TRUE,
        caption = "Parameter estimates for the COM-Poisson GLMM predicting First Fixation Count (FFC)") |>
  kable_styling(latex_options = c("HOLD_position"))

sum_com_df
```

Table S18. *Parameter estimates for the COM-Poisson GLMM predicting First Fixation Count (FFC)*

Effect	Estimate	Std. Error	z value	Pr(>|z|)
(Intercept)	2.304	0.030	77.739	0.000
Stimulus contentPositive	-0.174	0.044	-3.971	0.000
Stimulus contentNeutral	-0.193	0.044	-4.371	0.000
Stimulus contentControl	-0.638	0.051	-12.557	0.000
Participant gender [Male]	0.059	0.040	1.460	0.144
Stimulus contentPositive × Participant gender [Male]	-0.159	0.061	-2.608	0.009
Stimulus contentNeutral × Participant gender [Male]	-0.085	0.061	-1.396	0.163
Stimulus contentControl × Participant gender [Male]	0.016	0.069	0.228	0.820

```
r2mod3 <- r2_nakagawa(mod3)
```

```
## Random effect variances not available. Returned R2 does not account for random effects.
```

```
r2_tblmod3 <- as_tibble(r2mod3)
kable(r2_tblmod3, format = "markdown",  booktabs =TRUE,
      align = "c", caption = "R model 3",
      escape = FALSE) %>%
      kable_styling(latex_options = c("HOLD_position"))
```

Table S19. *R model 3*

R2_conditional	R2_marginal
	0.0947283

```
sing3 <- performance::check_singularity(mod3)

sd_intercept <- try({
```

```
  vc3_cond <- VarCorr(mod3)$cond
  vc3_df   <- as.data.frame(vc3_cond)  # aquí sí se puede
  # Detectar nombres de columnas (cambian entre versiones)
  col_var1 <- intersect(c("var1","Var1","term","Term"), names(vc3_df))[1]
  col_sd   <- intersect(c("sdcor","SD","sd","Std.Dev","StdDev","estimate"), names(vc3_df))[1]

  # Fila del intercepto (sin var2 => no correlación)
  if (!"var2" %in% names(vc3_df)) vc3_df$var2 <- NA_character_
  vc3_df %>%
    filter(is.na(var2), .data[[col_var1]] == "(Intercept)") %>%
    pull(!!col_sd) %>%
    as.numeric() %>%
    `[`(1)
}, silent = TRUE)


if (inherits(sd_intercept, "try-error") || is.na(sd_intercept)) {
  td <- broom.mixed::tidy(mod3, effects = "ran_pars", component = "cond")
  # term típico: "sd__(Intercept)"; columna estimate trae el SD
  sd_intercept <- td %>%
    filter(grepl("^sd__\\(Intercept\\)$", term) | grepl("\\(Intercept\\)", term)) %>%
    pull(estimate) %>%
    as.numeric() %>%
    `[`(1)
}

tab_sing3 <- tibble::tibble(
  Check  = c("Model singularity", "Random effect (Group)", "Effect", "SD"),
  Result = c(
    ifelse(sing3, "TRUE (Model is singular)", "FALSE (Model is not singular)"),
    "Participant",
    "Intercept",
    ifelse(is.finite(sd_intercept), sprintf("%.6f", sd_intercept), "NA")
  )
)

kable(tab_sing3, booktabs = TRUE, align = c("l","c"),
      caption = "Singularity check and random-effect (Intercept) - Model 3") %>%
  kable_styling(latex_options = c("HOLD_position"))
```

Table S20. *Singularity check and random-effect (Intercept) — Model 3*

Check	Result
Model singularity	TRUE (Model is singular)
Random effect (Group)	Participant
Effect	Intercept
SD	0.000006

2.2.3.1.3 Estimated marginal means Given the significant interaction between stimulus content and participant gender, estimated marginal means (EMMs) were computed and pairwise contrasts were performed to examine how first fixation probabilities differed across stimulus types within each gender. Post hoc comparisons were adjusted using the Tukey method to control for multiple testing. The results below present the contrasts separately for male and female participants, allowing a clearer interpretation of the interaction effect.

```
emm_mod3   <- emmeans(mod3, ~ First_fixation | Gender, type = "response")
contr_mod3 <- contrast(emm_mod3, "pairwise", adjust = "tukey")
contr_df <- as.data.frame(contr_mod3)

est_col  <- if ("estimate" %in% names(contr_df)) "estimate" else if ("ratio" %in% names(contr_df)) "ratio"
stat_col <- if ("t.ratio" %in% names(contr_df)) "t.ratio" else "z.ratio"


format_contrast <- function(contrast, gender) {
  m <- stringr::str_match(contrast, "^\\s*([^/\\-]+)\\s*[/-]\\s*([^/\\-]+)\\s*$")
  lhs <- trimws(m[,2]); rhs <- trimws(m[,3])
  paste(lhs, gender, "-", rhs, gender)
}


contr_fmt <- contr_df %>%
  mutate(
    contrast_fmt = format_contrast(contrast, Gender),
    estimate     = .data[[est_col]],
    stat         = .data[[stat_col]],
    SE           = if ("SE" %in% names(.)) SE else NA_real_
  ) %>%
  transmute(
    Gender,
    contrast = contrast_fmt,
    estimate, SE,
    `t.ratio` = stat,
    p.value
  )

contrasts_male_only <- contr_fmt %>% filter(Gender == "Male") %>% select(-Gender)

kable(contrasts_male_only, digits = 5, booktabs = TRUE, align = "c",
      caption = "Contrasts between stimulus content for Male - Model 3") %>%
  kable_styling(latex_options = "HOLD_position", font_size = 12, full_width = FALSE)
```

Table S21. *Contrasts between stimulus content for Male - Model 3*

contrast	estimate	SE	t.ratio	p.value
Negative Male - Positive Male	1.39630	0.05935	7.85442	0.00000
Negative Male - Neutral Male	1.32039	0.05519	6.64920	0.00000
Negative Male - Control Male	1.86301	0.08691	13.33814	0.00000
Positive Male - Neutral Male	0.94563	0.04286	-1.23338	0.60554
Positive Male - Control Male	1.33425	0.06649	5.78675	0.00000
Neutral Male - Control Male	1.41096	0.06947	6.99259	0.00000

```
contrasts_female_only <- contr_fmt %>% filter(Gender == "Female") %>% select(-Gender)

kable(contrasts_female_only, digits = 5, booktabs = TRUE, align = "c",
      caption = "Contrasts between stimulus content for Female - Model 3") %>%
  kable_styling(latex_options = "HOLD_position", font_size = 12, full_width = FALSE)
```

Table S22. *Contrasts between stimulus content for Female - Model 3*

contrast	estimate	SE	t.ratio	p.value
Negative Female - Positive Female	1.19057	0.05230	3.97077	0.00042
Negative Female - Neutral Female	1.21294	0.05357	4.37149	0.00007
Negative Female - Control Female	1.89251	0.09614	12.55712	0.00000
Positive Female - Neutral Female	1.01879	0.04694	0.40402	0.97771
Positive Female - Control Female	1.58958	0.08341	8.83276	0.00000
Neutral Female - Control Female	1.56026	0.08217	8.44680	0.00000

2.2.3.2 Figure Taking into account the results of the COM-Poisson GLMM—showing a significant effect of stimulus content and a stimulus content*gender interaction—post hoc Tukey-adjusted pairwise comparisons were conducted within each gender. Results are presented in separate panels for women and men to facilitate interpretation of the interaction.

```
emm_raw <- as.data.frame(emm_mod3)
FFC <- if ("response" %in% names(emm_raw)) emm_raw$response else emm_raw$emmean
LCL <- if ("asymp.LCL" %in% names(emm_raw)) emm_raw$asymp.LCL else emm_raw$lower.CL
UCL <- if ("asymp.UCL" %in% names(emm_raw)) emm_raw$asymp.UCL else emm_raw$upper.CL

emm_df <- tibble(
  Gender           = emm_raw$Gender,
  Stimulus_content = emm_raw$First_fixation,
  FFC              = FFC,
  SE               = emm_raw$SE,
  LCL              = LCL,
  UCL              = UCL
)

contr_df <- as.data.frame(contr_mod3)
est_col  <- if ("estimate" %in% names(contr_df)) "estimate" else if ("ratio" %in% names(contr_df)) "ratio"
stat_col <- if ("t.ratio" %in% names(contr_df)) "t.ratio" else "z.ratio"

format_contrast <- function(contrast, gender){
  m <- stringr::str_match(contrast, "^\\s*([^/\\-]+)\\s*[/-]\\s*([^/\\-]+)\\s*$")
  paste0(trimws(m[,2]), " ", gender, " - ", trimws(m[,3]), " ", gender)
}

contr_fmt <- contr_df %>%
  mutate(
    contrast_lbl = format_contrast(contrast, Gender),
    estimate     = .data[[est_col]],
    stat         = .data[[stat_col]]
  ) %>%
  transmute(Gender, contrast = contrast_lbl, estimate, SE = if ("SE" %in% names(contr_df)) SE else NA_real_

contrasts_male <- contr_fmt %>% filter(Gender == "Male") %>%
  filter(contrast %in% c(
    "Neutral Male - Control Male",
    "Positive Male - Control Male",
    "Positive Male - Neutral Male",
    "Negative Male - Control Male",
    "Negative Male - Neutral Male",
    "Negative Male - Positive Male"
```

```
  )) %>%
  mutate(p.signif = pval.stars(p.value)) %>%
  separate_wider_delim(contrast, " - ", names = c("group1", "group2")) %>%
  select(group1, group2, t.ratio, p.value, p.signif)

contrasts_female <- contr_fmt %>% filter(Gender == "Female") %>%
  filter(contrast %in% c(
    "Negative Female - Positive Female",
    "Negative Female - Neutral Female",
    "Negative Female - Control Female",
    "Positive Female - Neutral Female",
    "Positive Female - Control Female",
    "Neutral Female - Control Female"
  )) %>%
  mutate(p.signif = pval.stars(p.value)) %>%
  separate_wider_delim(contrast, " - ", names = c("group1", "group2")) %>%
  select(group1, group2, t.ratio, p.value, p.signif)

pf_hom <- pf %>%
  filter(Gender == "Male") %>%
  rename(Stimulus_content = First_fixation, First_Fixation_count = n)

pf_muj <- pf %>%
  filter(Gender == "Female") %>%
  rename(Stimulus_content = First_fixation, First_Fixation_count = n)

emm_hom <- emm_df %>% filter(Gender == "Male") %>% select(-Gender) %>%
  rename(First_Fixation_count = FFC)
emm_muj <- emm_df %>% filter(Gender == "Female") %>% select(-Gender) %>%
  rename(First_Fixation_count = FFC)


y_max_male   <- max(pf_hom$First_Fixation_count, na.rm = TRUE)
y_max_female <- max(pf_muj$First_Fixation_count, na.rm = TRUE)

ypos_male   <- c(y_max_male+1, y_max_male+3, y_max_male+5, y_max_male+2, y_max_male+4, y_max_male+6)
ypos_female <- c(y_max_female+1, y_max_female+3, y_max_female+5, y_max_female+2, y_max_female+4, y_max_fema

tbl_male <- contrasts_male %>% mutate(y.position = ypos_male, group1 = str_replace(group1, " Male", ""), g
tbl_female <- contrasts_female %>% mutate(y.position = ypos_female, group1 = str_replace(group1, " Female"

fig_ffc_male <- ggplot(pf_hom, aes(x = Stimulus_content, y = First_Fixation_count)) +
  geom_violin(trim = FALSE, aes(fill = Stimulus_content)) +
  geom_jitter(alpha = 0.05, width = 0.25) +
  geom_errorbar(data = emm_hom, aes(ymin = First_Fixation_count - SE, ymax = First_Fixation_count + SE), w
  geom_point(data = emm_hom, shape = 21, size = 2.2, color = "black", fill = "white") +
  stat_pvalue_manual(tbl_male,
                     label = "p.signif",
                    y.position = c(25, 29, 31, 27, 27, 25) ) +
  labs(title = "Men", x = "Stimulus content", y = "First Fixation Count (FFC)") +
  guides(fill = "none") +
  theme_minimal() +
  theme(axis.text.x = element_text(size = 10))

fig_ffc_female <- ggplot(pf_muj, aes(x = Stimulus_content, y = First_Fixation_count)) +
  geom_violin(trim = FALSE, aes(fill = Stimulus_content)) +
```

```
  geom_jitter(alpha = 0.05, width = 0.25) +
  geom_errorbar(data = emm_muj, aes(ymin = First_Fixation_count - SE, ymax = First_Fixation_count + SE), w
  geom_point(data = emm_muj, shape = 21, size = 2.2, color = "black", fill = "white") +
  stat_pvalue_manual(tbl_female,
                     label = "p.signif",
                    y.position = c(25, 29, 31, 27, 27, 25)) +
  labs(title = "Women", x = "Stimulus content", y = "First Fixation Count (FFC)") +
  guides(fill = "none") +
  theme_minimal() +
  theme(axis.text.x = element_text(size = 10))


figura_ffc <- ggarrange(fig_ffc_female, fig_ffc_male, nrow = 1, labels = "auto")
figura_ffc
```

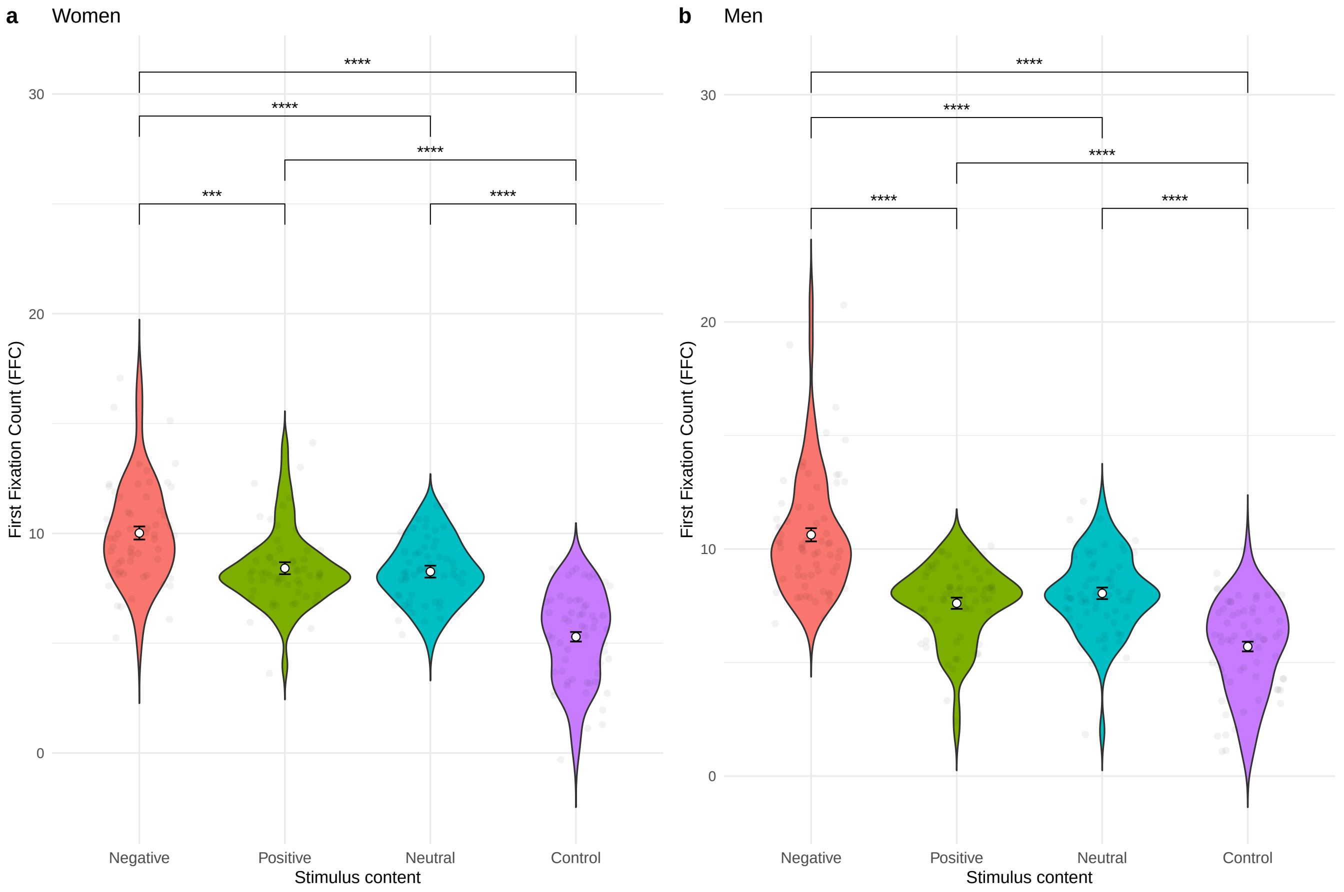

Figure S3. *Dimension between the emotional content of the stimulus, images with negative, positive, neutral and control emotional content, by gender of the participants, see Table S21 and Table S22, where the contrasts between each dimension are observed. In all cases, significant effects are represented with lines and stars: *p < .05, **p < .01, ***p < .001, ****p < .0001.*

2.2.3.3 Bootstrap Considering that Model 3 was fitted using a Generalized Linear Mixed Model (GLMM) with a Conway–Maxwell–Poisson (COM-Poisson) distribution, a bootstrap procedure was implemented to obtain robust estimates of parameter variability and confidence intervals. The bootstrap method was used to generate multiple resampled datasets from the original data and to estimate the empirical sampling distribution of each model coefficient.

```
template_names <- names(fixef(mod3)$cond)
```

```
boot_fun <- function(data, indices) {
  d <- data[indices, ]
  fit <- try(
    glmmTMB(
      n ~ First_fixation * Gender + (1 | Participant),
      family = compois(),
      data = d
    ),
    silent = TRUE
  )
  if (inherits(fit, "try-error")) {
    return(rep(NA_real_, length(fixef(mod3)$cond)))
  } else {
    return(fixef(fit)$cond)
  }
}

R <- 1000
boot_obj <- boot(data = pf, statistic = boot_fun, R = R)

boot_mat <- as.matrix(boot_obj$t)
colnames(boot_mat) <- template_names

observed <- fixef(mod3)$cond[template_names]


rep.mean <- apply(boot_mat, 2, mean,    na.rm = TRUE)
lower    <- apply(boot_mat, 2, quantile, probs = 0.025, na.rm = TRUE)
upper    <- apply(boot_mat, 2, quantile, probs = 0.975, na.rm = TRUE)
bias     <- rep.mean - observed

label_term <- function(x) {
  x <- gsub("\\(Intercept\\)", "(Intercept)", x)
  x <- gsub("First_fixationPositive", "Stimulus content [Positive]", x)
  x <- gsub("First_fixationNeutral",  "Stimulus content [Neutral]",  x)
  x <- gsub("First_fixationControl",  "Stimulus content [Control]",  x)
  x <- gsub("GenderMale",             "Gender [Male]",               x)
  x <- gsub(":", " × ", x)
  x
}

res_tbl <- tibble(
  term     = label_term(names(observed)),
  observed = as.numeric(observed),
  rep.mean = as.numeric(rep.mean),
  bias     = as.numeric(bias),
  lower    = as.numeric(lower),
  upper    = as.numeric(upper)
)


orden_deseado <- c(
  "Stimulus content [Positive]",
  "Stimulus content [Neutral]",
  "Stimulus content [Control]",
  "Gender [Male]",
```

```
  "Stimulus content [Control] × Gender [Male]",
  "Stimulus content [Neutral] × Gender [Male]",
  "Stimulus content [Positive] × Gender [Male]"
)


res_tbl <- res_tbl %>%
  filter(term %in% orden_deseado) %>%
  mutate(order_key = match(term, orden_deseado)) %>%
  arrange(order_key) %>%
  select(-order_key)

kable(
  res_tbl,
  digits = 4,
  booktabs = TRUE,
  caption = "Bootstrap estimated values of Model 3 (COM-Poisson GLMM)"
) %>%
  kable_styling(latex_options = c("HOLD_position"))
```

Table S23. *Bootstrap estimated values of Model 3 (COM-Poisson GLMM)*

term	observed	rep.mean	bias	lower	upper
Stimulus content [Positive]	-0.1744	-0.1728	0.0016	-0.2507	-0.0947
Stimulus content [Neutral]	-0.1931	-0.1923	0.0008	-0.2649	-0.1201
Stimulus content [Control]	-0.6379	-0.6399	-0.0020	-0.7553	-0.5280
Gender [Male]	0.0589	0.0592	0.0003	-0.0273	0.1466
Stimulus content [Control] × Gender [Male]	0.0157	0.0198	0.0041	-0.1530	0.1813
Stimulus content [Neutral] × Gender [Male]	-0.0849	-0.0849	0.0000	-0.1999	0.0285
Stimulus content [Positive] × Gender [Male]	-0.1594	-0.1612	-0.0019	-0.2834	-0.0471

2.2.4 Total Duration of Fixation

The Total Duration of Fixation is a measure of delayed attention, which consists of the total time that the participant observes a stimulus. It should be noted that it is the cumulative time of each time the participant decides to view the image.

2.2.4.1 Fit model In the fourth model, Total Duration of Fixation is predicted by the stimulus type and its interaction with the participant's gender. A random intercept was included for the participant to account for individual variability in attention patterns. Initially, a random intercept for the stimulus was included.

The model assumes a Gamma distribution with a log link, appropriate for modeling positively skewed duration data.

```
mod4_glmm <- glmmTMB(
  Total_duration ~ Stimulus_content * Gender +
    (1 + Stimulus_content || Participant) +
    (1 | Stimulus),
  family = Gamma(link = "log"),
  data = bd
)
```

2.2.4.1.1 Model assumptions The following functions 'check_model()', 'check_distribution()', and 'check_normality()' are used to evaluate the quality of fit and distribution of residuals in a statistical model. For example, check_model() could provide a visualization of residuals and diagnose

problems such as heteroscedasticity or bias in the model. check_distribution() could provide a visualization of the distribution of residuals and diagnose problems such as deviation from normality or outliers.

```r
check_model(mod4_glmm)
```

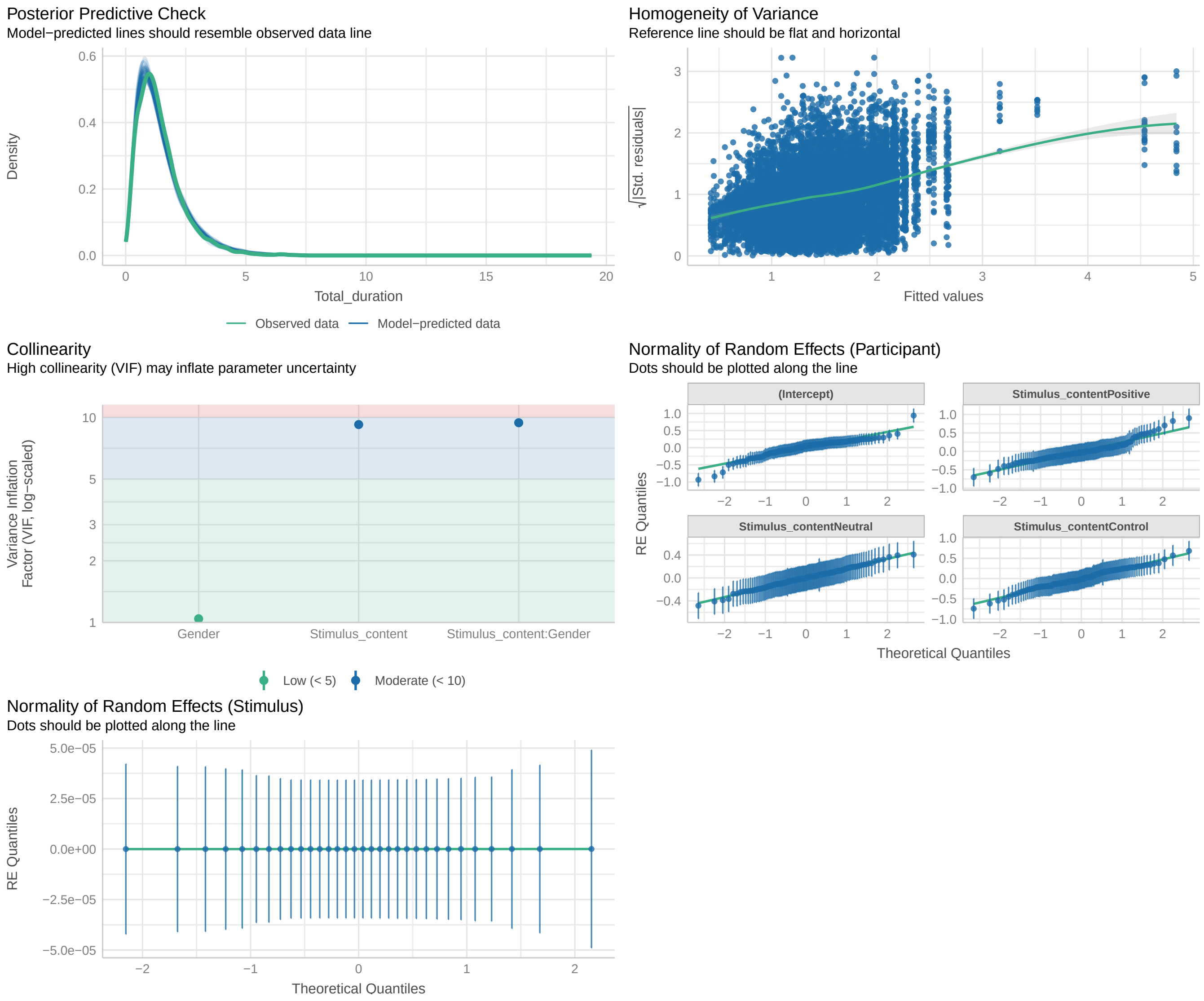


```r
dist_checkmod4 <- check_distribution(mod4_glmm)
kable(data.frame(dist_checkmod4),
      digits = 2, booktabs = TRUE,
      caption = "Distribution of residuals model 4") %>%
      kable_styling(latex_options = c("HOLD_position"))
```

Table S24. *Distribution of residuals model 4*

Distribution	p_Residuals	p_Response
bernoulli	0.00	0.00
beta	0.00	0.03
beta-binomial	0.00	0.03
binomial	0.00	0.00
cauchy	0.47	0.00
chi	0.03	0.00
exponential	0.22	0.03
F	0.00	0.22
gamma	0.09	0.34
half-cauchy	0.00	0.00
inverse-gamma	0.00	0.00
lognormal	0.00	0.06
neg. binomial (zero-infl.)	0.00	0.00
negative binomial	0.00	0.00
normal	0.12	0.00
pareto	0.00	0.00
poisson	0.00	0.00
poisson (zero-infl.)	0.00	0.00
tweedie	0.03	0.28
uniform	0.00	0.00
weibull	0.03	0.00

2.2.4.1.2 Summary model To examine the results of model 4, we used three complementary summaries. First, we used the Anova() function from the car package to obtain a Type III Wald chi-square test, which evaluates the significance of each predictor and their interaction in the model. This method is appropriate for generalized linear mixed models fitted with glmmTMB.

Second, we applied the summary() function to obtain the estimated coefficients, their standard errors, z-values, and associated p-values for the fixed effects.

Third, we calculated the marginal and conditional pseudo R^2 values using the r2_nakagawa() function. These values reflect the proportion of variance explained by the fixed effects alone (marginal R^2) and by both fixed and random effects combined (conditional R^2), following the method proposed by Nakagawa and Schielzeth (Nakagawa & Schielzeth, 2013).

```
anova.mod4 <- Anova(mod4_glmm, type = 3)
anova.mod4 <- as.data.frame(anova.mod4)|>
  rownames_to_column() |>
  mutate_at("rowname", str_replace_all, ":", " × ") |>
  mutate_at("rowname", str_replace_all, "Stimulus_content",
          "Stimulus content")|>
  rename( Effect = rowname)|>
  kable(digits = 2, booktabs = TRUE,
        align = c("l", "l", rep("c", 4)), caption = "Effect of Stimulus
        Content and Participant's Gender
        on Total Duration of Fixations (TDF)",
        escape = FALSE)|>
        kable_styling(latex_options = c("hold_position"))
anova.mod4
```

```
sum.mod4 <- summary(mod4_glmm)
```

Table S25. *Effect of Stimulus Content and Participant's Gender on Total Duration of Fixations (TDF)*

Effect	Chisq	Df	Pr(>Chisq)
(Intercept)	213.24	1	0.00
Stimulus content	200.24	3	0.00
Gender	1.01	1	0.32
Stimulus content × Gender	0.84	3	0.84

```
sum.mod4_df <- tidy(mod4_glmm, effects = "fixed") |>
  mutate(term = str_replace_all(term, ":", " × ")) |>
  mutate(term = str_replace_all(term, "Stimulus_contentControl",
                                "Stimulus content [Control]")) |>
  mutate(term = str_replace_all(term, "Stimulus_contentPositive",
                                "Stimulus content [Positive]")) |>
  mutate(term = str_replace_all(term, "Stimulus_contentNeutral",
                                "Stimulus content [Neutral]")) |>
  mutate(term = str_replace_all(term, "GenderMale",
                                "Gender [Male]")) |>
  rename (Effect = term,
         Estimate = estimate,
         `Std. Error` = std.error,
         `z value` = statistic,
         `Pr(>|z|)` = p.value)|>
  select(Effect, Estimate, `Std. Error`, `z value`, `Pr(>|z|)`)

sum.mod4_df |>
  kable(digits = 2, booktabs = TRUE,
        align = c("l", rep("c", 5)),
        caption = "Total Duration of Fixations (TDF)
          by stimylus content,
        gender and the interaction
        between stimulus contentand gender",
        escape = FALSE) |>
  kable_styling(latex_options = c("HOLD_position"))
```

Table S26. *Total Duration of Fixations (TDF) by stimylus content, gender and the interaction between stimulus contentand gender*

Effect	Estimate	Std. Error	z value	Pr(>|z|)
(Intercept)	0.52	0.04	14.60	0.00
Stimulus content [Positive]	-0.16	0.04	-3.96	0.00
Stimulus content [Neutral]	-0.29	0.03	-9.18	0.00
Stimulus content [Control]	-0.46	0.04	-11.77	0.00
Gender [Male]	0.05	0.05	1.00	0.32
Stimulus content [Positive] × Gender [Male]	-0.02	0.06	-0.33	0.74
Stimulus content [Neutral] × Gender [Male]	-0.01	0.04	-0.26	0.79
Stimulus content [Control] × Gender [Male]	-0.05	0.05	-0.88	0.38

```
r2mod4 <- r2_nakagawa(mod4_glmm)
```

```
## Random effect variances not available. Returned R2 does not account for random effects.
```

```
r2_tblmod4 <- as_tibble(r2mod4)
kable(r2_tblmod4, format = "markdown",  booktabs =TRUE,
```

```
      align = "c", caption = "R model 4",
      escape = FALSE) %>%
      kable_styling(latex_options = c("HOLD_position"))
```

Table S27. *R model 4*

R2_conditional	R2_marginal
	0.1049118

```
re_par <- parameters::model_parameters(mod4_glmm, effects = "random")
re_df  <- as.data.frame(re_par)

col_param <- intersect(c("Parameter","Term","Name"), names(re_df))[1]
col_est   <- intersect(c("SD","SD (random effect)","Coefficient","Estimate","Est."), names(re_df))[1]
col_group <- intersect(c("Group","Cluster"), names(re_df))[1]

tab_sd <- re_df %>%
  transmute(
    Group  = .data[[col_group]],
    Effect_raw = .data[[col_param]],
    SD   = suppressWarnings(as.numeric(.data[[col_est]]))
  ) %>%
  filter(grepl("^SD\\s*\\(", Effect_raw)) %>%
  mutate(
    Effect = str_replace(Effect_raw, "^SD\\s*\\((.*)\\)$", "\\1"),
    Effect = str_replace_all(Effect, "Stimulus_content", "Stimulus content"),
    Effect = str_replace_all(Effect, "First_fixation",   "Stimulus content"),
    Effect = str_replace_all(Effect, ":", " × "),
    Effect = str_replace(Effect, "Stimulus contentPositive", "Stimulus content [Positive]"),
    Effect = str_replace(Effect, "Stimulus contentNeutral",  "Stimulus content [Neutral]"),
    Effect = str_replace(Effect, "Stimulus contentControl",  "Stimulus content [Control]"),
    Variance = SD^2
  ) %>%
  select(Group, Effect, SD, Variance)

kable(tab_sd, digits = 4, booktabs = TRUE,
      caption = "Random effects (SD and Variance) - Model 4 (Gamma GLMM)") %>%
  kable_styling(latex_options = c("HOLD_position"))
```

Table S28. *Random effects (SD and Variance) — Model 4 (Gamma GLMM)*

Group	Effect	SD	Variance
Participant	Intercept	0.2548	0.0649
Stimulus	Intercept	0.0000	0.0000
Participant	Stimulus content [Positive]	0.2801	0.0785
Participant	Stimulus content [Neutral]	0.1988	0.0395
Participant	Stimulus content [Control]	0.2643	0.0699

2.2.4.1.3 Estimated marginal means Given the significant effect observed for the stimulus content variable in the ANOVA-type table, post-hoc pairwise comparisons were performed using the emmeans() function. These contrasts allow for a detailed examination of the differences between levels of Stimulus content (i.e., Control, Neutral, Positive, Negative), adjusting for multiple comparisons and accounting for the model's link function.

The estimated marginal means were computed on the response scale using type = “response” and regridded to obtain interpretable values (i.e., in milliseconds). The table below presents the pairwise contrasts between levels of Stimulus content.

```
mcm4 <- emmeans(mod4_glmm, pairwise ~ Stimulus_content, type = "response")
mcm4$emmeans <- regrid(mcm4$emmeans)
mcm4$contrasts <- pairs(mcm4$emmeans)

mcm4_emmeans <- tibble(data.frame(mcm4$emmeans)) |>
  rename(Total_duration = response)|>
  select(-df)

contrasts_df4 <-  as.data.frame(mcm4$contrasts)|>
  select(-df)

kable(contrasts_df4, digits = 5,  booktabs =TRUE,
      align = "c", caption = "contrasts between variables, according to the
      content of the stimulus of model 4") %>%
      kable_styling(latex_options = "HOLD_position",
                    font_size = 12,
                    full_width = FALSE)
```

Table S29. *contrasts between variables, according to the content of the stimulus of model 4*

contrast	estimate	SE	z.ratio	p.value
Negative - Positive	0.27472	0.04287	6.40737	0.00000
Negative - Neutral	0.44942	0.03210	13.99867	0.00000
Negative - Control	0.66875	0.03514	19.03313	0.00000
Positive - Neutral	0.17471	0.04730	3.69352	0.00127
Positive - Control	0.39404	0.04875	8.08221	0.00000
Neutral - Control	0.21933	0.03801	5.77004	0.00000

2.2.4.2 Figure Finally, for this model 4, a figure showing the total fixation time as a function of stimulus content and the respective differences between the variables is shown.

```
tbl.contrasts_df4 <- contrasts_df4 %>%
  mutate(.y. = "Total_duration") %>%
  separate_wider_delim(contrast, " - ",
                       names = c("group1", "group2")) %>%
  select(7, 1:6) %>%
  mutate(p.signif = pval.stars(p.value))

fig_tdf <- ggplot(bd, aes(x=Stimulus_content,
               y=Total_duration)) +
  geom_violin(trim = FALSE,
              aes(fill = Stimulus_content)) +
  geom_jitter(alpha = 0.009, width = 0.3) +
  geom_errorbar(data = mcm4_emmeans,
              mapping =
                aes(ymin = Total_duration-SE,
                    ymax = Total_duration+SE),
                colour = "black", width = 0.01) +
  geom_point(data = mcm4_emmeans,
             shape = 50, size = 1,
             color = "black", fill = "white") +
```

```
  stat_pvalue_manual(tbl.contrasts_df4,
                     label = "p.signif",
                     y.position =  c(8.5, 10.5, 12.5, 9.5, 11.5, 8.5))  +
  labs(x = "Stimulus Content",
       y = "Total duration of fixations (TDF)",
       fill = " ") +
  theme(axis.text.x = element_text(size = 10))+
  guides(fill = FALSE)+
  theme_tq()

fig_tdf
```

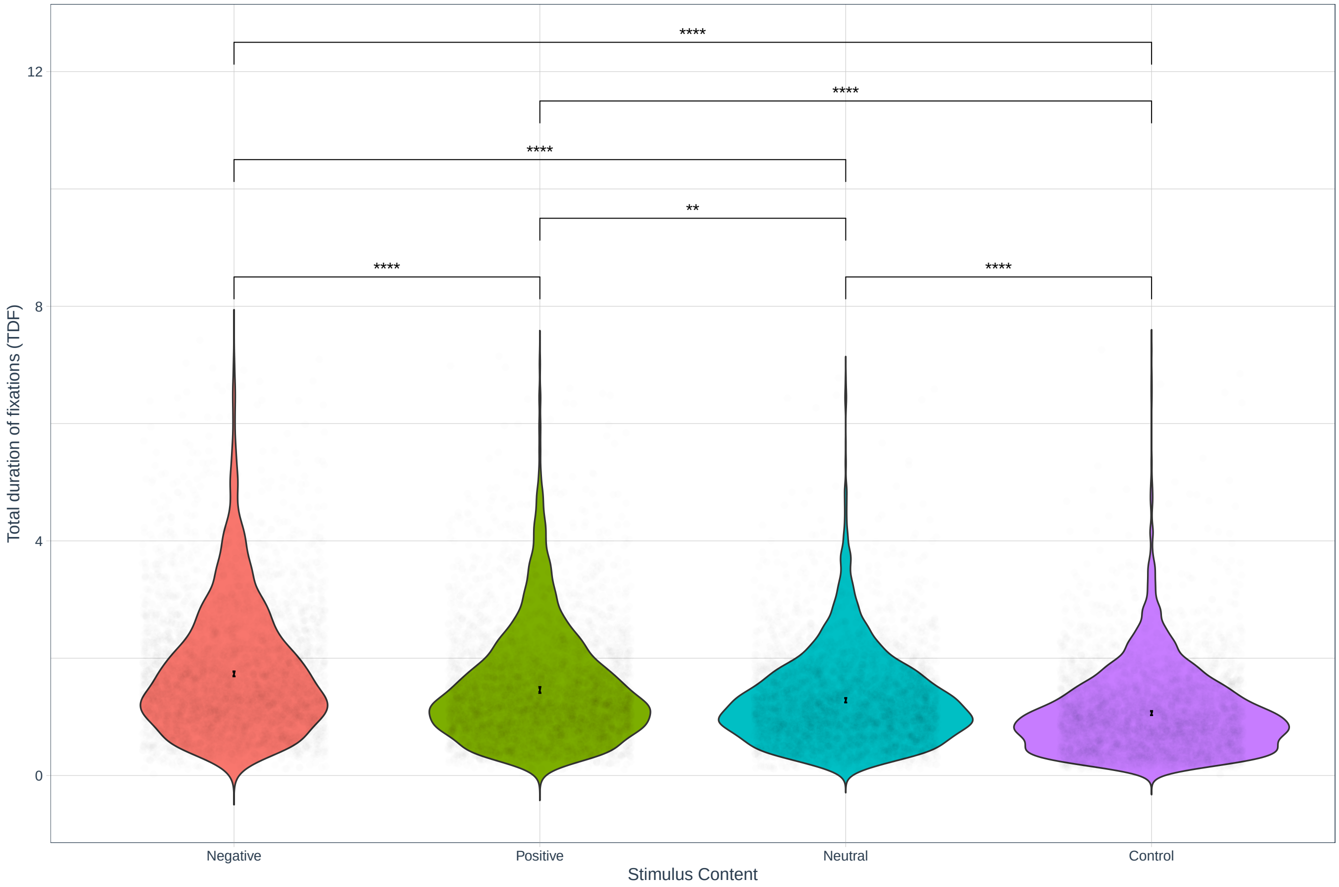

Figure S4. *Dimension between the emotional content of the stimulus, images with negative, positive, neutral and control emotional content, see Table S29 where the contrasts between each dimension are observed. In all cases, significant effects are represented with lines and stars:* *p < .05, **p < .01, ***p < .001, ****p < .0001.

2.2.4.3 Bootstrap Given that the model is a generalized linear mixed model with a non-normal error distribution (Gamma with log link), standard inferential techniques based on asymptotic normality may not provide reliable estimates. Therefore, a non-parametric bootstrap approach was used to better estimate the uncertainty associated with the fixed effects.

The bootstrap method involves generating multiple resamples from the original dataset (with replacement) and fitting the same model on each sample. This process produces an empirical sampling distribution of the statistic of interest, in this case, the Total duration of fixations (TDF).

```
boot_fn <- function(data, indices) {
  d <- data[indices, ]
  fit <- glmmTMB(Total_duration ~ Stimulus_content * Gender +
```

```
                   (1 + Stimulus_content | Participant) +
                   (1 | Stimulus),
                 data = d, family = Gamma(link = "log"))
  return(fixef(fit)$cond)
}

set.seed(824)
mod4_boot <- boot(data = bd1, statistic = boot_fn, R = 1000)

boot.ci <- boot.ci(mod4_boot, type = "basic", index = 1)
terms <- names(fixef(mod4_glmm)$cond)
boots_mod4_df <- data.frame(
  term = terms,
  observed = fixef(mod4_glmm)$cond,
  rep.mean = colMeans(mod4_boot$t),
  se = apply(mod4_boot$t, 2, sd),
  bias = colMeans(mod4_boot$t) - fixef(mod4_glmm)$cond
)

get_ci <- function(boot_obj, index) {
  ci <- boot.ci(boot_obj, type = "basic", index = index)
  if (!is.null(ci$basic)) {
    return(c(lower = ci$basic[4], upper = ci$basic[5]))
  } else {
    return(c(lower = NA, upper = NA))
  }
}


boot_cis <- t(sapply(1:length(terms), function(i) get_ci(mod4_boot, i)))
boots_mod4_df$ci.lower <- boot_cis[, "lower"]
boots_mod4_df$ci.upper <- boot_cis[, "upper"]


boots_mod4_df <- boots_mod4_df |>
  mutate(term = str_replace_all(term, ":", " × ")) |>
  mutate(term = str_replace_all(term, "Stimulus_contentControl", "Stimulus content [Control]")) |>
  mutate(term = str_replace_all(term, "Stimulus_contentPositive", "Stimulus content [Positive]")) |>
  mutate(term = str_replace_all(term, "Stimulus_contentNeutral", "Stimulus content [Neutral]")) |>
  mutate(term = str_replace_all(term, "GenderMale", "Gender [Male]"))

orden_deseado <- c("(Intercept)",
                   "Stimulus content [Positive]",
                   "Stimulus content [Neutral]",
                   "Stimulus content [Control]" ,
                   "Gender [Male]",
                   "Stimulus content [Positive] × Gender [Male]",
                   "Stimulus content [Neutral] × Gender [Male]",
                   "Stimulus content [Control] × Gender [Male]")

boots_mod4_df <- boots_mod4_df |> arrange(match(term, orden_deseado))

rownames(boots_mod4_df) <- NULL

boots_mod4_df |>
  select(term, observed, rep.mean, se, bias, ci.lower, ci.upper) |>
```

```
  rename(
    Effect = term,
    Estimate = observed,
    `Boot Mean` = rep.mean,
    `Std. Error` = se,
    Bias = bias,
    `CI Lower` = ci.lower,
    `CI Upper` = ci.upper
  ) |>
  kable(digits = 4, booktabs = TRUE,
        caption = "Bootstrap estimates of model 4",
        align = "lcccccc",
         rownames = FALSE) |>
  kable_styling(latex_options = c("HOLD_position"))
```

Table S30. *Bootstrap estimates of model 4*

Effect	Estimate	Boot Mean	Std. Error	Bias	CI Lower	CI Upper
(Intercept)	0.5249	0.5174	0.0127	-0.0075	0.5014	0.5512
Stimulus content [Positive]	-0.1631	-0.1597	0.0182	0.0034	-0.1946	-0.1249
Stimulus content [Neutral]	-0.2944	-0.2924	0.0187	0.0020	-0.3284	-0.2542
Stimulus content [Control]	-0.4638	-0.4631	0.0190	0.0007	-0.4968	-0.4206
Gender [Male]	0.0498	0.0468	0.0171	-0.0031	0.0126	0.0806
Stimulus content [Positive] × Gender [Male]	-0.0189	-0.0165	0.0260	0.0024	-0.0660	0.0349
Stimulus content [Neutral] × Gender [Male]	-0.0117	-0.0091	0.0251	0.0026	-0.0566	0.0404
Stimulus content [Control] × Gender [Male]	-0.0477	-0.0456	0.0263	0.0021	-0.0983	0.0068

2.2.5 Total number of fixations

The Total Number of Fixations is a measure of sustained (delayed) attention. It reflects how often a stimulus was fixated upon throughout a trial, capturing the frequency with which a participant returns to or continues engaging with a stimulus. During the experiment, participants were allowed to explore freely, and the number of times each stimulus was fixated upon was recorded.

2.2.5.1 Fit model In the fifth model, the Total Number of Fixations is predicted by the type of stimulus and its interaction with the participant's gender. Random intercepts were included for both participants and stimuli, accounting for individual variability and stimulus-specific effects on the fixation count.

The model was estimated using a Gamma distribution with a log link, appropriate for positively skewed count-like data with non-integer values.

```
mod5_glmm <- glmmTMB(
  Fixation_count ~ Stimulus_content * Gender +
    (1 + Stimulus_content || Participant) +
    (1 | Stimulus),
  family = Gamma(link = "log"),
  data = bd
)
```

2.2.5.1.1 Model assumptions The following functions 'check_model()', 'check_distribution()', and 'check_normality()' are used to evaluate the quality of fit and distribution of residuals in a statistical model. For example, check_model() could provide a visualization of residuals and diagnose problems such as heteroscedasticity or bias in the model. check_distribution() could provide a visualization of the distribution of residuals and diagnose problems such as deviation from normality or outliers. .

```r
check_model(mod5_glmm)
```

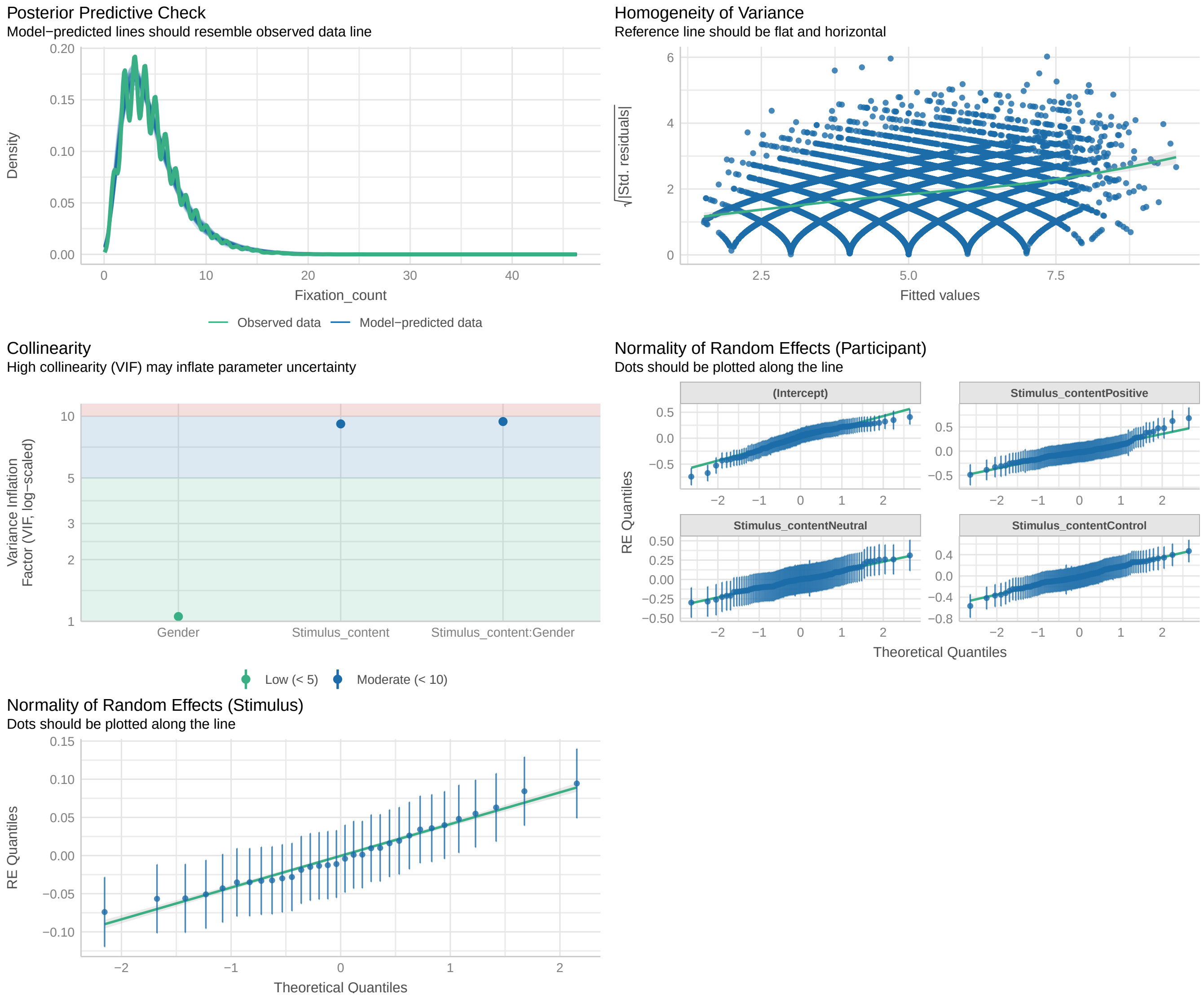


```r
dist_checkmod5 <- check_distribution(mod5_glmm)
      kable(data.frame(dist_checkmod5),
      digits = 2, booktabs = TRUE,
      caption = "Distribution of residuals model 4") %>%
      kable_styling(latex_options = c("HOLD_position"))
```

Table S31. *Distribution of residuals model 4*

Distribution	p_Residuals	p_Response
bernoulli	0.00	0.00
beta	0.00	0.00
beta-binomial	0.00	0.12
binomial	0.00	0.00
cauchy	0.44	0.00
chi	0.06	0.12
exponential	0.03	0.00
F	0.00	0.00
gamma	0.19	0.03
half-cauchy	0.03	0.00
inverse-gamma	0.00	0.00
lognormal	0.00	0.00
neg. binomial (zero-infl.)	0.00	0.03
negative binomial	0.00	0.56
normal	0.16	0.00
pareto	0.00	0.00
poisson	0.00	0.06
poisson (zero-infl.)	0.00	0.03
tweedie	0.00	0.00
uniform	0.00	0.00
weibull	0.09	0.03

2.2.5.1.2 Summary model To examine the results of model 5, we used three complementary summaries. First, we used the Anova() function from the car package to obtain a Type III Wald chi-square test, which evaluates the significance of each predictor and their interaction in the model. This method is appropriate for generalized linear mixed models fitted with glmmTMB.

Second, we applied the summary() function to obtain the estimated coefficients, their standard errors, z-values, and associated p-values for the fixed effects.

Third, we calculated the marginal and conditional pseudo R^2 values using the r2_nakagawa() function. These values reflect the proportion of variance explained by the fixed effects alone (marginal R^2) and by both fixed and random effects combined (conditional R^2), following the method proposed by Nakagawa and Schielzeth (Nakagawa & Schielzeth, 2013).

```
anova.mod5 <- Anova(mod5_glmm, type = 3)
anova.mod5 <- as.data.frame(anova.mod5)|>
  rownames_to_column() |>
  mutate_at("rowname", str_replace_all, ":", " × ") |>
  mutate_at("rowname", str_replace_all, "Stimulus_content",
        "Stimulus content")|>
  rename( Effect = rowname)|>
  kable(digits = 2, booktabs = TRUE,
      align = c("l", "l", rep("c", 4)), caption = "Effect of StimulusContent and Participant's Gender on
      escape = FALSE)|>
      kable_styling(latex_options = c("hold_position"))
anova.mod5
```

```
sum.mod5 <- summary(mod5_glmm)


sum.mod5_df <- tidy(mod5_glmm, effects = "fixed") |>
  mutate(term = str_replace_all(term, ":", " × ")) |>
```

Table S32. *Effect of StimulusContent and Participant's Gender on Total number of fixings (TNF)*

Effect	Chisq	Df	Pr(>Chisq)
(Intercept)	2543.99	1	0.00
Stimulus content	207.02	3	0.00
Gender	2.01	1	0.16
Stimulus content × Gender	3.60	3	0.31

```
  mutate(term = str_replace_all(term, "Stimulus_contentControl",
                                "Stimulus content [Control]")) |>
  mutate(term = str_replace_all(term, "Stimulus_contentPositive",
                                "Stimulus content [Positive]")) |>
  mutate(term = str_replace_all(term, "Stimulus_contentNeutral",
                                "Stimulus content [Neutral]")) |>
  mutate(term = str_replace_all(term, "GenderMale",
                                "Gender [Male]")) |>
  rename (Effect = term,
         Estimate = estimate,
         `Std. Error` = std.error,
         `z value` = statistic,
         `Pr(>|z|)` = p.value)|>
  select(Effect, Estimate, `Std. Error`, `z value`, `Pr(>|z|)`)

sum.mod5_df |>
  kable(digits = 2, booktabs = TRUE,
        align = c("l", rep("c", 5)),
        caption = "Total number of fixings (TNF)
        by stimylus content,gender and the
        interactionbetween stimulus contentand gender",
        escape = FALSE) |>
  kable_styling(latex_options = c("HOLD_position"))
```

Table S33. *Total number of fixings (TNF) by stimylus content,gender and the interactionbetween stimulus contentand gender*

Effect	Estimate	Std. Error	z value	Pr(>|z|)
(Intercept)	1.69	0.03	50.44	0.00
Stimulus content [Positive]	-0.09	0.03	-2.74	0.01
Stimulus content [Neutral]	-0.22	0.03	-8.46	0.00
Stimulus content [Control]	-0.41	0.03	-12.97	0.00
Gender [Male]	0.06	0.04	1.42	0.16
Stimulus content [Positive] × Gender [Male]	-0.05	0.05	-1.09	0.28
Stimulus content [Neutral] × Gender [Male]	-0.01	0.04	-0.42	0.68
Stimulus content [Control] × Gender [Male]	-0.07	0.04	-1.69	0.09

```
r2mod5 <- r2_nakagawa(mod5_glmm)
r2_tblmod5 <- as_tibble(r2mod5)
kable(r2_tblmod5, format = "markdown",  booktabs =TRUE,
      align = "c", caption = "R model 5",
      escape = FALSE) %>%
      kable_styling(latex_options = c("HOLD_position"))
```

Table S34. *R model 5*

R2_conditional	R2_marginal
0.3159274	0.0806485

```r
re_par <- parameters::model_parameters(mod5_glmm, effects = "random")
re_df  <- as.data.frame(re_par)

col_param <- intersect(c("Parameter","Term","Name"), names(re_df))[1]
col_est   <- intersect(c("SD","SD (random effect)","Coefficient","Estimate","Est."), names(re_df))[1]
col_group <- intersect(c("Group","Cluster"), names(re_df))[1]

tab_sd <- re_df %>%
  transmute(
    Group  = .data[[col_group]],
    Effect_raw = .data[[col_param]],
    SD   = suppressWarnings(as.numeric(.data[[col_est]]))
  ) %>%
  filter(grepl("^SD\\s*\\(", Effect_raw)) %>%
  mutate(
    Effect = str_replace(Effect_raw, "^SD\\s*\\((.*)\\)$", "\\1"),
    Effect = str_replace_all(Effect, "Stimulus_content", "Stimulus content"),
    Effect = str_replace_all(Effect, "First_fixation",   "Stimulus content"),
    Effect = str_replace_all(Effect, ":", " × "),
    Effect = str_replace(Effect, "Stimulus contentPositive", "Stimulus content [Positive]"),
    Effect = str_replace(Effect, "Stimulus contentNeutral",  "Stimulus content [Neutral]"),
    Effect = str_replace(Effect, "Stimulus contentControl",  "Stimulus content [Control]"),
    Variance = SD^2
  ) %>%
  select(Group, Effect, SD, Variance)

kable(tab_sd, digits = 4, booktabs = TRUE,
    caption = "Random effects (SD and Variance) - Model 5 (Gamma GLMM)") %>%
  kable_styling(latex_options = c("HOLD_position"))
```

Table S35. *Random effects (SD and Variance) — Model 5 (Gamma GLMM)*

Group	Effect	SD	Variance
Participant	Intercept	0.2292	0.0525
Stimulus	Intercept	0.0469	0.0022
Participant	Stimulus content [Positive]	0.2111	0.0446
Participant	Stimulus content [Neutral]	0.1482	0.0220
Participant	Stimulus content [Control]	0.2029	0.0412

2.2.5.1.3 Estimated marginal means Given the significant effect observed for the stimulus content variable in the ANOVA-type table, post-hoc pairwise comparisons were performed using the emmeans() function. These contrasts allow for a detailed examination of the differences between levels of Stimulus content (i.e., Control, Neutral, Positive, Negative), adjusting for multiple comparisons and accounting for the model's link function.

The estimated marginal means were computed on the response scale using type = "response" and regridded to obtain interpretable values (i.e., in milliseconds). The table below presents the pairwise contrasts between levels of Stimulus content.

```
mcm5 <- emmeans(mod5_glmm, pairwise ~ Stimulus_content, type = "response")
mcm5$emmeans <- regrid(mcm5$emmeans)
mcm5$contrasts <- pairs(mcm5$emmeans)

mcm5_emmeans <- tibble(data.frame(mcm5$emmeans)) |>
  rename(Fixation_count = response)|>
  select(-df)

contrasts_df5 <-  as.data.frame(mcm5$contrasts)|>
  select(-df)

kable(contrasts_df5, digits = 5,  booktabs =TRUE,
      align = "c", caption = "contrasts between variables, according to the
      content of the stimulus of model 5") %>%
      kable_styling(latex_options = "HOLD_position",
                    font_size = 12,
                    full_width = FALSE)
```

Table S36. *contrasts between variables, according to the content of the stimulus of model 5*

contrast	estimate	SE	z.ratio	p.value
Negative - Positive	0.60126	0.11521	5.21898	0.00000
Negative - Neutral	1.13446	0.08864	12.79872	0.00000
Negative - Control	2.01936	0.09742	20.72897	0.00000
Positive - Neutral	0.53320	0.12655	4.21325	0.00015
Positive - Control	1.41810	0.13081	10.84091	0.00000
Neutral - Control	0.88490	0.10303	8.58916	0.00000

2.2.5.2 Figure Finally, for this model 5, a figure showing the Total number of fixations as a function of stimulus content and the respective differences between the variables is shown.

```
tbl.contrasts_df5 <- contrasts_df5 %>%
  mutate(.y. = "Fixation_count") %>%
  separate_wider_delim(contrast, " - ",
                       names = c("group1", "group2")) %>%
  select(7, 1:6) %>%
  mutate(p.signif = pval.stars(p.value))

fig_tnf <- ggplot(bd, aes(x=Stimulus_content,
               y=Fixation_count)) +
  geom_violin(trim = FALSE,
              aes(fill = Stimulus_content)) +
  geom_jitter(alpha = 0.009, width = 0.3) +
  geom_errorbar(data = mcm5_emmeans,
              mapping =
                aes(ymin = Fixation_count-SE,
                    ymax = Fixation_count+SE),
                colour = "black", width = 0.01) +
  geom_point(data = mcm5_emmeans,
             shape = 50, size = 1,
             color = "black", fill = "white") +
  stat_pvalue_manual(tbl.contrasts_df5,
                     label = "p.signif",
                     y.position =  c(28.5, 32.5, 36.5, 30.5, 34.5, 28.5))  +
```

```
  labs(x = "Stimulus Content",
       y = "Total Number of Fixings (TNF)",
       fill = " ") +
  theme(axis.text.x = element_text(size = 10))+
  guides(fill = FALSE)+
  theme_tq()

fig_tnf
```

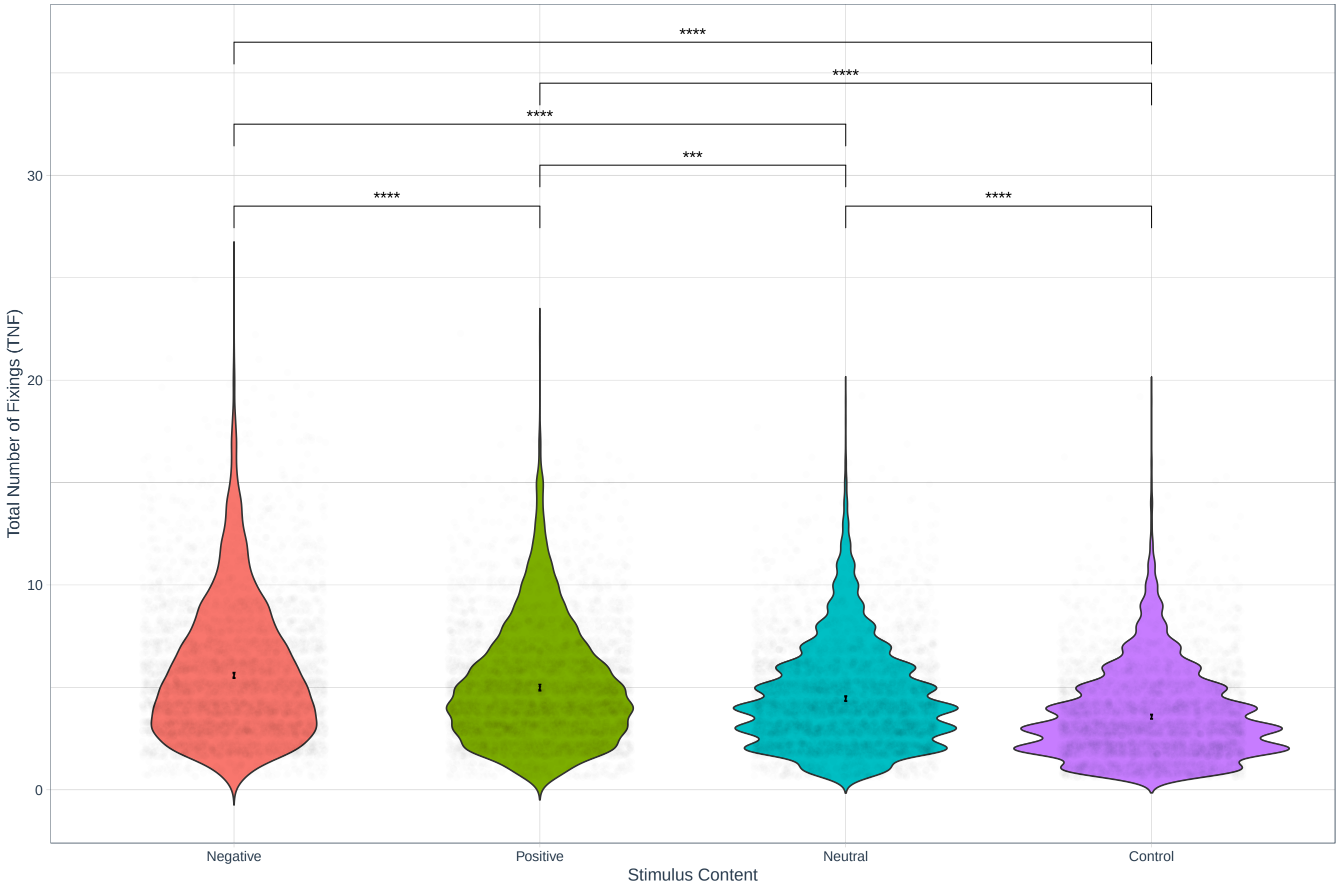

Figure S5. *Dimension between the emotional content of the stimulus, images with negative, positive, neutral and control emotional content, see Table S36 where the contrasts between each dimension are observed. In all cases, significant effects are represented with lines and stars:* *p < *.05,* **p < *.01,* ***p < *.001,* ****p < *.0001.*

2.2.5.3 Bootstrap Given that the model is a generalized linear mixed model with a non-normal error distribution (Gamma with log link), standard inferential techniques based on asymptotic normality may not provide reliable estimates. Therefore, a non-parametric bootstrap approach was used to better estimate the uncertainty associated with the fixed effects.

The bootstrap method involves generating multiple resamples from the original dataset (with replacement) and fitting the same model on each sample. This process produces an empirical sampling distribution of the statistic of interest, in this case, the Total Number of Fixings (TNF).

```
boot_fn <- function(data, indices) {
  d <- data[indices, ]
  fit <- glmmTMB(Fixation_count ~ Stimulus_content * Gender +
                   (1 + Stimulus_content | Participant) +
                   (1 | Stimulus),
                 data = d, family = Gamma(link = "log"))
```

```
  return(fixef(fit)$cond)
}

set.seed(824)
mod5_boot <- boot(data = bd1, statistic = boot_fn, R = 1000)

boot.ci <- boot.ci(mod5_boot, type = "basic", index = 1)
terms <- names(fixef(mod5_glmm)$cond)
boots_mod5_df <- data.frame(
  term = terms,
  observed = fixef(mod5_glmm)$cond,
  rep.mean = colMeans(mod5_boot$t),
  se = apply(mod5_boot$t, 2, sd),
  bias = colMeans(mod5_boot$t) - fixef(mod5_glmm)$cond
)

get_ci <- function(boot_obj, index) {
  ci <- boot.ci(boot_obj, type = "basic", index = index)
  if (!is.null(ci$basic)) {
    return(c(lower = ci$basic[4], upper = ci$basic[5]))
  } else {
    return(c(lower = NA, upper = NA))
  }
}


boot_cis <- t(sapply(1:length(terms), function(i) get_ci(mod5_boot, i)))
boots_mod5_df$ci.lower <- boot_cis[, "lower"]
boots_mod5_df$ci.upper <- boot_cis[, "upper"]


boots_mod5_df <- boots_mod5_df |>
  mutate(term = str_replace_all(term, ":", " × ")) |>
  mutate(term = str_replace_all(term, "Stimulus_contentControl", "Stimulus content [Control]")) |>
  mutate(term = str_replace_all(term, "Stimulus_contentPositive", "Stimulus content [Positive]")) |>
  mutate(term = str_replace_all(term, "Stimulus_contentNeutral", "Stimulus content [Neutral]")) |>
  mutate(term = str_replace_all(term, "GenderMale", "Gender [Male]"))

orden_deseado <- c("(Intercept)",
                   "Stimulus content [Positive]",
                   "Stimulus content [Neutral]",
                   "Stimulus content [Control]" ,
                   "Gender [Male]",
                   "Stimulus content [Positive] × Gender [Male]",
                   "Stimulus content [Neutral] × Gender [Male]",
                   "Stimulus content [Control] × Gender [Male]")

boots_mod5_df <- boots_mod5_df |> arrange(match(term, orden_deseado))

rownames(boots_mod5_df) <- NULL

boots_mod5_df |>
  select(term, observed, rep.mean, se, bias, ci.lower, ci.upper) |>
  rename(
    Effect = term,
    Estimate = observed,
```

```
    `Boot Mean` = rep.mean,
    `Std. Error` = se,
    Bias = bias,
    `CI Lower` = ci.lower,
    `CI Upper` = ci.upper
  ) |>
  kable(digits = 4, booktabs = TRUE,
        caption = "Bootstrap estimates of model 5",
        align = "lcccccc",
         rownames = FALSE) |>
  kable_styling(latex_options = c("HOLD_position"))
```

Table S37. *Bootstrap estimates of model 5*

Effect	Estimate	Boot Mean	Std. Error	Bias	CI Lower	CI Upper
(Intercept)	1.6890	1.6817	0.0116	-0.0073	1.6676	1.7139
Stimulus content [Positive]	-0.0893	-0.0863	0.0165	0.0030	-0.1180	-0.0543
Stimulus content [Neutral]	-0.2194	-0.2167	0.0172	0.0027	-0.2491	-0.1822
Stimulus content [Control]	-0.4113	-0.4098	0.0176	0.0015	-0.4419	-0.3731
Gender [Male]	0.0636	0.0603	0.0158	-0.0033	0.0310	0.0909
Stimulus content [Positive] × Gender [Male]	-0.0490	-0.0457	0.0237	0.0033	-0.0959	-0.0015
Stimulus content [Neutral] × Gender [Male]	-0.0150	-0.0121	0.0231	0.0029	-0.0564	0.0358
Stimulus content [Control] × Gender [Male]	-0.0742	-0.0704	0.0240	0.0037	-0.1180	-0.0250

3 Summary tables and figures

The objective of this section is to present the main figures and tables of the study. Figure 1 corresponds to the experimental design. Following this, we present a summary of the results obtained, grouping the main information into two tables and two figures. Table 1 and Figure 2 contain the information for early attention, that is, model 1 (Time to First Fixation, TFF), model 2), (First Fixation Duration, FFD), and model 3 (First Fixation Count, FFC). Table 2 and Figure 3 contain the main information for late attention, that is, model 4 (Total Duration of Fixations, TDF) and model 5 (Total Number of Fixations, TNF).

```
as.ggplot(image_read_pdf("Data/design.pdf"))
```

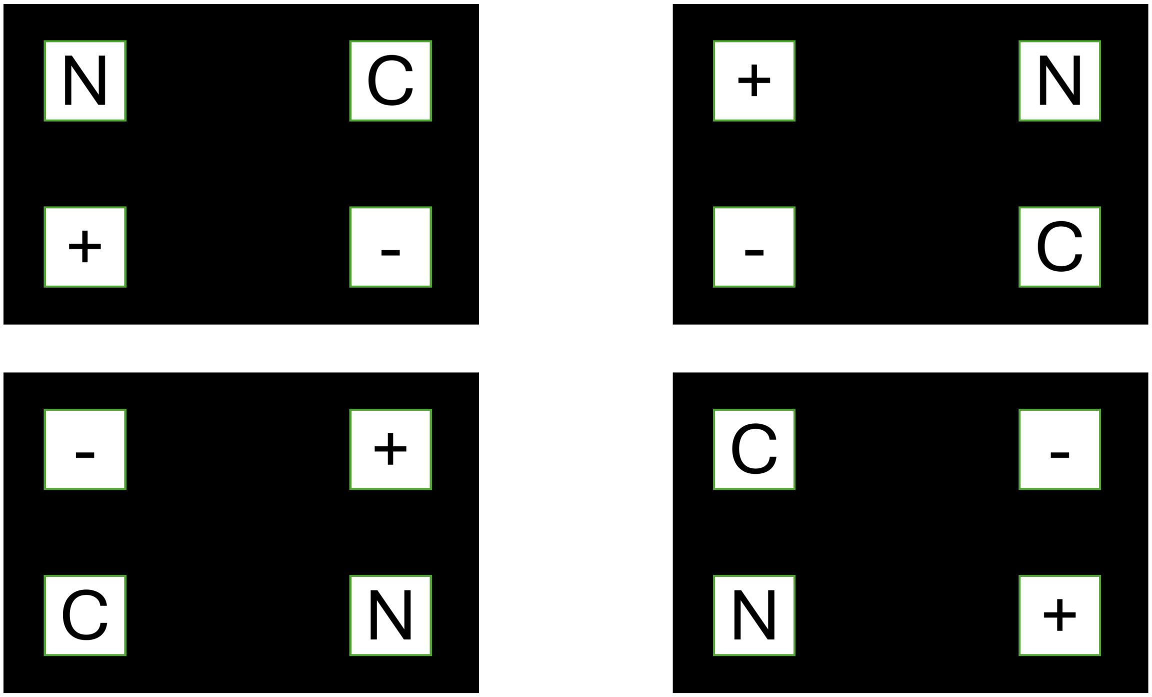

Figure 1. *Experimental design of image content*

The image of the experimental design is presented below, N corresponds to neutral images, C corresponds to control images, + corresponds to images with positive valence, and - corresponds to images with negative valence. To find the final stimuli, you can enter the following link https://osf.io/c89nx/files/osfstorage/68280df2adaf95d2f0cacbc7

```
# --- helpers locales solo para este chunk ---
tex_escape <- function(x) {
  x <- as.character(x)
  x <- gsub("\\\\", "\\\\textbackslash{}", x)
  x <- gsub("([#%&{}])", "\\\\\\1", x, perl = TRUE)
  x <- gsub("\\$", "\\\\$", x)
  x <- gsub("\\^", "\\\\^{}", x)
  x <- gsub("~", "\\\\textasciitilde{}", x)
  x <- gsub("_", "\\\\_", x, fixed = TRUE)
  x <- gsub("\\|", "\\\\textbar{}", x)
  x
}

fix_names <- function(aovtab){
  rn <- rownames(aovtab)
  rn <- gsub("^First_fixation$", "Stimulus content", rn)
  rn <- gsub("^Stimulus_content$", "Stimulus content", rn)
  rn <- gsub("First_fixation:Gender", "Stimulus content × Gender", rn)
  rn <- gsub("Stimulus_content:Gender", "Stimulus content × Gender", rn)
  rownames(aovtab) <- rn
  aovtab
}

# --- Anovas con nombres ya corregidos ---
m1 <- fix_names(Anova(mod1_glmm, type = 3))
```

```
m2 <- fix_names(Anova(mod2_glmm, type = 3))
m3 <- fix_names(Anova(mod3, type = 3))
m4 <- fix_names(Anova(mod4_glmm, type = 3))
m5 <- fix_names(Anova(mod5_glmm,type = 3))

# --- Tablas combinadas ---
tab1 <- MultMerge(m1, m2, m3)
tab2 <- MultMerge(m4, m5)

# Limpieza extra de nombres
rn1 <- rownames(tab1)
rn1 <- sub("^First_fixation$", "Stimulus content", rn1)
rn1 <- sub("^First_fixation:Gender$", "Stimulus content × Gender", rn1)
rownames(tab1) <- rn1

rn2 <- rownames(tab2)
rn2 <- sub("^First_fixivation$", "Stimulus content", rn2)
rn2 <- sub("^First_fixation$", "Stimulus content", rn2)
rn2 <- sub("^First_fixation:Gender$", "Stimulus content × Gender", rn2)
rownames(tab2) <- rn2

# Encabezados con matemáticas (escape=FALSE)
colnames(tab1) <- c("$\\chi^2$", "df", "p", "$\\chi^2$", "df", "p", "$\\chi^2$", "df", "p")
colnames(tab2) <- c("$\\chi^2$", "df", "p", "$\\chi^2$", "df", "p")

# --- Mover rownames a columna de forma segura ---
tab1_df <- as.data.frame(tab1, check.names = FALSE)
tab1_df <- tibble::add_column(tab1_df,
                              Effect = tex_escape(rownames(tab1_df)),
                              .before = 1,
                              .name_repair = "minimal")

tab2_df <- as.data.frame(tab2, check.names = FALSE)
tab2_df <- tibble::add_column(tab2_df,
                              Effect = tex_escape(rownames(tab2_df)),
                              .before = 1,
                              .name_repair = "minimal")

# --- Imprimir con kable ---
kable(tab1_df,
      digits = 2,
      booktabs = TRUE,
      align = c("l", rep("c", ncol(tab1_df) - 1)),
      caption = "Overall results - Early attention",
      escape = FALSE) |>
  add_header_above(c(" " = 1, "Model 1" = 3, "Model 2" = 3, "Model 3" = 3)) |>
  add_header_above(c(" " = 1, "Early attention" = 9)) |>
  kable_styling(latex_options = c("HOLD_position"))
```

Table 1. *Overall results — Early attention*

	Early attention									
	Model 1			Model 2			Model 3			
	Effect	χ^2	df	p	χ^2.1	df.1	p.1	χ^2.2	df.2	p.2
(Intercept)	(Intercept)	59.47	1	0.00	3266.08	1	0.00	6043.41	1	0.00
Gender	Gender	0.91	1	0.34	1.73	1	0.19	2.13	1	0.14
Stimulus content	Stimulus content	97.55	3	0.00	67.88	3	0.00	158.86	3	0.00
Stimulus content × Gender	Stimulus content × Gender	1.75	3	0.62	3.01	3	0.39	9.03	3	0.03

```
kable(tab2_df,
      digits = 2,
      booktabs = TRUE,
      align = c("l", rep("c", ncol(tab2_df) - 1)),
      caption = "Overall results - Late attention",
      escape = FALSE) |>
  add_header_above(c(" " = 1, "Model 4" = 3, "Model 5" = 3)) |>
  add_header_above(c(" " = 1, "Late attention" = 6)) |>
  kable_styling(latex_options = c("HOLD_position"))
```

Table 2. *Overall results — Late attention*

	Late attention						
	Model 4			Model 5			
	Effect	χ^2	df	p	χ^2.1	df.1	p.1
(Intercept)	(Intercept)	213.24	1	0.00	2543.99	1	0.00
Gender	Gender	1.01	1	0.32	2.01	1	0.16
Stimulus content	Stimulus content	200.24	3	0.00	207.02	3	0.00
Stimulus content × Gender	Stimulus content × Gender	0.84	3	0.84	3.60	3	0.31

```
ggarrange(fig_TPF, fig_DFF, fig_ffc_female, fig_ffc_male,
          nrow = 2,
          ncol = 2,
          labels = c("a","b","c",""),
          common.legend = TRUE,
          legend = "bottom")
```

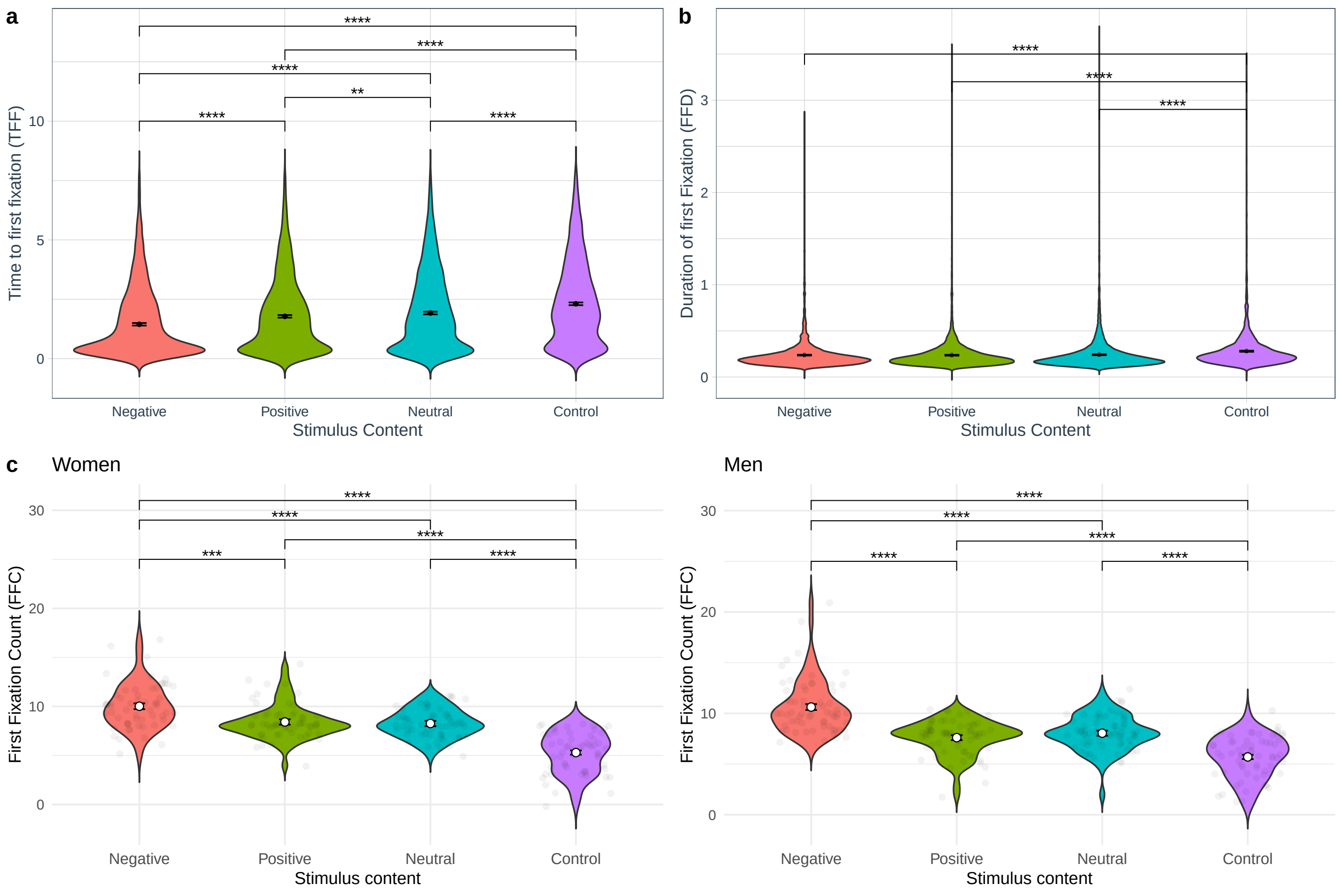

Figure 2. *Early attention. Dimension between the emotional content of the stimulus, images with negative, positive, neutral and control emotional content, where the contrasts between each dimension are observed. Considering that for model 3 (first fixation count) the interaction of gender with stimulus content was significant, separate graphs by gender are presented. In all cases, significant effects are represented with lines and stars:* *p < .05, **p < .01, ***p < .001, ****p < .0001.

```
ggarrange(fig_tdf, fig_tnf,
          nrow = 1,
          ncol = 2,
          labels = c("a", "b"),
          common.legend = TRUE,
          legend = "bottom")
```

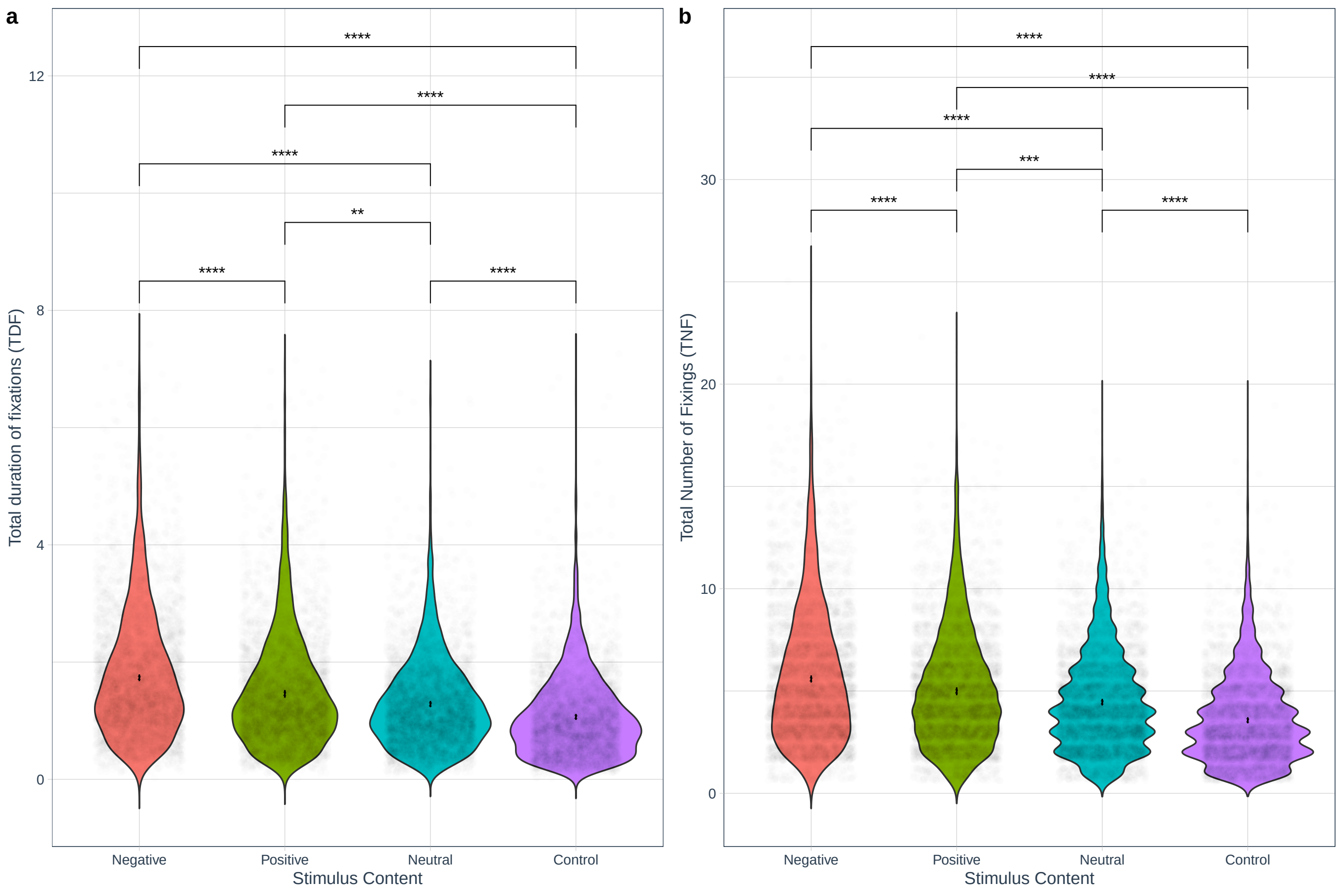

Figure 3. *Late attention. Dimension between the emotional content of the stimulus, images with negative, positive, neutral and control emotional content, where the contrasts between each dimension are observed. In all cases, significant effects are represented with lines and stars:* *p < .05, **p < .01, ***p < .001, ****p < .0001.

4 Session info (for reproducibility)

```
library(pander)
pander(sessionInfo(), locale = FALSE)
```

R version 4.5.0 (2025-04-11 ucrt)

Platform: x86_64-w64-mingw32/x64

attached base packages: *stats*, *graphics*, *grDevices*, *utils*, *datasets*, *methods* and *base*

other attached packages: *pander(v.0.6.6)*, *DHARMa(v.0.4.7)*, *DescTools(v.0.99.60)*, *broom.mixed(v.0.2.9.6)*, *car(v.3.1-3)*, *carData(v.3.0-5)*, *glmmTMB(v.1.1.11)*, *pdftools(v.3.5.0)*, *ggplotify(v.0.1.2)*, *magick(v.2.8.6)*, *PerformanceAnalytics(v.2.0.8)*, *quantmod(v.0.4.27)*, *TTR(v.0.24.4)*, *xts(v.0.14.1)*, *zoo(v.1.8-14)*, *tidyquant(v.1.0.11)*, *boot(v.1.3-31)*, *gtools(v.3.9.5)*, *ggpubr(v.0.6.0)*, *broom(v.1.0.8)*, *kableExtra(v.1.4.0)*, *emmeans(v.1.11.1)*, *readxl(v.1.4.5)*, *performance(v.0.13.0)*, *lmerTest(v.3.1-3)*, *lme4(v.1.1-37)*, *Matrix(v.1.7-3)*, *lubridate(v.1.9.4)*, *forcats(v.1.0.0)*, *stringr(v.1.5.1)*, *dplyr(v.1.1.4)*, *purrr(v.1.0.4)*, *readr(v.2.1.5)*, *tidyr(v.1.3.1)*, *tibble(v.3.2.1)*, *ggplot2(v.3.5.2)*, *tidyverse(v.2.0.0)* and *knitr(v.1.50)*

loaded via a namespace (and not attached): *Rdpack(v.2.6.4)*, *gld(v.2.6.7)*, *rlang(v.1.1.6)*, *magrittr(v.2.0.3)*, *furrr(v.0.3.1)*, *e1071(v.1.7-16)*, *compiler(v.4.5.0)*, *mgcv(v.1.9-1)*, *systemfonts(v.1.2.3)*, *vctrs(v.0.6.5)*, *quadprog(v.1.5-8)*, *pkgconfig(v.2.0.3)*, *fastmap(v.1.2.0)*, *backports(v.1.5.0)*, *labeling(v.0.4.3)*, *rmarkdown(v.2.29)*, *tzdb(v.0.5.0)*, *haven(v.2.5.4)*, *nloptr(v.2.2.1)*, *tinytex(v.0.57)*, *xfun(v.0.52)*, *parallel(v.4.5.0)*, *R6(v.2.6.1)*, *stringi(v.1.8.7)*, *RColorBrewer(v.1.1-3)*, *parallelly(v.1.44.0)*,

cellranger(v.1.1.0), *numDeriv(v.2016.8-1.1)*, *estimability(v.1.5.1)*, *Rcpp(v.1.0.14)*, *bookdown(v.0.43)*, *parameters(v.0.25.0)*, *splines(v.4.5.0)*, *timechange(v.0.3.0)*, *tidyselect(v.1.2.1)*, *rstudioapi(v.0.17.1)*, *abind(v.1.4-8)*, *yaml(v.2.3.10)*, *codetools(v.0.2-20)*, *TMB(v.1.9.17)*, *curl(v.6.2.2)*, *listenv(v.0.9.1)*, *qpdf(v.1.3.5)*, *lattice(v.0.22-6)*, *bayestestR(v.0.15.3)*, *withr(v.3.0.2)*, *askpass(v.1.2.1)*, *coda(v.0.19-4.1)*, *evaluate(v.1.0.3)*, *gridGraphics(v.0.5-1)*, *future(v.1.49.0)*, *proxy(v.0.4-27)*, *xml2(v.1.3.8)*, *pillar(v.1.10.2)*, *reformulas(v.0.4.1)*, *insight(v.1.2.0)*, *generics(v.0.1.4)*, *hms(v.1.1.3)*, *rootSolve(v.1.8.2.4)*, *scales(v.1.4.0)*, *minqa(v.1.2.8)*, *globals(v.0.18.0)*, *xtable(v.1.8-4)*, *class(v.7.3-23)*, *glue(v.1.8.0)*, *lmom(v.3.2)*, *tools(v.4.5.0)*, *data.table(v.1.17.2)*, *ggsignif(v.0.6.4)*, *Exact(v.3.3)*, *fs(v.1.6.6)*, *mvtnorm(v.1.3-3)*, *cowplot(v.1.1.3)*, *grid(v.4.5.0)*, *rbibutils(v.2.3)*, *datawizard(v.1.1.0)*, *RobStatTM(v.1.0.11)*, *nlme(v.3.1-168)*, *Formula(v.1.2-5)*, *cli(v.3.6.5)*, *textshaping(v.1.0.1)*, *expm(v.1.0-0)*, *viridisLite(v.0.4.2)*, *svglite(v.2.2.1)*, *gtable(v.0.3.6)*, *rstatix(v.0.7.2)*, *yulab.utils(v.0.2.0)*, *digest(v.0.6.37)*, *farver(v.2.1.2)*, *htmltools(v.0.5.8.1)*, *lifecycle(v.1.0.4)*, *httr(v.1.4.7)* and *MASS(v.7.3-65)*

5 Supplementary references

Bolker, B., Warnes, G., & Lumley, T. (2022). *Gtools: Various r programming tools* [R package version 3.9.4]. https://CRAN.R-project.org/package=gtools

Brooks, M. E., Kristensen, K., van Benthem, K. J., Magnusson, A., Berg, C. W., Nielsen, A., Skaug, H. J., Mächler, M., & Bolker, B. M. (2017). Glmmtmb balances speed and flexibility among packages for zero-inflated generalized linear mixed modeling. *The R Journal*, *9*(2), 378–400. https://journal.r-project.org/archive/2017/RJ-2017-066/index.html

Canty, A., & Ripley, B. (2022). *Boot: Bootstrap functions (originally by angelo canty for s)* [R package version 1.3-28.1]. https://CRAN.R-project.org/package=boot

Dancho, M., & Vaughan, D. (2023). *Tidyquant: Tidy quantitative financial analysis* [R package version 3.5.0]. https://cran.r-project.org/web/packages/tidyquant/index.html

Hartig, F. (2024). *Dharma: Residual diagnostics for hierarchical (multi-level / mixed) regression models* [R package version 0.4.7]. https://github.com/florianhartig/DHARMa/issues

Kassambara, A. (2023). *Ggpubr: 'ggplot2' based publication ready plots* [R package version 0.6.0]. https://CRAN.R-project.org/package=ggpubr

Kurdi, B., Lozano, S., & Banaji, M. R. (2017). Introducing the Open Affective Standardized Image Set (OASIS). *Behavior Research Methods*, *49*(2), 457–470. https://doi.org/10.3758/s13428-016-0715-3

Lenth, R. V. (2022). *Emmeans: Estimated marginal means, aka least-squares means* [R package version 1.7.3]. https://CRAN.R-project.org/package=emmeans

Lüdecke, D., Ben-Shachar, M. S., Patil, I., Waggoner, P., & Makowski, D. (2021). performance: An R package for assessment, comparison and testing of statistical models. *Journal of Open Source Software*, *6*(60), 3139. https://doi.org/10.21105/joss.03139

Marchewka, A., Żurawski, Ł., Jednoróg, K., & Grabowska, A. (2014). The Nencki Affective Picture System (NAPS): Introduction to a novel, standardized, wide-range, high-quality, realistic picture database. *Behavior Research Methods*, *46*(2), 596–610. https://doi.org/10.3758/s13428-013-0379-1

Nakagawa, S., & Schielzeth, H. (2013). A general and simple method for obtaining r2 from generalized linear mixed-effects models. *Methods in Ecology and Evolution*, *4*(2), 133–142. https://doi.org/https://doi.org/10.1111/j.2041-210x.2012.00261.x

Robinson, D. (2020). *Broom: Convert statistical objects into tidy tibbles* [R package version 0.7.6]. https://CRAN.R-project.org/package=broom

Signorell, A. (2023). *Desctools: Tools for descriptive statistics* [R package version 0.99.23]. https://cran.r-project.org/web/packages/DescTools/index.html

Wickham, H. (2016). *Ggplot2: Elegant graphics for data analysis*. Springer-Verlag New York. https://ggplot2.tidyverse.org

Wickham, H., Averick, M., Bryan, J., Chang, W., McGowan, L. D., François, R., Grolemund, G., Hayes, A., Henry, L., Hester, J., Kuhn, M., Pedersen, T. L., Miller, E., Bache, S. M., Müller, K., Ooms, J., Robinson, D., Seidel, D. P., Spinu, V., . . . Yutani, H. (2019). Welcome to the tidyverse. *Journal of Open Source Software*, *4*(43), 1686. https://doi.org/10.21105/joss.01686

Wickham, H., & Bryan, J. (2021). *Readxl: Read excel files* [R package version 1.3.1]. https://CRAN.R-project.org/package=readxl

Wickham, H., François, R., Henry, L., & Müller, K. (2022). *Dplyr: A grammar of data manipulation* [R package version 1.0.8]. https://CRAN.R-project.org/package=dplyr

Wickham, H., Vaughan, D., Girlich, M., Ushey, K., Posit, & PBC. (2023). *Tidyr: Tidy messy data* [R package version 1.3.0]. https://CRAN.R-project.org/package=tidyr

Wolen, A. R., Hartgerink, C. H., Hafen, R., Richards, B. G., Soderberg, C. K., & York, T. P. (2020). osfr: An R interface to the open science framework. *Journal of Open Source Software*, *5*(46), 2071. https://doi.org/10.21105/joss.02071

Xie, Y. (2014). Knitr: A comprehensive tool for reproducible research in R [ISBN 978-1466561595]. In V. Stodden, F. Leisch, & R. D. Peng (Eds.), *Implementing reproducible computational research*. Chapman and Hall/CRC. https://doi.org/10.1201/9781315373461-1

Zhu, H. (2020). *Kableextra: Construct complex table with 'kable' and pipe syntax* [R package version 1.3.1]. https://CRAN.R-project.org/package=kableExtra
