# Supplementary material for: Effects of stimulus emotional content on gaze pattern: An eye-tracking study
Source: PLoS One. 2026 Jan 28;21(1):e0341261. doi: 10.1371/journal.pone.0341261 (PMC12851487; doi:10.1371/journal.pone.0341261)
Supplement: Attachment — Submitted filename: Response to Reviewers.pdf [file pone.0341261.s003.pdf]

Manuscript ID: PONE-D-24-50575
Title: *Effects of stimulus emotional content on gaze pattern: an eye-tracking study*
Dear Dr. Elisa Scerrati,

We would like to express our sincere thanks to you and the reviewers for the time and effort spent in evaluating our manuscript. We sincerely appreciate the constructive comments and insightful suggestions, which have been invaluable in improving the clarity, rigor, and overall quality of our study.

We have carefully considered each of the issues raised and have substantially revised the manuscript to address them. Below, we provide a detailed point-by-point response to each of the reviewers' comments, explaining how each issue has been addressed. In particular, we have

- Clarified the ethical procedures and provided a detailed rationale on the informed consent process.
- Added a rationale for the sample size based on the decision that was made at the time.
- Given an interpretation of the results from the Bootstrap analyses, taking into account the violation of assumptions.
- Improved consistency between results and interpretation, and moderated speculative statements.

We would like to thank reviewer #1 for recognizing the strengths of the paper and for pointing out aspects that needed clarification. His comments helped us refine our theoretical framing and methodological clarity.

We are also especially grateful to reviewer #2, whose detailed and rigorous comments led us to reexamine several aspects of our analysis and interpretation. His input was instrumental in improving the robustness and consistency of the interpretation of our results and conclusions.
We believe that the revised version of the manuscript has been significantly strengthened, and we hope that the changes we have made meet the standards for publication in PLOS ONE

- We respectfully submit our revised manuscript along with:
- A corrected version with the changes made,
- A clean version of the revised manuscript, and
- The detailed point-by-point response document.

Thank you again for the opportunity to review our work and for considering our responses. We remain at your disposal for any further clarification or revision needed.

Sincerely,
Andrés Castellanos-Chacón
On behalf of all co-authors
Universidad El Bosque
gcastellanos@unbosque.edu.co

Reviewer #1:

We sincerely appreciate your positive review and valuable comments. We have carefully considered each of his observations and believe that, thanks to them, the manuscript has been significantly strengthened. Their contributions allowed us to improve conceptual clarity, optimize the structure of the introduction, broaden the interpretive depth of the discussion, and reinforce methodological transparency. We especially appreciate the suggestions related to statistical justification, ethical management of the information and the inclusion of sociocultural factors, which substantially enriched the focus of the study.

Abstract

- statistical analysis should be reported in the abstract.

In response to this point, as well as some recommendations made by Reviewer 2, we completely rewrote the abstract. It now reports the main statistical analyses (p. 1-2):

> *The attentional system tends to prioritize negative stimuli in the early stages of processing, favoring threat detection. However, it is unclear whether this bias is maintained or reversed toward positive stimuli at later stages. In this study, we used a free-viewing paradigm with eye tracking to examine early and late attentional biases toward negative, positive, and neutral stimuli (humans in emotionally unloaded activities) versus control stimuli (inanimate objects) in 122 participants without affective disorders (64 men, 58 women). Mixed models evidenced that participants fixated faster ($F_{3, 15156.24} = 186.43$, $p < .001$) and more first times ($F_{3, 15157.50} = 56.93$, $p < .001$) on negative stimuli compared to the other stimuli, confirming a negativity bias in early attention. In late attention, we observed a longer total fixation time on negative stimuli ($F_{3,15178.97} = 451.78$, $p < .001$) and a greater total number of fixations ($F_{3, 15155.66} = 447.39$, $p < .001$), contradicting the hypothesis of a positive bias in emotional regulation. In addition, we found significant differences in the interaction between emotional content and gender in delayed attention ($p < .001$), although no direct differences were found between women and men. This sustained negativity bias may reflect an adaptive regulatory process in which individuals allocate attentional resources to threat-related information to enhance learning and emotional preparedness. One limitation of the study is the lack of control over socioecological factors that may shape attentional biases, such as cultural normalization of violence. Future studies should examine these effects across diverse sociocultural settings and in clinical populations.*

Introduction

- The introduction is generally lengthy, and some sections may need to be summarized to allow the reader to grasp the key points more quickly.

We have abridged several sections to improve readability and ensure that key points are presented more concisely. Specifically, we have refined the theoretical framework by reducing redundancies in the discussion of attentional biases and inhibitory processes, retaining only the most relevant definitions and conceptual distinctions. In addition, we condensed the discussion of early and late attentional biases, preserving the context necessary to support the research question. The section on

eye-tracking was also revised to emphasize its methodological relevance and highlight its advantages in capturing temporal aspects of attention. Most of these changes are detailed in the following responses to both reviewers. The introduction was reduced from 1587 words to a total of 1082 words, which is ~30% less, and within this total we additionally discussed what was requested at other points.

- Some concepts, such as "bias" and "inhibitory processes," could be defined more clearly.

We have better defined these two concepts:

> Bias (p. 2): *However, our brain can attend to stimuli or ignore them, depending on the load assigned to a stimulus, and this is known as bias, which in a general sense, refers to a systematic deviation in the processing of the information we attend to and which leads us to have a preference for certain stimuli over others (6).*
> Inhibitory processes (p. 3): *In addition, inhibitory processes play a crucial role in the regulation of attention, as they allow individuals to suppress irrelevant or distracting stimuli in favor of more relevant information (10).*

Materials and Methods

- While it is mentioned that the sample size was increased based on previous studies, the lack of determination of the sample size based on statistical power could undermine the validity of the results. Further explanation in this regard could be beneficial.

We have added the following paragraph (p. 7) to our article, where we acknowledge the limitation that the lack of an a priori power analysis creates. However, we have also included a subsequent power analysis to show that our sample was larger than the initial sample estimate based on another study, improving over previous research in this regard.

> *Given the within-subject design and repeated-measures structure of the eye-tracking data, we expect high sensitivity for detecting attentional biases. However, we acknowledge the limitation of our study and the importance of power analyses in study planning, and encourage future research to incorporate a priori power calculations to further strengthen the robustness of the findings. Although we acknowledge this limitation, a post hoc power analysis was performed on the data from the Sears et al. (51) , the only previous study that presents 4 simultaneous images, using the pwr package in R. The analysis revealed that, with $u = 2$ degrees of freedom in the numerator and $v = 78.03$ degrees of freedom in the denominator, the effect size was $f^2 = 0.12$. Given a significance level of $\alpha = 0.05$, an adequate probability of detecting significant effects of 0.80 would be achieved with a sample of 81 participants. Based on these assumptions, our sample size of $n = 122$ provides a power of 0.94.*

- While the approval from the ethics committee is mentioned, additional details regarding the management of personal information and assurance of informed consent from participants could enhance transparency.

Thanks. We have expanded information on the use of covert informed consent, explaining its importance for research and supporting it with an article by Riege et al. (2021) that discusses the ethical implications of this method and provides a methodological and ethical precedent for this (p. 14-15).

> *The study protocol was approved by the institutional ethics committee of Universidad El Bosque (PIS 002-2020). A first informed consent form was submitted virtually, in order to obtain preliminary data that would allow us to discard those participants who did not meet the inclusion criteria of the research. Subsequently, participants who met the criteria reported to the laboratory the following day. In the laboratory, participants signed a covert written consent form, in which participants were not initially informed that their eye movements were being recorded. Personal data were treated confidentially and anonymously to ensure privacy and compliance with data protection regulations. Only aggregated data were used in the analyses and no personally identifiable information was retained. Once the experimental paradigm was completed, full informed consent was provided, explaining the experiment in detail and, in the event of not wishing to participate, ensuring the deletion of all data collected.*
>
> *We recognize that the use of covert consent procedures poses significant ethical challenges. However, in the context of this study, this approach was deemed necessary to ensure the scientific validity of the results while minimizing any potential risk to participants. Throughout the process, specific measures were taken to safeguard the autonomy of the participants and to respect ethical standards.*
>
> *In order not to disrupt natural patterns of visual scanning, participants were not initially informed that their eye movements were being recorded. This temporary omission was approved by the institutional ethics committee of Universidad El Bosque (approval number PIS 002-2020), based on the following considerations: (1) the risk to participants was minimal, as only gaze patterns were collected in response to emotionally varied but noninvasive visual stimuli, and no personal biometric data beyond gaze location were recorded; (2) a thorough debriefing and full informed consent was conducted immediately after the task, during which participants were given the opportunity to withdraw their data without penalty; and (3) early disclosure of the specific study objectives would likely have compromised the validity of the data by altering participants' naturalistic visual behavior. This approach aligns with recent methodological perspectives that support the ethical use of covert eye-tracking procedures under controlled conditions, particularly when transparency may compromise the authenticity of participants' responses. For example, Riege et al. (59) argue that covert eye-tracking can be ethically justified when accompanied by robust debriefing protocols and voluntary data withdrawal options, as it allows researchers to investigate cognitive and attentional processes without introducing reactivity or demand characteristics.*
>
> *In applying this design, we attempted to balance the need to reduce experimental bias with the ethical imperative to respect participants' rights. No participants expressed discomfort or chose to withdraw after receiving full information about the study, suggesting that the measures taken were effective in maintaining both scientific integrity and ethical responsibility.*

- While it is noted that the study was not pre-registered, further explanation regarding the reasons for this and its impact on the validity of the results could be beneficial.

We have added the following information to the Transparency and Openness section (p. 13):

> *This study was not pre-registered due to a lack of information on pre-registration requirements at the time the study was initiated, and because it was not a standard procedure within our research framework when the study was designed. Despite this, we have made efforts to guarantee transparency by providing detailed methods and reproducible analyses, including the sample size justification, stimulus selection, and the complete analysis code along with the data in the Supplementary Material. Furthermore, all hypotheses and statistical models were specified to the ethics committee prior to data collection, minimizing potential bias in the analysis.*

Discussion

- While the results indicate that late attention was greater towards negative content than positive, a deeper interpretation of the reasons behind these findings and their potential impacts on human behavior could have enriched the discussion.

We have now provided an interpretation of Late Attention and the neurocognitive and evolutionary mechanisms that could explain sustained attentional engagement with negative stimuli. To this end, we present an analysis of how the brain's attentional systems, including the amygdala, adapt to detect and monitor potential threats in the environment. We also highlight evidence showing that negative stimuli tend to generate more intense autonomic and cortical responses, which contribute to prolonged fixation (Schupp et al., 2004).

Importantly, rather than suggesting pathological implications, we emphasize that this attentional bias toward negative content likely reflects an adaptive, evolutionarily conserved mechanism to enhance learning and preparation. To illustrate how these biases manifest in everyday life, we cite studies showing that people in the general population tend to pay more attention to emotionally intense or threatening media content (Pourtois et al., 2013; Soroka et al., 2019), and that this tendency can shape emotional habits and influence people's interactions with their environment (p. 26-27).

> *Several studies suggest that sustained attention to negative stimuli may reflect an adaptive mechanism rooted in the evolutionary role of the attentional system to prioritize potentially threatening information (21,66). The amygdala, in particular, plays a key role in this process, enhancing the perceptual salience of emotionally salient stimuli and contributing to prolonged attentional engagement (67,68). This bias toward negative information has been linked to stronger autonomic and cortical responses (69), which may explain the sustained fixation observed in our findings.*
> *In everyday contexts, this attentional pattern may help explain why people are naturally drawn to emotionally intense or distressing news, images, or events. From an adaptive standpoint, prolonged attention to such content might support learning from aversive experiences and preparing for future threats. These natural biases could shape how individuals engage with emotionally charged information in their environment, possibly influencing emotional awareness, information-seeking behaviors, or even the salience of emotionally significant social cues (70,71).*

- Although the authors have mentioned social influences, examining cultural and environmental impacts on attention patterns and the interpretation of results could have enriched the discussion.

In the Restrictions on Generality section (p. 33), we have discussed how contextual variables such as exposure to violence (Lambert et al., 2010; McLaughlin et al., 2014), urban versus rural upbringing (Javanbakht et al., 2015), and cultural norms surrounding emotional expression (Butler et al., 2007; Matsumoto et al., 2008) could significantly modulate attentional responses to negative stimuli. Similarly, we present how the social context of Colombia can influence the results of our study. These factors can alter the salience of emotional content and thresholds for active or avoidant attention. For example, people exposed to chronic interpersonal violence may show heightened vigilance toward threatening stimuli, while those belonging to collectivist cultures may exhibit greater attentional disengagement due to norms surrounding emotional suppression. Therefore, while our findings reveal a general pattern of attentional bias toward negative stimuli, their interpretation must be contextualized within the sample's socioecological profile. We recommend that future studies systematically incorporate contextual variables such as exposure to violence, cultural context, emotional expression norms, and media viewing habits to better understand how socioenvironmental factors interact with attentional processing.

> *It is also important to consider the potential influence of socio-cultural and environmental factors on attentional patterns. Cultural norms surrounding emotional regulation, expression, and attentional control may modulate how individuals respond to emotional versus neutral stimuli. For instance, individuals from collectivist cultures may exhibit greater attentional disengagement from emotionally intense stimuli due to norms that discourage overt emotional expresión (85,86). Moreover, contextual variables such as growing up in high-violence environments (87,88), or differences in urban versus rural upbringing (89), may sensitize or desensitize attentional systems to negative content. Sociocultural factors likely influenced the attentional patterns observed in our sample.*
>
> *Considering this, it is possible that Colombia's history of armed conflict and widespread exposure to violence could have heightened sensitivity to threat-related cues. For instance, indirect exposure to neighborhood violence has been linked to increased avoidance behaviors in Colombian children (90). Additionally, while Colombia is traditionally viewed as a collectivist society, Bogotá the urban setting of this study, has been associated with more individualistic orientations (91). These contextual elements may shape emotional processing and attentional biases differently than in populations from more stable or culturally homogeneous regions. We recommend that future studies include contextual variables such as violence exposure, emotional suppression norms, and media use habits to better understand the generalizability of attentional biases across diverse populations.*

- Although the sample includes 122 participants, the absence of affective disorders may limit the results and restrict their generalizability to specific populations.

In the Constraints on Generality section, we emphasize that the exclusion of participants with affective disorders was a deliberate methodological decision, based on limitations observed in previous studies such as those of Nummenmaa et al. (2006), Duque and Vázquez (2015) and Sears et al. (2019), where this factor was not controlled. This control allowed us to isolate the attentional mechanisms characteristic of the general population, increasing the internal validity of the study and

reducing biases in the interpretation. We believe that this approach is a strength of our design, as it provides a more accurate baseline for future clinical comparisons. Furthermore, as indicated in the Prospects for Future Studies section, we propose replicating this paradigm in clinical samples and comparing these results with the general population to advance our understanding of the attentional biases associated with affective disorders.

In light of this, we have added the following information to the Constraints on Generality section (p. 29-30).

> *In this study, we excluded participants with affective disorders. While this may limit generalizability to clinical populations, it directly addresses a limitation present in previous studies, where the inclusion of participants with undetected affective symptoms may have introduced confounding effects in attentional patterns. For example, Nummenmaa et al. (19), Duque and Vázquez (41) and Sears et al. (31) either included clinical samples or did not control for the presence of affective symptoms, making it difficult to determine whether attentional biases reflect general population mechanisms or are specific to individuals with mood disorders. By ensuring a non-clinical sample, we are able to isolate attentional mechanisms characteristic of the general population, improving internal validity and reducing interpretation bias. This allows for a clearer understanding of baseline attentional responses to emotional and non-emotional stimuli.*

Additionally, we added the following information to the Prospects for future studies section (p. 33).

> *For future studies, it is recommended to replicate this study with people without affective disorders outside the pandemic context and further investigate the emotional regulation hypothesis. In addition, they studies should consider replicating this paradigm with clinical populations, such as individuals diagnosed with depression or anxiety, to examine whether the attentional patterns observed here differ significantly from those of nonclinical groups. Such comparisons could help clarify whether the attentional bias toward negative stimuli reflects a greater capacity for emotional regulation or vulnerability to affective dysregulation. Exploration of these dynamics in different populations would contribute to both theoretical refinement and practical applications in clinical settings.*

Reviewer #2:

We sincerely thank the reviewer for the time and effort dedicated to reviewing our manuscript. We are especially grateful not only for the critical and constructive comments but also for the thoughtful suggestions provided to address each identified issue. These recommendations facilitated our revision process and helped us to better understand and communicate the core problems in our manuscript. Thanks to their input, we were able to refine key sections of the paper, including the theoretical framing, ethical justification, statistical analysis, and discussion.

Abstract:

The abstract tries to summarize the study but ends up being a bit fragmented. The key findings aren't presented clearly, and the statistical results lack context. The gender differences and interaction effects, which seem important, are mentioned without proper emphasis. There's

also no acknowledgment of the study's limitations, especially the ethical concerns about covert consent.

Suggestion: **Rewrite the abstract to make the findings more direct and highlight key statistical outcomes and their relevance. A brief nod to the limitations would also help set expectations.**

Taking into account these recommendations, as well as some suggestions made by Reviewer 1, we have completely rewritten the abstract, adding the most relevant results, as well as the limitations and suggestions for future studies (p. 1). This is the abstract now:

> *The attentional system tends to prioritize negative stimuli in the early stages of processing, favoring threat detection. However, it is unclear whether this bias is maintained or reversed toward positive stimuli at later stages. In this study, we used a free-viewing paradigm with eye tracking to examine early and late attentional biases toward negative, positive, and neutral stimuli (humans in emotionally unloaded activities) versus control stimuli (inanimate objects) in 122 participants without affective disorders (64 men, 58 women). Mixed models evidenced that participants fixated faster ($F3, 15156.24 = 186.43, p < .001$) and more first times ($F3, 15157.50 = 56.93, p < .001$) on negative stimuli compared to the other stimuli, confirming a negativity bias in early attention. In late attention, we observed a longer total fixation time on negative stimuli ($F3,15178.97 = 451.78, p < .001$) and a greater total number of fixations ($F3, 15155.66 = 447.39, p < .001$), contradicting the hypothesis of a positive bias in emotional regulation. In addition, we found significant differences in the interaction between emotional content and gender in delayed attention ($p < .001$), although no direct differences were found between women and men. This sustained negativity bias may reflect an adaptive regulatory process in which individuals allocate attentional resources to threat-related information to enhance learning and emotional preparedness. One limitation of the study is the lack of control over socioecological factors that may shape attentional biases, such as cultural normalization of violence. Future studies should examine these effects across diverse sociocultural settings and in clinical populations.*

Introduction:

The introduction sets the stage but feels repetitive in places and doesn't flow smoothly. While some relevant studies are cited, their connection to the research question isn't always clear. The rationale for using eye-tracking technology is underdeveloped, and the research gap isn't strongly emphasized.

***Suggestion*: Streamline the theoretical framework to remove redundancy and sharpen the focus on the research question. Be more explicit about what makes this study different from previous work.**

We have made significant revisions to improve clarity, eliminate redundancies, and optimize the logical flow of ideas. First, we refined the theoretical framework by integrating clear definitions of key concepts such as attentional bias and inhibitory processes, so that these terms are explicitly linked to the study's objectives. We also strengthened the justification for using eye-tracking by explaining its advantages over reaction-time-based methods in capturing both early and late stages of attention (p. 5).

Early studies demonstrated that emotional stimuli both positive and negative capture attention more quickly than neutral stimuli (16,19). However, subsequent research indicated that negative stimuli dominate early attention (39), whereas positive stimuli generate greater late attention (27,29,31).

Also, we emphasized how our study builds on previous research while addressing key methodological limitations, particularly the need to control for the presence of human figures in neutral conditions (p. 5)

Despite these findings, previous studies often lacked direct competition between positive and negative stimuli or did not control for confounding factors such as the presence of humans in neutral images (31,40). Addressing these methodological limitations, by concurrently presenting positive, negative, and neutral stimuli while controlling for human-related content in neutral images, can provide a more accurate and ecologically valid assessment of natural attentional biases in non-clinical populations.

Furthermore, we have clarified the research gap by highlighting the scarcity of studies examining early and late attentional biases in nonclinical populations, especially those that also consider the social relevance of emotional stimuli in their design. More than 70% of the introduction has been rewritten, and in an effort to be more specific, it has been reduced from 1,587 words to a total of 1,082 words, a reduction of ~30%.

Methods:

Participants: The sample size is described, but there's no formal power analysis to justify it.

This is a crucial point. We have added the following paragraph to our article (p. 7), where we acknowledge this limitation. Furthermore, we performed a power analysis to show that our sample size provides an adequate power considering estimates based on other research (see also the response to a related point raised by Rev. 1).

Given the within-subject design and repeated-measures structure of the eye-tracking data, we expect high sensitivity for detecting attentional biases. However, we acknowledge the limitation of our study and the importance of power analyses in study planning, and encourage future research to incorporate a priori power calculations to further strengthen the robustness of the findings. Although we acknowledge this limitation, a post hoc power analysis was performed on the data from the Sears et al. (51) , the only previous study that presents 4 simultaneous images, using the pwr package in R. The analysis revealed that, with $u = 2$ degrees of freedom in the numerator and $v = 78.03$ degrees of freedom in the denominator, the effect size was $f^2 = 0.12$. Given a significance level of $\alpha = 0.05$, an adequate probability of detecting significant effects of 0.80 would be achieved with a sample of 81 participants. Based on these assumptions, our sample size of $n = 122$ provides a power of 0.94.

Stimuli Selection: The process of selecting and validating stimuli lacks detail. It's unclear whether cultural relevance was considered.

The description of the stimuli selection process has been expanded (p. 8-9). Although it was previously noted that the stimuli were validated, we have now clarified in the supplementary material that they were evaluated in two dimensions and by a comparable population. The following information has been added to the manuscript:

> *Initially, 71 stimuli were preselected from the Nencki Affective Picture System (NAPS) (54) and Open Affective Standardized Image Set, OASIS (55) image banks and validated with a population similar to the one evaluated (228 participants). Arousal and valence were evaluated for the stimuli, and those that maintained the evaluation in both dimensions were used for the experiment.*
> *Subsequently, 16 stimuli were selected from each category (64 in total). For more details, see section 1.1 in the Supplementary Material. For the construction of the 32 trials with the four competing stimuli, 16 trials were first constructed with the 64 images obtained, each of which contained one randomly selected image of each of the four types of stimuli (Positive, negative, control, and neutral).*
> *Subsequently, another 16 trials were conducted with the same 64 stimuli in the same manner, but without allowing images that were presented together in the first 16 created stimuli to appear together again. To ensure that the stimuli were not in the same disposition, the location of the four stimuli types was equally likely to appear in each corner (Fig. 1):*

Fig 1

Presentation of experimental paradigm stimuli

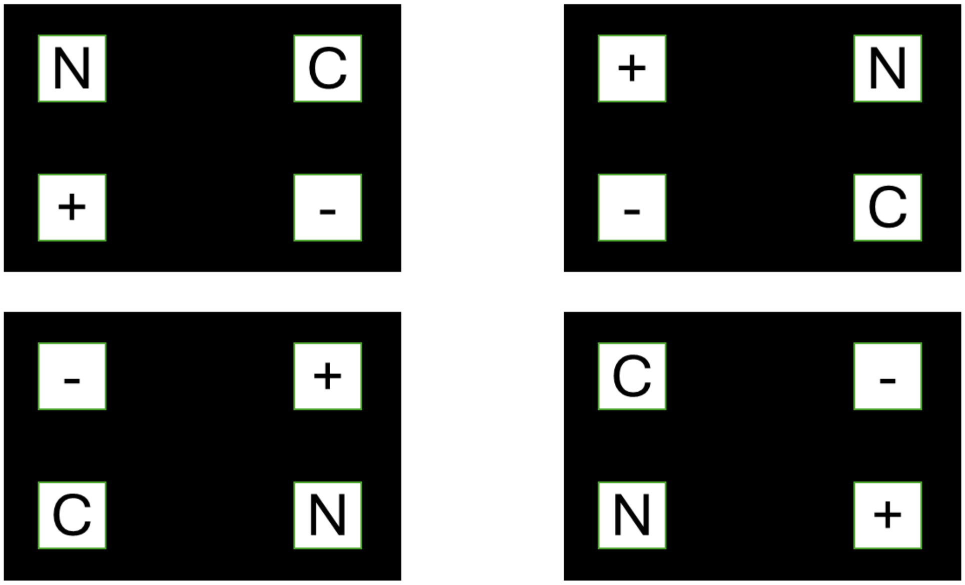

Ethics: The covert consent process raises serious ethical concerns. Even if this approach received approval, a stronger justification is needed in the text

This is a very important issue, also highlighted by reviewer 1. Considering this, we expanded the information on the use of covert informed consent (p. 14-15), to explain its importance for research and supporting its use with an article by Riege et al. (2021) that discusses the ethical implications of this method, and serves as a precedent for our study.

The study protocol was approved by the institutional ethics committee of Universidad El Bosque (PIS 002-2020). A first informed consent form was submitted virtually, in order to obtain preliminary data that would allow us to discard those participants who did not meet the inclusion criteria of the research. Subsequently, participants who met the criteria reported to the laboratory the following day. In the laboratory, participants signed a covert written consent form, in which participants were not initially informed that their eye movements were being recorded. Personal data were treated confidentially and anonymously to ensure privacy and compliance with data protection regulations. Only aggregated data were used in the analyses and no personally identifiable information was retained. Once the experimental paradigm was completed, full informed consent was provided, explaining the experiment in detail and, in the event of not wishing to participate, ensuring the deletion of all data collected.

We recognize that the use of covert consent procedures poses significant ethical challenges. However, in the context of this study, this approach was deemed necessary to ensure the scientific validity of the results while minimizing any potential risk to participants. Throughout the process, specific measures were taken to safeguard the autonomy of the participants and to respect ethical standards.

In order not to disrupt natural patterns of visual scanning, participants were not initially informed that their eye movements were being recorded. This temporary omission was approved by the institutional ethics committee of Universidad El Bosque (approval number PIS 002-2020), based on the following considerations: (1) the risk to participants was minimal, as only gaze patterns were collected in response to emotionally varied but noninvasive visual stimuli, and no personal biometric data beyond gaze location were recorded; (2) a thorough debriefing and full informed consent was conducted immediately after the task, during which participants were given the opportunity to withdraw their data without penalty; and (3) early disclosure of the specific study objectives would likely have compromised the validity of the data by altering participants' naturalistic visual behavior.

This approach aligns with recent methodological perspectives that support the ethical use of covert eye-tracking procedures under controlled conditions, particularly when transparency may compromise the authenticity of participants' responses. For example, Riege et al. (59) argue that covert eye-tracking can be ethically justified when accompanied by robust debriefing protocols and voluntary data withdrawal options, as it allows researchers to investigate cognitive and attentional processes without introducing reactivity or demand characteristics.

In applying this design, we attempted to balance the need to reduce experimental bias with the ethical imperative to respect participants' rights. No participants expressed discomfort or chose to withdraw after receiving full information about the study, suggesting that the measures taken were effective in maintaining both scientific integrity and ethical responsibility.

Statistical Analyses: The use of Linear Mixed Models (LMM) is appropriate, but many assumptions were violated. Bootstrap techniques were applied, but the results are inconsistently emphasized.

This is a fair point and we thank the reviewer for highlighting this lack of consistency in the interpretation. We explaining the importance of the technique used and how to interpret such data (p. 12-13).

> *We used bootstrap techniques to address violations of statistical assumptions, particularly regarding the normality of residuals in the mixed-effects models. Traditional parametric inference based on these models could have led to biased conclusions if assumption violations were ignored. To mitigate this, we applied non-parametric bootstrapping with 10,000 iterations, which allowed us to generate empirical confidence intervals (CIs) for all fixed effects. Statistical inference was based on whether these CIs included zero: effects whose 95% Cis bounds did not cross zero were interpreted as statistically significant. For all main results, these bootstrap-based inferences were consistent with the pattern of significant and non-significant results. Therefore, although assumption violations were present, the convergence between both inferential approaches supports our findings. Thus, although inference based on CIs is preferable, we decided to report p-values in the main text as they are more widely used, but this interpretation is fully supported by the bootstrap-based CIs.*

Results:

The assumption violations (e.g., non-normal residuals) are concerning, and while bootstrap techniques were used, the findings are not always interpreted with this in mind.

We added an explanation in the "Statistical Analyses" section for correct interpretation of the data.

The marginal R^2 values are very low, suggesting the fixed effects (stimulus type, gender) explain little variance

We have added the following paragraphs in the Results section.

Early attention (p. 17):

> *The marginal R^2 values were relatively low, indicating that the fixed effects explain a modest proportion of the variance. This highlights the importance of individual differences and stimulus variability, captured by the conditional R^2 values.*

Late attention (p. 20):

> *As in early attention, the marginal R^2 is low indicating that, although the independent variables explain little variance in response, the effects found are consistent and significant. Thus, it is suggested that there may be other potentially individual or contextual factors influencing attentional patterns.*

Additionally, in the discussion we have added the following paragraph (p. 22-23):

> *Furthermore, the models produced low marginal R^2 values, indicating that the fixed effects (stimulus content and gender) accounted for only a small portion of the total variance. While at first this might suggest weak explanatory power, these findings should not be overlooked. The low marginal R^2 values likely reflect the inherent complexity of attentional behavior, which is probably influenced by numerous unmeasured individual, contextual, and situational factors. Importantly, the detection of statistically significant effects, even with low marginal R^2, supports the consistency of the emotional stimuli's influence across participants. The much higher conditional R^2 values, which incorporate variance explained by random effects, further highlight the substantial contribution of individual- and stimulus-level variability in attentional patterns. Importantly, the presence of statistically significant effects, even with low marginal R^2, supports the robustness of our design and the consistency of the emotional stimuli's influence across participants.*

Gender and stimulus interactions are presented but lack depth in interpretation.

We would like to clarify this point that was probably poorly explained in the previous version. Although an interaction between gender and stimulus content was observed, no differences emerged when comparing stimuli men and women within the same stimulus category. That is, comparisons between men and women for positive, negative, neutral or control stimuli separately showed no differences. Instead, the interaction reflects differences that arise when comparing gender across different emotional categories; for example, men viewing positive stimuli compared to women viewing negative stimuli. To clarify this subtle but important issue, we added the following paragraphs in the Results.

Early attention (p. 18):

> *To further clarify the interaction between gender and stimulus content, we examined pairwise comparisons within and across stimulus categories. No significant gender differences were observed when comparing men and women within the same stimulus type (e.g., men vs. women for negative stimuli). However, the interaction effect emerged when comparing different combinations of gender and stimulus type (for example, comparing fixations on negative stimuli in women to fixations on positive stimuli in men). This indicates that gender differences in attentional allocation are context-dependent and shaped by the emotional content of the stimuli rather than by gender alone.*

Late attention (p. 20):

> *As in early attention, the marginal R^2 is low indicating that, although the independent variables explain little variance in response, the effects found are consistent and significant. Thus, it is suggested that there may be other potentially individual or contextual factors influencing attentional patterns.*

Some figures are cluttered and difficult to interpret.

In order to make the graphs clearer, we kept the violins that explain the distribution of the data, but we removed the distribution by means of the geom_jitter function.

Discussion:

The discussion has a tendency to drift into speculation, especially when trying to explain the findings through cognitive reappraisal.

This is an important point. We have rewritten this section to avoid speculating in this regard. As a result, we now presented it merely as a hypothesis that needs to be explored in future studies (p. 28)

> *One hypothesis we propose to try to explain the findings is that emotional regulation strategies, such as cognitive reappraisal, may help individuals to reinterpret and process threatening stimuli more adaptively. Cognitive reappraisal allows individuals to engage with emotional stimuli without avoiding them, leading to a more effective response (76–78). This strategy may promote sustained attention toward threatening stimuli when these are not perceived as real threats, such as in the case of the stimuli presented in our study. Just like how people might watch horror movies or observe situations in the street that don't directly affect them, they may remain engaged with these stimuli as a means to learn and prepare for potential threats. This process allows individuals to face and learn from simulated threats, which can be more adaptive for future situations. While this framework provides a plausible but tentative explanation for our findings, further replication studies are needed to identify whether this effect is larger in other populations.*

Contradictory results, like those challenging the positivity bias hypothesis, are mentioned but not fully explored.

We have expanded the discussion to more thoroughly address the contradiction with the positivity bias hypothesis. In particular, we now incorporate an explanatory framework based on adaptive learning and threat preparedness, proposing that sustained attention to negative stimuli may serve a functional role in enhancing situational awareness and emotional regulation (p. 25-26).

> *This finding challenges the positivity bias hypothesis, which would predict a preference for viewing stimuli with positive emotional content over negative and neutral stimuli, particularly during later stages of information processing in non-clinical populations. Our results contrast with findings such as those of Isaacowitz et al. (29), who reported that older adults tended to allocate more attention to positive stimuli over time. However, the younger, non-clinical sample in our study, coupled with the simultaneous presentation of emotionally competing stimuli, likely heightened vigilance toward threat-related cues. Rather than supporting a generalized positivity bias, our findings underscore the conditional nature of this effect. One plausible explanation is that sustained attention to negative stimuli may serve as an adaptive function by facilitating learning and preparedness in response to potential threats, similar to the cognitive engagement elicited by suspenseful or dramatic scenes. This aligns with theoretical accounts proposing that negative information may be more salient because it signals possible danger and thus*

requires greater cognitive resources. Future research should consider factors such as participant age, task demands, cultural context, and emotional salience when evaluating attentional biases, as these variables may moderate the expression of positivity or negativity biases.

But additionally, also in response to a point raised by rev. 1, we have added a tentative neuroscientific explanation to this possibility (p. 26):

Several studies suggest that sustained attention to negative stimuli may reflect an adaptive mechanism rooted in the evolutionary role of the attentional system to prioritize potentially threatening information (21,66). The amygdala, in particular, plays a key role in this process, enhancing the perceptual salience of emotionally salient stimuli and contributing to prolonged attentional engagement (67,68). This bias toward negative information has been linked to stronger autonomic and cortical responses (69), which may explain the sustained fixation observed in our findings.

Also, we have added the following information on emotional states changes as a result of the pandemic in the section Constraints on Generality (p. 30-31):

It is particularly important to consider that our data were collected during the second year of the COVID-19 pandemic, a time in which emotional dysregulation, elevated depressive symptoms, and increased vigilance towards threat-related stimuli were widely documented in the general population (80,81). The heightened attentional focus on negative stimuli may partly reflect these contextual stressors. Pandemic-related uncertainty and media exposure may have amplified attentional biases to threatening cues, making our findings particularly relevant to understanding attentional dynamics under prolonged stress.

In addition, to discuss external factors in more depth, we included the following paragraph (p. 33-34):

It is also important to consider the potential influence of socio-cultural and environmental factors on attentional patterns. Cultural norms surrounding emotional regulation, expression, and attentional control may modulate how individuals respond to emotional versus neutral stimuli. For instance, individuals from collectivist cultures may exhibit greater attentional disengagement from emotionally intense stimuli due to norms that discourage overt emotional expresión (85,86). Moreover, contextual variables such as growing up in high-violence environments (87,88), or differences in urban versus rural upbringing (89), may sensitize or desensitize attentional systems to negative content. Sociocultural factors likely influenced the attentional patterns observed in our sample. Considering this, it is possible that Colombia's history of armed conflict and widespread exposure to violence could have heightened sensitivity to threat-related cues. For instance, indirect exposure to neighborhood violence has been linked to increased avoidance behaviors in Colombian children (90). Additionally, while Colombia is traditionally viewed as a collectivist society, Bogotá the urban setting of this study, has been associated with more individualistic orientations (91). These contextual elements may shape emotional processing and attentional biases differently than in populations from more stable or culturally homogeneous regions. We recommend that future studies include contextual

variables such as violence exposure, emotional suppression norms, and media use habits to better understand the generalizability of attentional biases across diverse populations.

Conclusion:

The conclusion briefly summarizes the study but feels rushed and doesn't fully tie back to the research question or address the broader relevance of the findings.

We revised the conclusion to more directly tie the findings to our research question, showing how sustained attention to negative stimuli informs our understanding of attentional processing across stages. We also clarified the broader significance of this bias, especially in terms of adaptive vigilance in emotionally healthy individuals (p. 34).

> *This study contributes to the understanding of how the attentional system prioritizes emotional over neutral stimuli by using a naturalistic, free-viewing paradigm. Our findings revealed a sustained attentional bias toward negative stimuli during both early and late processing stages, even in a non-clinical population. These results suggest that, contrary to the positivity bias hypothesis, negative stimuli may continue to dominate attention in emotionally healthy individuals, possibly due to their relevance for adaptive vigilance and threat detection.*

There's also no acknowledgment of the study's limitations, particularly regarding ethical concerns and statistical issues.

We now address the potential influence of subclinical depressive symptoms in participants due to the pandemic context, clarify that the covert consent procedure, although potentially sensitive, was ethically approved and implemented following established guidelines to minimize bias, and highlight that the use of bootstrapping techniques is more adequate that *p*-value based significance, given that our models do not meet all assumptions. However, because inference from bootstrap Cis and p-values are consistent for all main results, we report *p*-values as this is more commonly used in the literature. We believe that these clarifications strengthen the transparency of the study's conclusions (p. 34).

> *However, some limitations should be acknowledged. First, while participants with clinical diagnoses were excluded, the inclusion of individuals with subclinical depressive symptoms possibly influenced by the COVID-19 context could have affected attentional patterns toward threat-related stimuli. Second, while the covert consent procedure could raise ethical concerns, it followed approved ethical protocols and was implemented to minimize experimental bias without compromising participant autonomy. This approach aligns with previous ethical frameworks supporting covert eye-tracking under strict debriefing and withdrawal protocols (59). Finally, although we applied bootstrapping techniques to address violations of statistical assumptions in mixed-effects models, such assumptions still limit inference to some extent.*
