# Supplementary material for: Effects of stimulus emotional content on gaze pattern: An eye-tracking study
Source: PLoS One. 2026 Jan 28;21(1):e0341261. doi: 10.1371/journal.pone.0341261 (PMC12851487; doi:10.1371/journal.pone.0341261)
Supplement: Attachment — Submitted filename: Response_to_Reviewers_auresp_2.pdf [file pone.0341261.s004.pdf]

Manuscript ID: PONE-D-24-50575
Title: *Effects of stimulus emotional content on gaze pattern: an eye-tracking study*
Dear Dr. Elisa Scerrati,

We would like to express our deep appreciation for your editorial work and sincerely thank all reviewers for their insightful feedback. Thanks to their contributions, we believe our manuscript is now more solid, methodologically robust, and comprehensive. We hope you will now consider it ready for publication.
In this revision, we have carefully addressed the critical statistical concerns raised by Reviewer #3. Following these recommendations, we have:

- Re-specified all analyses as **generalized linear mixed models (GLMMs)** using a Gamma distribution with a log link, to more appropriately model the bounded and positively skewed nature of the dependent variables.
- Included **random slopes** for within-subject predictors (e.g., stimulus content by participant) to better capture the hierarchical data structure and avoid inflation of Type I error.
- The interpretation of the results remained very close to what it was before. The changes, although minimal, simplified the interpretation of the results. The only change was in delayed attention, as the interaction between gender and stimulus content was no longer significant. This interaction had previously lacked theoretical relevance as it did not reflect differences within the same stimulus type between genders.
- Adjusted our reporting to use likelihood ratio chi-square tests for model comparisons.

We are also grateful to Reviewer #1 for their positive evaluation and recognition of the manuscript's strengths.

We are confident that these changes have strengthened the analytical rigor and interpretive clarity of our work. We respectfully submit our revised manuscript along with:

- A marked-up version highlighting all changes,
- A clean version of the revised manuscript, and
- A detailed point-by-point response to reviewers' comments.

Thank you once again for your time and commitment to ensuring the quality of the review process.
Sincerely,
Andrés Castellanos-Chacón
On behalf of all co-authors
Universidad El Bosque
gcastellanos@unbosque.edu.co

Reviewer #1:

We would like to sincerely thank the reviewer once again for their constructive comments and suggestions during the initial round of review, which greatly contributed to improving the clarity, structure, and methodological rigor of our manuscript. Their feedback helped us refine the theoretical framing and ensure that the analyses were presented in a more coherent and transparent way. We would like to express our sincere gratitude for your excellent and precise methodological recommendations.

Reviewer #3:

We thank the reviewer for their clear and helpful methodological recommendations and statistical guidance. We followed all suggestions, and the reanalysis confirmed that model assumptions were met and that the overall pattern of findings remained consistent.

1. The response time and fixation duration variables appear to be highly skewed, as is typical for time-based measures. While the use of bootstrapping is a reasonable strategy for deriving robust confidence intervals, it does not address other fundamental modeling issues, particularly those related to the link function. When dependent variables are bounded (e.g., positive times, counts), equal differences across levels of a linear predictor cannot be interpreted as reflecting equal differences on an underlying scale of the ability or construct being measured. This is primarily why generalized linear models (GLMs) should be used, apart from appropriate modeling of the error term. In the present specific case, using generalized linear (mixed) models (GLMMs), likely with a Gamma distribution and a log link, would provide a much more natural and statistically sensible approach. In this context, assumption violations are not an unfortunate surprise, but it is inherent in the data structure! GLMMs are designed to handle this. The "lme4" or the "glmmTMB" packages of R could be used for this.

Following their recommendation, we respecified Models 1, 2, 4, and 5 as generalized linear mixed models (GLMMs) with a gamma distribution and a log link, implemented using the glmmTMB R package. We maintained the use of the bootstrap method to obtain robust confidence intervals. In addition, we included ANOVA tables from the car package to facilitate the interpretation of the main effects.

This change required updates to several sections of the manuscript, including the Abstract, Statistical Analysis, Results, Discussion (particularly the subsection on delayed attention), and Conclusions.

The updated analyses yielded results consistent with those obtained previously, with one minimal change: the interaction between stimulus content and gender on delayed attention was no longer statistically significant. It's important to note that even in the previous model, this interaction lacked theoretical relevance, as it reflected differences between combinations across categories (e.g., male-negative stimulus versus female-positive stimulus), rather than within the same stimulus category across genders (e.g., male-negative stimulus versus female-negative stimulus), which would have been the theoretically meaningful comparison. Therefore, eliminating this effect in the new model simplifies the results without altering the central interpretation.
The changes that were made were as follows:

Abstract:

In the summary we explicitly leave the type of analysis we did for the different models and additionally we update the main results obtained (p. 1). This is the abstract now:

The attentional system tends to prioritize negative stimuli in the early stages of processing, favoring threat detection. However, it is unclear whether this bias is maintained or reversed toward positive stimuli at later stages. In this study, we used a free-viewing paradigm with eye tracking to examine early and late attentional biases toward negative, positive, and neutral stimuli (humans in emotionally unloaded activities) versus control stimuli (inanimate objects) in 122 participants without affective disorders (64 men, 58 women). We fitted generalized linear mixed models with random intercepts for stimuli and random intercepts and slopes for participants, and used non-parametric bootstrap resampling to obtain robust estimates and confidence intervals. Additionally, we analyzed the number of first fixations using a linear model with an ANOVA approach. Results showed that participants fixated faster ($\chi^2(3) = 97.55, p < .001$) and more frequently in their first fixation ($F_{3,15157.50} = 56.93, p < .001$) on negative stimuli compared to the other categories, confirming a negativity bias in early attention In late attention, we found longer total fixation durations ($\chi^2(3) = 200.24, p < .001$) and a greater number of fixations ($\chi^2(3) = 207.02, p < .001$) for negative stimuli, contradicting the hypothesis of a positivity shift during emotional regulation. This sustained negativity bias may reflect an adaptive regulatory process in which individuals allocate attentional resources to threat-related information to enhance learning and emotional preparedness. Future studies should examine these effects across diverse sociocultural settings and in clinical populations.

Methods:
Statistical analysis
This section clarifie the models used (p. 12) but also specifies the reason for using Bootstrap. The section read as follows:

Generalized linear mixed models (GLMM) with a Gamma distribution and a logarithmic link function were fitted for each dependent variable, except for First Fixation Count (FFC), which was analyzed using a linear model due to the nature of the variable. For early attention, the modeled variables were: Time to First Fixation (TFF), First Fixation Duration (FFD), and FFC. For late attention, the modeled variables were: Total Duration of Fixation (TDF) and Total Number of Fixations (TNF).

The main fixed effects included stimulus content and participant gender, while the random effects included intercepts for participant and stimulus ID, plus random slopes for stimulus content within participant.

To obtain robust estimates and assess the stability of effects, non-parametric bootstrap resampling with 1000 iterations was used to compute empirical confidence intervals (CIs) for the fixed effects. Statistical inference was based on whether these CIs included zero: effects whose 95% CI bounds did not cross zero were interpreted as statistically significant. In all main results, the bootstrap-based conclusions matched those obtained using conventional methods.

Results:

The tables were and the graphs were updated taking into account the new models (p. 15). Simplifying the image of late attention where before it was separated by gender, but since there was no longer an interaction between gender and stimulus content, this separation was not necessary.

Table 1.
Overall results early attention

Early attention	*TFF*		*FFD*		*FFC*	
	χ^2	*p*	χ^2	*P*	*F*	*p*
Gender	*.08*	*.78*	*1.71*	*.19*	*2.84*	*.09*
Stimulus content	*236.02*	***<.001***	*160.98*	***<.001***	*56.93*	***<.001***
*Stimulus content * gender*	*1.75*	*.62*	*3.01*	*.39*	*3.16*	***.02***

Note. Effects of Stimulus Content (Negative, Positive, Neutral, and Control) and Participant Gender (Women, Men) on Time to First Fixation (TFF), First Fixation Duration (FFD), and First Fixation Count (FFC). TFF and FFD results are from generalized linear mixed models (GLMMs) with likelihood ratio tests (χ^2), and FFC results are from an ANOVA (F). TFF model: conditional R^2 = .169, marginal R^2 = .043. FFD model: conditional R^2 = .16, marginal R^2 = .022. FFC model: R^2 = .44, adjusted R^2 = .43. Significant effects are in bold.

Table 2
Overall results late attention

Late attention	*TDF*		*TNF*	
	χ^2	*P*	χ^2	*p*
Gender	*.77*	*.38*	*1.22*	*.27*
Stimulus content	*456.14*	*<.001*	*504.87*	*<.001*
*Stimulus content * Gender*	*.84*	*.84*	*3.6*	*.31*

Note. Effects of Stimulus Content (Negative, Positive, Neutral, and Control) and Participant Gender (Women, Men) on Total Duration of Fixations (TDF) and Total Number of Fixations (TNF), analyzed using generalized linear mixed models (GLMMs) with likelihood ratio tests (χ^2). TDF model: conditional R^2 = – (could not be estimated due to negligible variance of the random effects), marginal R^2 = .104. TNF model: conditional R^2 = .31, marginal R^2 = .08. Significant effects are in bold. For full ANOVA-type tables, including degrees of freedom, see Tables S20 (TDF) and S26 (TNF) in the Supplementary Material.

Discussion:

The following sections mentioning the interaction in late attention and the importance of the use of Bootstrap were removed.

> *This could be relevant, as although we did not find differences between men and women in the pattern of late attention towards specific categories of stimuli, there was a significant interaction effect between stimulus content and gender in the two parameters of late attention. This does not rule out differences in the processing of emotional and non-emotional stimuli between men and women (p.26).*
>
> *Finally, although we applied bootstrapping techniques to address violations of statistical assumptions in mixed-effects models, such assumptions still limit inference to some extent (p. 31).*

2. Another limitation concerns the specification of the random-effects structure in the mixed models. The manuscript correctly states that random effects for participants and stimuli were included, which is appropriate. However, a closer look at the shared R code reveals that only random intercepts were modeled, without any random slopes, even for within-subject factors

such as stimulus content. This is an important omission, as random slopes for within-subject predictors (e.g., stimulus content by participant) are often necessary to properly account for the hierarchical structure and dependencies in the data. The absence of such slopes can inflate Type I error rates and lead to misestimated standard errors. Bootstrapping does not solve this problem if the underlying model specification is inadequate.

We fully agree with this observation regarding the specification of the random effects structure. In the initial analyses, only random intercepts were modeled for participants and stimuli, which did not account for potential within-subject variability in predictors. Following this recommendation, we respecified the models to include random slopes for within-subject factors, particularly stimulus content by participant.

These adjustments were implemented alongside the changes in point 1. The inclusion of random slopes did not alter the overall pattern of results. The changes made within the article are consistent with the modifications in the first point, in the summary section, method, specifically Statistical analysis, and in the Results.

As a minor point: the authors say that they used linear mixed models (LMMs), but why do they report F-statistics instead of the chi-square when performing ANOVA-like comparisons? In both LMMs and GLMMs I would expect the test statistic to be z-score for coefficients, and chi-square for model comparisons.

The way the data is presented has been modified to avoid any confusion; these changes were made in the results section. They can be seen on: p. 15 (Overall results early attention), p. 16 (Fig. Early attention), p. 18 (Overall results late attention), and p. 18 (Fig. Late attention).
