# Supplementary material for: Effects of stimulus emotional content on gaze pattern: An eye-tracking study
Source: PLoS One. 2026 Jan 28;21(1):e0341261. doi: 10.1371/journal.pone.0341261 (PMC12851487; doi:10.1371/journal.pone.0341261)
Supplement: Attachment — Submitted filename: Response_to_Reviewers_auresp_3.pdf [file pone.0341261.s005.pdf]

Manuscript ID: PONE-D-24-50575
Title: *Effects of stimulus emotional content on gaze pattern: an eye-tracking study*

Dear Dr. Elisa Scerrati,

We sincerely appreciate the opportunity to further revise our manuscript and are grateful for your continued editorial guidance. We also thank the reviewers for their constructive feedback, which has once again contributed to strengthening our work.

In this revised version, we have carefully addressed the concerns raised by Reviewer #3, which primarily focused on three aspects:

- The modeling of First Fixation Count (FFC)
- The reporting and interpretation of random effects
- The inclusion of post hoc power analyses.

Accordingly, we have implemented the following changes:

- Re-specified the First Fixation Count analysis using a COM-Poisson generalized linear mixed model (GLMM).
- Expanded the reporting of random effects, providing the variance components, standard deviations, and correlations for random intercepts and slopes.
- Removed the information about post hoc power analysis.

These revisions have been reflected in the *Abstract*, *Methods* (particularly the Statistical Analysis section), *Results*, *Discussion*, and *Limitations*. Importantly, these updates do not alter the overall interpretation of the findings but enhance the clarity and coherence of the manuscript.

We are confident that these revisions address all remaining concerns and further strengthen the manuscript in accordance with *PLOS ONE*'s methodological and reporting standards. We are resubmitting:

- A marked-up version showing all changes
- A clean revised version
- A point-by-point response to Reviewer #3.

Sincerely,

Andrés Castellanos-Chacón
On behalf of all co-authors
Universidad El Bosque
gcastellanos@unbosque.edu.co

Reviewer #3:

We would like to express our sincere gratitude for your careful re-evaluation of our manuscript and for the clarity and precision of your methodological observations. Your comments once again helped us refine the statistical modeling and improve the transparency of our analyses. Below, we provide a detailed response to each of your points.

1. Modeling of First Fixation Counts (FFC): The choice to analyze FFC using a linear model with ANOVA is not ideal or clear, given the non-normal and bounded nature of count data. A Poisson or negative binomial GLMM would be more appropriate and would ensure consistency with the analytic strategy applied to the other dependent variables. Retaining a linear model here weakens the elsewhere improved analytic rigor.

Following your recommendation, we re-specified the First Fixation Count analysis using a COM-Poisson GLMM implemented via the glmmTMB package. The updated results are now reported in Table 1 (Early Attention) and detailed in Section 2.2.3 First fixation count of the Supplementary Material. The model's conditional R^2 could not be estimated due to negligible variance in random effects, which we have explicitly stated in the manuscript. The results and interpretation remain consistent with our previous findings.

The changes that were made were as follows:

Abstract:

In the summary, we changed the type of analysis for model 3 and adjusted the values to those yielded by the new analysis (see page 1):

> The attentional system tends to prioritize negative stimuli in the early stages of processing, favoring threat detection. However, it is unclear whether this bias is maintained or reversed toward positive stimuli at later stages. In this study, we used a free-viewing paradigm with eye tracking to examine early and late attentional biases toward negative, positive, and neutral stimuli (humans in emotionally unloaded activities) versus control stimuli (inanimate objects) in 122 participants without affective disorders (64 men, 58 women). We fitted generalized linear mixed models with random intercepts for stimuli and random intercepts and slopes for participants, and used non-parametric bootstrap resampling to obtain robust estimates and confidence intervals*. Additionally, the number of first fixations was analyzed with a COM-Poisson GLMM.* Results showed that participants fixated faster ($\chi^2(3) = 97.55$, $p < .001$) *and for longer durations ($\chi^2(3) = 337.45$, $p < .001$)* on negative stimuli compared to the other categories, confirming a negativity bias in early attention In late attention, we found longer total fixation durations ($\chi^2(3) = 200.24$, $p < .001$) and a greater number of fixations ($\chi^2(3) = 207.02$, $p < .001$) for negative stimuli, contradicting the hypothesis of a positivity shift during emotional regulation. This sustained negativity bias may reflect an adaptive regulatory process in which individuals allocate attentional resources to threat-related information to enhance learning and emotional preparedness. Future studies should examine these effects across diverse sociocultural settings and in clinical populations.

Method (Statistical analysis):

In the statistical analysis section of the Method, we added a paragraph specifying the model used to measure First Fixation Count (FFC). This paragraph also includes specific information on the analysis of variable intercepts (see page 12).

> *Generalized linear mixed models (GLMMs) were fitted for each dependent variable. Time-based variables (TFF, FFD, TDF, TNF) were modeled with a Gamma distribution and a log link, while the First Fixation Count (FFC) was analyzed using a count-based GLMM with a Conway–Maxwell–Poisson (COM-Poisson) distribution, which flexibly accounts for under- or over-dispersion in count data. All models included random intercepts for participants and stimuli, and by-participant random slopes for stimulus content.*

Results:

The corresponding changes were made in Table 1 (see page 15) and in the table note (see page 16).

Table 1. Overall results early attention

Early attention	TFF		FFD		FFC	
	χ^2	p	χ^2	p	χ^2	p
Intercept	59.47	**<.001**	3266.08	**<.001**	6043.41	**<.001**
Gender	.091	.34	1.73	.19	2.13	.14
Stimulus content	97.55	**<.001**	67.88	**<.001**	158.86	**<.001**
Stimulus content * gender	1.75	.62	3.01	.39	9.03	**.03**

Note. Effects of Stimulus Content (Negative, Positive, Neutral, and Control) and Participant Gender (Women, Men) on Time to First Fixation (TFF), First Fixation Duration (FFD), and First Fixation Count (FFC). TFF and FFD results are from generalized linear mixed models (GLMMs) with likelihood ratio tests (χ^2), and FFC results are from a COM-Poisson GLMM (Type III Wald χ^2 tests). TFF model: conditional R^2 = .169, marginal R^2 = .043. FFD model: conditional R^2 = .16, marginal R^2 = .022. FFC model: conditional R^2 = – (could not be estimated due to negligible variance of the random effects), marginal R^2 = .094. Significant effects are in bold.

2) Reporting of random effects: Although the revised manuscript states that random slopes have been included, the results provide no information on the magnitude of random intercepts and slopes (e.g., variance components, standard deviations, or correlations between intercepts and slope). Without these details, it is difficult to evaluate whether the inclusion of random slopes substantively contributed to the model, or whether they accounted for any meaningful variance (perhaps they were a necessary try to make, but actually useless). Providing this information would make the hierarchical modeling more transparent and convincing. (I think

that in R functions like parameters::model_parameters() or sjPlot::tab_model() or other similar would do it).

We have now addressed this point comprehensively. For each GLMM, we extracted and reported the standard deviations, variances, and correlations of the random intercepts and slopes (Tables S5, S13, S20, S28, S35) in the Supplementary Material). Additionally, the Results section now summarizes these values, highlighting their relevance to participant-level variability and model stability.

The changes that were made were as follows:

Method (Statistical analysis):

In the statistical analysis section of the Method, we added a paragraph specifying the model used to measure First Fixation Count (FFC). This paragraph also includes specific information on the analysis of variable intercepts (see page 12).

> *Generalized linear mixed models (GLMMs) were fitted for each dependent variable. Time-based variables (TFF, FFD, TDF, TNF) were modeled with a Gamma distribution and a log link, while the First Fixation Count (FFC) was analyzed using a count-based GLMM with a Conway–Maxwell–Poisson (COM-Poisson) distribution, which flexibly accounts for under- or over-dispersion in count data. All models included random intercepts for participants and stimuli, and by-participant random slopes for stimulus content. Variance components and correlations among random effects are reported.*

Results:

In the early intervention section (see page 16), we added a paragraph explaining the main findings for the three models. We clarified that Model 3 (FFC) yielded a singular fit due to negligible random-effect variance. Nonetheless, we retained the random structure required by the design. Current recommendations in hierarchical modeling emphasize that singular fits particularly when the estimated variance of a random component approaches zero, do not necessarily indicate model misspecification, but rather reflect limited empirical variability in that specific dataset. Following the guidance outlined by Gelman and Brown (2024) and the extended discussion in Gelman (2025), maintaining the theoretically justified random slopes is preferable to removing them, as omitting required random effects can bias fixed-effect estimates and lead to anticonservative inferences in repeated-measures or clustered designs. For this reason, the full random structure was preserved in Model 3 even though the variance component was small, ensuring that the model remained consistent with the study design and with contemporary recommendations for mixed-effects modeling.

> *Random effects captured meaningful participant-level variability. For TFF (Model 1, see details in Table S5 in the Supplementary Material) and FFD (Model 2, see details in Table S13 in the Supplementary Material), random intercepts and slopes for stimulus content showed moderate variance (SD = 0.13–0.36) and strong negative correlations between intercepts and slopes (r = −0.66 to −0.84), suggesting that participants with faster fixation tendencies exhibited greater differentiation between stimulus types. In contrast, the FFC (Model 3, see details in Table S20 in the Supplementary Material) model was singular, with*

negligible random-effect variance, indicating limited participant-specific variability for this measure.

Gelman, A. (2025, enero 23). When estimating a treatment effect with a cluster design, you need to include varying slopes, even if the fit gives warning messages. *Statistical Modeling, Causal Inference, and Social Science.* https://statmodeling.stat.columbia.edu/2025/01/23/slopes/

Gelman, A., & Brown, N. J. L. (2024). How Statistical Challenges and Misreadings of the Literature Combine to Produce Unreplicable Science: An Example From Psychology. *Advances in Methods and Practices in Psychological Science, 7*(4), 25152459241276398. https://doi.org/10.1177/25152459241276398

Similarly, we did so in delayed care, where we explained the main findings (see page 19).

Random effects revealed meaningful individual variability. In the TDF model (Model 4, see details in Table S28 in the Supplementary Material), variability was highest for the random slopes of positive and control stimuli (SD = 0.28 and 0.26), with moderate intercept variability (SD = 0.25). In the TNF (Model 5, see details in Table S35 in the Supplementary Material), a similar pattern emerged (SDs = 0.21 and 0.20 for positive and control stimuli; intercept SD = 0.23). Stimulus-level variance was negligible in both models.

3) Post hoc power analysis: The added justification based on post hoc power calculations contributes little to the overall evaluation of the study. Post hoc power is generally not informative, and presenting it as evidence of adequacy may be misleading. It would be preferable to simply acknowledge that the sample size is relatively large compared to similar studies, while noting the absence of an a priori calculation as a limitation.

The post hoc power analysis has been removed from the "Participants" section. There was already a paragraph acknowledging that no a priori power analysis was performed and that the sample size was selected based on comparability with previous studies in this field.

The changes that were made were as follows:

Participants (Method):

The following information was removed from page 8:

Although we acknowledge this limitation, a post hoc power analysis was performed on the data from the Sears et al. (51) , the only previous study that presents 4 simultaneous images, using the pwr package in R. The analysis revealed that, with u = 2 degrees of freedom in the numerator and v = 78.03 degrees of freedom in the denominator, the effect size was f^2 = 0.12. Given a significance level of α = 0.05, an adequate probability of detecting significant effects of 0.80 would be achieved with a sample of 81 participants. Based on these assumptions, our sample size of n = 122 provides a power of 0.94.

Discussion:

A new paragraph has been added to the "Conclusions" section as a limitation, noting that while the sample size was sufficient to ensure model stability, future research should include a priori power calculations to ensure optimal sensitivity (see page 32):

> *An important limitation was the size of our sample, which, although larger than in comparable studies and providing a stable estimate of the model, lacks a priori power analysis. Future research should include a priori power calculations to ensure adequate sensitivity and further validate the robustness of these findings.*
